# Tumour inflammasome-derived IL-1β recruits neutrophils and improves local recurrence-free survival in EBV-induced nasopharyngeal carcinoma

**DOI:** 10.1002/emmm.201201569

**Published:** 2012-10-15

**Authors:** Lih-Chyang Chen, Li-Jie Wang, Nang-Ming Tsang, David M Ojcius, Chia-Chun Chen, Chun-Nan OuYang, Chuen Hsueh, Ying Liang, Kai-Ping Chang, Chiu-Chin Chen, Yu-Sun Chang

**Affiliations:** 1 Chang Gung Molecular Medicine Research Center, Chang Gung UniversityTaoyuan, Taiwan; 2 Graduate Institute of Basic Medical Sciences, Chang Gung UniversityTaoyuan, Taiwan; 3 Department of Radiation Oncology, Chang Gung Memorial Hospital at Lin-KouTaoyuan, Taiwan; 4 Center for Molecular and Clinical Immunology, Chang Gung UniversityTaoyuan, Taiwan; 5 Health Sciences Research Institute and Molecular Cell Biology, University of CaliforniaMerced, CA, USA; 6 Department of Pathology, Chang Gung Memorial Hospital at Lin-KouTaoyuan, Taiwan; 7 Department of Otolaryngology, Chang Gung Memorial Hospital at Lin-KouTaoyuan, Taiwan

**Keywords:** cancer, inflammasome, neutrophil, prognosis, therapy

## Abstract

Inflammasomes sense infection and cellular damage and are critical for triggering inflammation through IL-1β production. In carcinogenesis, inflammasomes may have contradictory roles through facilitating antitumour immunity and inducing oncogenic factors. Their function in cancer remains poorly characterized. Here we show that the NLRP3, AIM2 and RIG-I inflammasomes are overexpressed in Epstein-Barr virus (EBV)-associated nasopharyngeal carcinoma (NPC), and expression levels correlate with patient survival. In tumour cells, AIM2 and RIG-I are required for IL-1β induction by EBV genomic DNA and EBV-encoded small RNAs, respectively, while NLRP3 responds to extracellular ATP and reactive oxygen species. Irradiation and chemotherapy can further activate AIM2 and NLRP3, respectively. In mice, tumour-derived IL-1β inhibits tumour growth and enhances survival through host responses. Mechanistically, IL-1β-mediated anti-tumour effects depend on infiltrated immunostimulatory neutrophils. We show further that presence of tumour-associated neutrophils is significantly associated with better survival in NPC patients. Thus, tumour inflammasomes play a key role in tumour control by recruiting neutrophils, and their expression levels are favourable prognostic markers and promising therapeutic targets in patients.

## INTRODUCTION

Inflammasomes are multiprotein complexes consisting of the pattern recognition receptors NLRP3, NLRC4, AIM2 or RIG-I; the adaptor protein ASC; and caspase-1. Inflammasome assembly leads to caspase-1 activation, which cleaves intracellular pro-IL-1β into secretable IL-1β. Inflammasomes are activated by pathogen-associated molecular patterns (PAMPs) including viral DNA (Rathinam et al, 2010) and RNA (Poeck et al, 2010), and damage-associated molecular patterns (DAMPs) including extracellular ATP (Mariathasan et al, 2006) and reactive oxygen species (ROS; Dostert et al, 2008; Zhou et al, 2011). PAMPs and DAMPs are present in the tumour microenvironment of infection-associated cancers (Chen & Nunez, 2010; de Martel & Franceschi, 2009); however, the expression profile of inflammasomes in malignant cells remains unclear. In addition, the link between inflammasome function in malignant cells, on the one hand, and infection- and stress-induced inflammation and cancer, on the other, is unknown.

IL-1β is a proinflammatory cytokine and has been implicated in carcinogenesis (Apte & Voronov, 2008). IL-1β is tightly regulated by two steps: NF-κB-mediated transcriptional induction of non-secretable pro-IL-1β and inflammasome-mediated cleavage of pro-IL-1β into the secretable form, IL-1β (Bryant & Fitzgerald, 2009; Netea et al, 2009). Constitutively active NLRP3 inflammasome and IL-1β secretion were observed in late stage melanoma cells (Okamoto et al, 2010). However, blocking IL-1β signalling by a soluble truncated form of recombinant human IL-1 receptor in a phase I study of patients with relapsed acute myeloid leukaemia did not have any antileukaemic effect (Bernstein et al, 1999). In addition, IL-1β has also been shown to eliminate malignant cells by facilitating antitumour immunity and enhancing the effects of chemotherapy (Apte & Voronov, 2008).

Persistent infection is associated with 18% of cancers (Parkin, 2006). Nasopharyngeal carcinoma (NPC) is attributed to infection of nasopharyngeal epithelial cells with Epstein-Barr virus (EBV), a double-stranded DNA virus of the herpes virus family; following infection, the NPC-infected site is infiltrated with non-malignant lymphocytes (Huang et al, 1999; Shanmugaratnam et al, 1979). EBV establishes a latent infection in more than 90% of the world's population (Parkin, 2006). In tumour cells, only a limited number of viral genes, including latent membrane protein 1 (LMP1) and the EBV-encoded small RNAs, EBER1 and EBER2 are expressed. NPC is relatively rare among Caucasians, but its incidence is 20-fold higher among East Asian populations (Wei & Sham, 2005). In NPC patients treated under current guidelines, the tumour recurrence rate from residual local disease is 20–25% (Chen et al, 2008, 2011); thus, recurrence remains a major problem in NPC therapy.

Here, we report that inflammasome proteins overexpressed in tumour cells play a key role in local tumour control, by responding to stimulation from DAMPs, PAMPs and therapeutic treatment, resulting in IL-1β secretion and neutrophil recruitment. Our findings further highlight the potential for using inflammasome overexpression as an independent favourable prognostic marker for NPC, and suggest inflammasomes are promising therapeutic targets in cancers.

## RESULTS

### Overexpression of inflammasome genes in NPC

We analysed the expression of inflammasome components in NPC tumours and adjacent normal tissues using Affymetrix microchips (Chen et al, 2010b), followed by real-time PCR-(RT-PCR)-based validation in a second group of samples (Table 1). We found that 25 out of the 33 tested inflammasome-related genes were detectable by quantitative RT-PCR, and 8 of them (CIITA, NLRC4, NLRP3, NLRP7, AIM2, RIG-I, ASC and IL-1β) were overexpressed (fold change, >2) in NPC tumour samples, compared to adjacent normal samples. To search for the potentially functional genes involved in inflammasomes, we selected the overexpressed genes whose expression levels are >2 (tumour *vs.* adjacent normal tissues). The less significant *p*-values of RIG-I, NLRP3, NLRC4 and NLRP7 are likely due to the smaller sample size analysed in this Q-PCR study. We nevertheless included RIG-I (2.212-fold), NLRP3 (3.645-fold), NLRC4 (2.040-fold) and NLRP7 (3.367-fold) for further study.

**Table 1:** Gene expression profiles of various inflammasome-related components in NPC

Genes	Affymetrix HG U133 plus 2.0 set (*n* = 9)	Quantitative RT-PCR (*n* = 7)	
		
	Fold change (NPC/normal)	Fold change (NPC/normal)	*p*-value
NOD-like receptors			
CIITA	3.854	3.218	0.038^*^
NAIP	1.894	ND	
NOD1	0.664	1.736	0.230
NOD2	1.093	1.351	0.552
NLRC3	1.031	1.579	0.313
NLRC4	2.887	2.040	0.202
NLRC5	1.071	1.125	0.765
NLRP1	0.672	1.328	0.573
NLRP2	2.127	1.169	0.644
NLRP3	ND	3.645	0.226
NLRP4	ND	ND	
NLRP5	ND	ND	
NLRP6	ND	1.964	0.560
NLRP7	7.223	3.367	0.170
NLRP8	ND	ND	
NLRP9	ND	0.806	0.664
NLRP10	ND	ND	
NLRP11	ND	ND	
NLRP12	ND	ND	
NLRP13	ND	ND	
NLRP14	0.598	1.704	0.504
NLRX1	0.653	1.259	0.428
DNA receptor			
AIM2	7.238	8.974	0.038^*^
RNA receptors			
RIG-I	1.966	2.212	0.185
MDA5	2.432	1.389	0.346
LGP2	1.814	0.783	0.381
ATP receptors			
P2RX4	0.919	1.027	0.900
P2RX7	7.052	1.450	0.477
Pannexin 1	1.185	1.328	0.570
Core components			
ASC	2.838	24.061	0.042^*^
Caspase1	1.393	0.612	0.115
Substrate cytokines			
IL-1β	6.729	24.783	0.028^*^
IL-18	1.033	1.108	0.771

The relative fold change of mRNA expression between NPC and adjacent normal tissues was determined by Affymetrix microchip analysis and quantitative RT-PCR. Affymetrix microarray analysis has been previously described (Chen et al, 2010b). *p*-values were calculated by Student's *t* test. Abbreviations and symbols: ND, not detected; ^*^, statistically significant.

**Figure 1:**
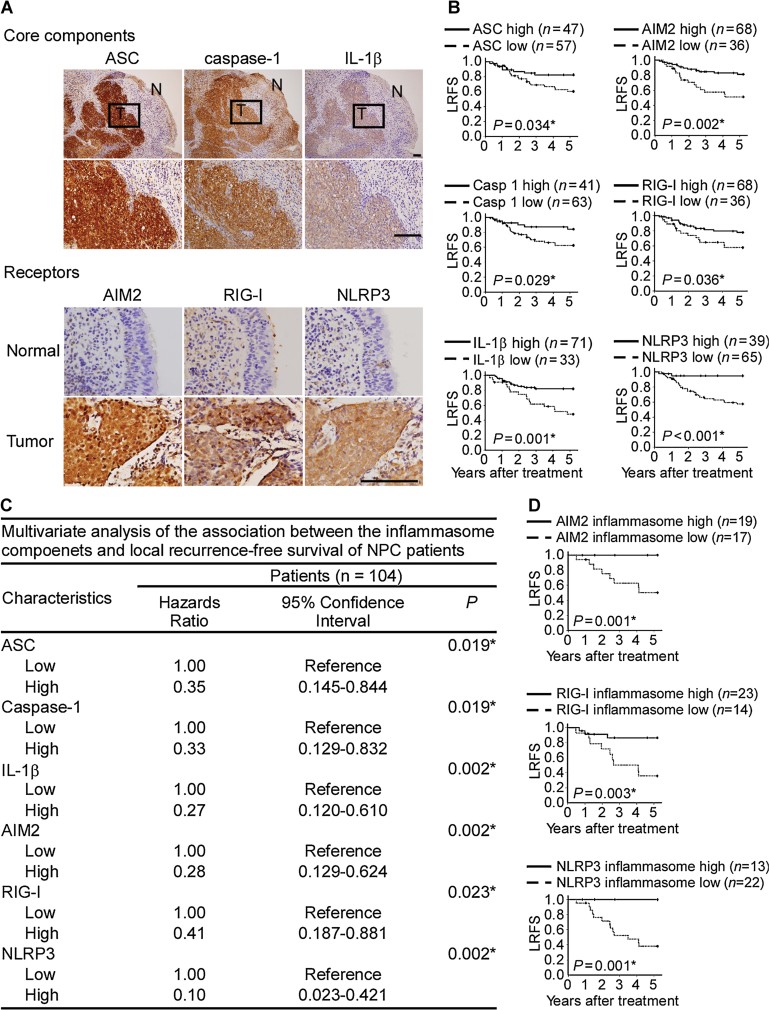
Association of AIM2, RIG-I and NLRP3 inflammasomes with better survival in NPC patients Overexpression of ASC, caspase-1 and IL-1β proteins as well as the inflammasome-receptor proteins in NPC tumour cells. Consecutive NPC tissue sections containing tumour (T) and adjacent non-tumour (N) cells were immunohistochemically stained with protein-specific antibodies. The results are shown at 100× magnification (upper panel), and the ‘N’ areas are shown at 400× magnification. The expression of these inflammasome receptors was relatively weak in the adjacent normal nasopharyngeal epithelial tissues. Bar, 100 µm.Kaplan–Meier survival analysis of LRFS as a function of elevated inflammasome component expression in NPC patients.Multivariant analysis of the association of inflammasome components with LRFS.Kaplan–Meier survival analysis of LRFS as a function of each AIM2, RIG-I and NLRP3 inflammasome in NPC patients. AIM2 inflammasome high means the levels of all four AIM2 inflammasome components including AIM2, as well as three common components ASC, caspase-1 and IL-1β in NPC biopsy tissues are scored as high levels by immunohistochemistry analyses. Similarly, RIG-I inflammasome high or NLRP3 inflammasome high means high levels of RIG-I or NLRP3 combined with high levels of ASC, caspase-1 and IL-1β. *, statistically significant as indicated.

We next used immunohistochemical staining to examine the expression of the eight inflammasome proteins together with caspase-1 in the third cohort of 104 NPC biopsy samples. Our results confirmed that these proteins were highly expressed in NPC tumour cells, but were weakly expressed in adjacent normal cells of the nasopharyngeal epithelium (Fig 1A and Supporting Information Fig S1A).

### Association of NLRP3, AIM2 and RIG-I inflammasomes with better survival in NPC patients

Kaplan–Meier survival analysis of the immunohistochemical results showed that upregulation of ASC, caspase-1, IL-1β, AIM2, RIG-I and NLRP3 correlated with better local recurrence-free survival (LRFS; Fig 1B) and disease-free survival (DFS; Supporting Information Fig S2). In contrast, the expression of CIITA, NLRC4 and NLRP7 did not correlate with survival (Supporting Information Fig S1B and S1C). No correlation was found between inflammasome expression and clinicopathological features (Supporting Information Tables S1–S6). Multivariate analysis showed that upregulation of ASC, caspase-1, IL-1β, AIM2, RIG-I and NLRP3 were strong independent prognostic predictors for better LRFS (Fig 1C and full information shown in Supporting Information Tables S7–S12) and DFS (data not shown). Notably, patients showing upregulation of all of these components in each inflammasome showed even stronger correlation with better LRFS (Fig 1D) and DFS (data not shown). None of the patients whose tumours showed upregulation of AIM2 or NLRP3 inflammasomes had local recurrence within 5 years after treatment (Fig 1D). Thus, overexpression of AIM2, RIG-I and NLRP3 inflammasomes and IL-1β in tumour cells likely contributes to local tumour control.

### Induction of pro-IL-1β by EBV LMP1 in NPC cells

**Figure 2:**
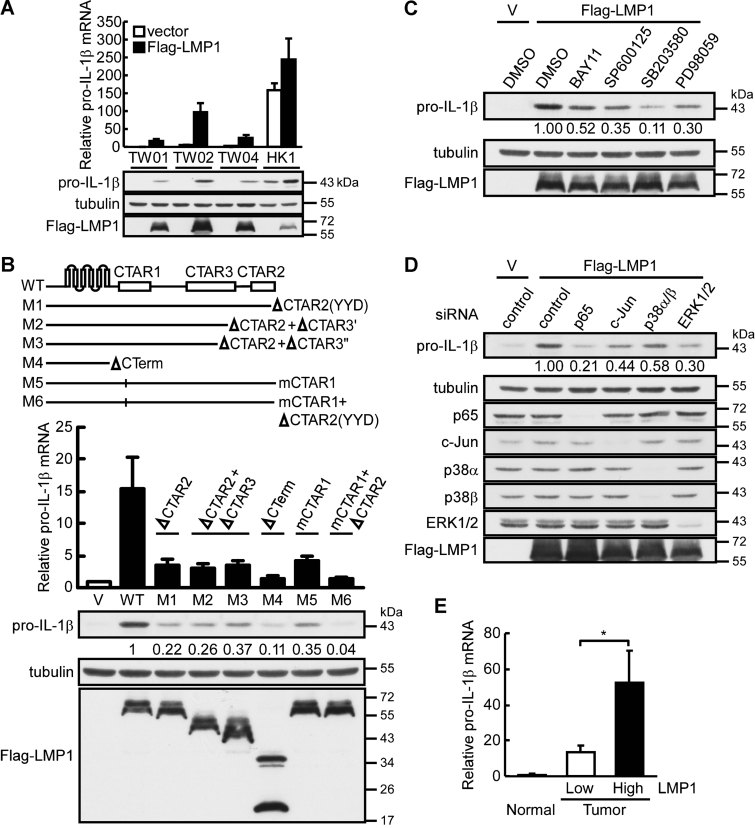
LMP1-mediated pro-IL1β induction through activation of the NF-κB and MAPK signalling pathways Induction of pro-IL-1β expression by LMP1 in NPC cell lines. NPC-TW01, -TW02, -TW04 and HK1 cells were transiently transfected with LMP1-expressing plasmid (Flag-LMP1), and pro-IL-1β mRNA and protein levels were determined at 24 h post-transfection by quantitative RT-PCR and Western blotting. All results are presented as the mean ± SD of three independent experiments.Mapping the domain of LMP1 responsible for pro-IL-1β induction. LMP1 and its domain-specific mutants were transfected into NPC-TW02 cells, and pro-IL-1β mRNA and protein levels were determined at 24 h post-transfection by quantitative RT-PCR and Western blotting. All results are presented as the mean ± SD of three independent experiments.The effect of inhibitors on pro-IL-1β induction. NPC-TW02 cells transfected with Flag-LMP1 expressing plasmid were treated with the specific inhibitors of NF-κB (BAY11-7082), JNK (SP600125), p38 MAPK (SB203580) and ERK1/2 (PD98059). The levels of pro-IL-1β protein were determined by Western blotting.The effect of p65 NF-κB, c-Jun, p38α/β MAPK and ERK1/2 knockdown on pro-IL-1β induction. NPC-TW02 cells transfected with a siRNA targeting either p65 NF-κB, c-Jun, p38α/β MAPK or ERK1/2 were further transfected with an LMP1-expressing plasmid. The levels of pro-IL-1β protein were determined by Western blotting.Correlation of LMP1 and pro-IL-1β mRNA expression in NPC biopsies. The relative fold change of LMP1 and pro-IL-1β mRNA expression between NPC and adjacent normal tissues was determined by quantitative RT-PCR. The 20 tumour tissues were divided into two groups by their relative expression levels of LMP1: high LMP1 (*n* = 10) and low LMP1 (*n* = 10). Normal tissues, *n* = 7. The results are presented as the mean ± SD and analysed by Student's *t* test. *p = 0.045.

We have reported that EBV LMP1 is expressed in NPC tumour cells (Tsai et al, 2006), which can activate NF-κB (Chen et al, 2010a; Hiscott et al, 1993) and AP-1 (Hurme & Matikainen, 1993). Since NF-κB activation is crucial for IL-1β expression (Dinarello, 2009), we hypothesized that LMP1 is an endogenous stimulator of pro-IL-1β in NPC. Thus, NPC-TW01, -TW02, -TW04 and HK1 NPC cell lines were transiently transfected with LMP1-expressing plasmid, and pro-IL-1β mRNA and protein levels were determined. As demonstrated in Fig 2A, LMP1 can significantly induce the expression of pro-IL-1β mRNA and protein in all four NPC cell lines. In order to map the domain of LMP1 responsible for pro-IL-1β induction, LMP1 and its domain-specific mutants described previously were transfected into NPC-TW02 cells (Chen et al, 2010a). According to the results shown in Fig 2B, deletion of the functional amino acids within CTAR2 (ΔYYD) or mutation of the TRAF binding motif in CTAR1 (mCTAR1) abolished LMP1-induced upregulation of pro-IL-1β by approximately 65%, compared with the induction levels achieved by the wild-type LMP1. The CTAR1 mutant combined with the CTAR2 deletion mutant (mCTAR1 + ΔYYD) or the C-terminally deleted construct (ΔCTerm) almost completely lost the ability to induce pro-IL-1β production. However, combining the inactivating mutation of CTAR2 with further deletion of CTAR3, which contains three putative JAK3 binding motifs (box1, -1′ and -2) [ΔCTAR2 + ΔCTAR3′ (Δbox2) and ΔCTAR2 + ΔCTAR3″ (Δbox1′and Δbox2)], did not cause a further decrease in LMP1-mediated activity. These experiments revealed that the CTAR1 and CTAR2 domains of LMP1 cooperate to induce pro-IL-1β expression.

Since the CTAR1 and CTAR2 domains of LMP1 are involved in NF-κB and MAPK activation (Chen et al, 2010a; Li & Chang, 2003; Zheng et al, 2007), we further determined the effect of blocking the NF-κB and MAPK signalling pathways on LMP1-mediated pro-IL-1β induction. As shown in Fig 2C and D, inhibitors of NF-κB (BAY11-7082), JNK (SP600125), p38 MAPK (SB203580) and ERK1/2 (PD98059), or knockdown of p65 NF-κB, c-Jun, p38α/β MAPK and ERK1/2 significantly abolished LMP1-induced pro-IL-1β expression. In addition, we analysed the association of LMP1 and pro-IL-1β expression in NPC biopsies by quantitative RT-PCR and found that the expression of LMP1 was significantly correlated with pro-IL-1β (Fig 2E). The results suggest that LMP1 can induce pro-IL-1β expression in NPC tumours.

### Activation of inflammasomes by PAMPs and DAMPs in NPC cells

**Figure 3:**
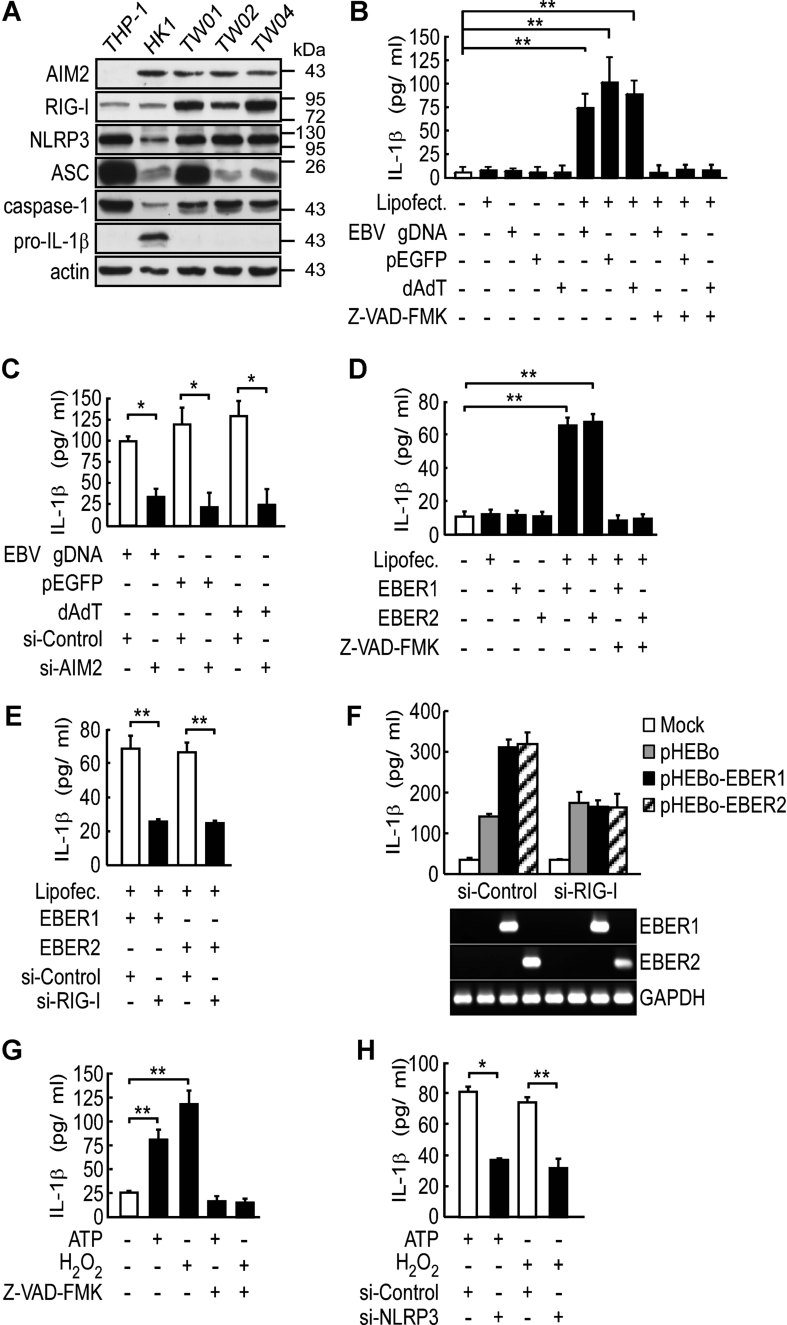
Activation of inflammasomes by EBV-associated factors and microenvironmental stressors **A.** Expression of inflammasome components in NPC cell lines. The protein levels of individual components and actin (loading control) were determined by Western blotting of cell lysates. The THP-1 cells were used as a positive control.**B,C.** IL-1β induction by EBV gDNA via AIM2. HK1 cells were transfected with fragmented EBV gDNA, pEGFP or poly (dA:dT) for 12 h, with or without a pretreatment with Z-VAD-FMK (10 µM) for 30 min (**B**), or pre-transfection with AIM2-targeting or control siRNA for 48 h (**C**). **p = 0.001, 0.004 and 0.002 for EBV gDNA, pEGFP and poly (dA:dT), respectively (**B**); *p = 0.006, 0.002 and 0.003 for EBV gDNA, pEGFP and poly (dA:dT), respectively (**C**). All results are presented as the mean ± SD of three independent experiments and analysed by Student's *t* test.**D,E.** IL-1β induction by EBERs via RIG-I. HK1 cells were transfected with *in vitro*-transcribed EBER1 and EBER2 for 12 h, with or without a pretreatment with Z-VAD-FMK (**D**), or pre-transfection with RIG-I-targeting or control siRNA for 48 h (**E**). **p = 0.006 and 0.002 for EBER1 and EBER2, respectively (**D**); **p = 0.009 and 0.009 for EBER1 and EBER2, respectively (**E**). All results are presented as the mean ± SD of three independent experiments and analysed by Student's *t* test.**F.** RIG-I activation by endogenous EBERs. HK1 cells were transfected with vector control (pHEBo), EBER1- or EBER2-expressing plasmid with pre-transfection with RIG-I-targeting or control siRNA for 48 h. Expression of EBERs was confirmed by RT-PCR. GAPDH was used as a positive control. The results are presented as the mean ± SD of three independent experiments.**G,H.** IL-1β induction by ATP and H_2_O_2_ via NLRP3. HK1 cells were treated with ATP (5 mM) for 4 h or with H_2_O_2_ (10 µM) for 24 h, with or without a pretreatment with Z-VAD-FMK (**G**), or pre-transfection with NLRP3-targeting or control siRNA for 48 h (**H**). IL-1β production was used to measure inflammasome activity. **p = 0.0001 and 0.0004 for ATP and H_2_O_2_, respectively (**G**); *p = 0.022 and **p = 0.007 for ATP and H_2_O_2_, respectively (**H**). All results are presented as the mean ± SD of three independent experiments and analysed by Student's *t* test.

We thus investigated the mechanisms through which AIM2, RIG-I and NLRP3 are activated by testing whether IL-1β secretion from NPC cell lines could be induced by stimulation with PAMPs (EBV genomic DNA, gDNA; and EBV-encoded small RNAs, EBER1 and EBER2) or DAMPs present in the tumour microenvironment (ATP and ROS), or could be affected by the caspase-1 inhibitor, Z-VAD-FMK or depletion of inflammasome components by RNA interference. After screening four NPC cell lines for expression of key inflammasome components (Fig 3A), we chose HK1 cells for the *in vitro* analysis because the basal expression level of pro-IL-1β in this cell line is similar to that in NPC biopsies (Fig 1A), and inflammasome activation was easily measured without additional induction of the signal 1, pro-IL-1β. For the other NPC cell lines, induction of pro-IL-1b was required to provide the signal 1 for the inflammasome activation. THP-1 monocytes and HEK293T cells, which have been characterized before for the presence of inflammasome components by Western blot (Martinon et al, 2002) were used as positive and negative controls for inflammasome activation, respectively (Fig 3A and Supporting Information Fig S3). We found that IL-1β release was significantly induced by treatment with fragmented EBV gDNA (average length, 500 bp) or the non-selective dsDNA, pEGFP plasmid and poly (dA:dT), and this effect was inhibited by Z-VAD-FMK (Fig 3B). However, the enhanced release of IL-1β was not observed in DNA-treated AIM2-depleted HK1 cells (Fig 3C and Supporting Information Fig S4A).

The expression of EBERs has been used to identify NPC tumour cells in our earlier studies (Tsai et al, 2006). We found that IL-1β secretion was significantly induced by *in vitro* synthesized-EBER1 and EBER2, and secretion was inhibited by Z-VAD-FMK (Fig 3D) or RIG-I depletion in HK1 cells (Fig 3E and Supporting Information Fig S4B). Further confirmation of the specificity of the effect of endogenous EBER1 and EBER2 on RIG-I-dependent IL-1β secretion was provided by transfection of the expression plasmids for EBER1 and EBER2 into HK1 cells (Gregorovic et al, 2011), indicating that endogenous EBER1 and EBER2 are able to induce IL-1β secretion, which is blocked by RIG-I depletion (Fig 3F). To test the effect of DAMPs, we measured IL-1β in the culture medium of normal and NLRP3-knockdown HK1 cells treated with ATP or an ROS inducer (H_2_O_2_). IL-1β was significantly stimulated by ATP and H_2_O_2_, and this effect could be completely blocked by Z-VAD-FMK (Fig 3G). In contrast, no stimulation of IL-1β secretion was observed in NLRP3-knockdown HK1 cells (Fig 3H and Supporting Information Fig S4C). Furthermore, we used a second NPC cell line, NPC-TW02 to examine whether inflammasome activation was the general event in NPC cells. As shown in Supporting Information Fig S5A and S5B, pro-IL-1β was inducible by TNF-α, and the inflammasome and IL-1β secretion were activated by various inflammasome stimulators, poly (dA:dT), EBER, ATP and H_2_O_2_ in NPC-TW02 cells. The interaction of endogenous AIM2, RIG-I and NLRP3 complexes with ASC upon stimulation was confirmed by co-immunoprecipitation with an ASC-specific antibody (Supporting Information Fig S6). Collectively, these findings indicate that AIM2, RIG-I and NLRP3 inflammasomes in tumour cells are activated by factors from the viral and tumour microenvironments, thus contributing to IL-1β secretion.

### Enhanced activation of inflammasomes by therapeutic treatments

**Figure 4:**
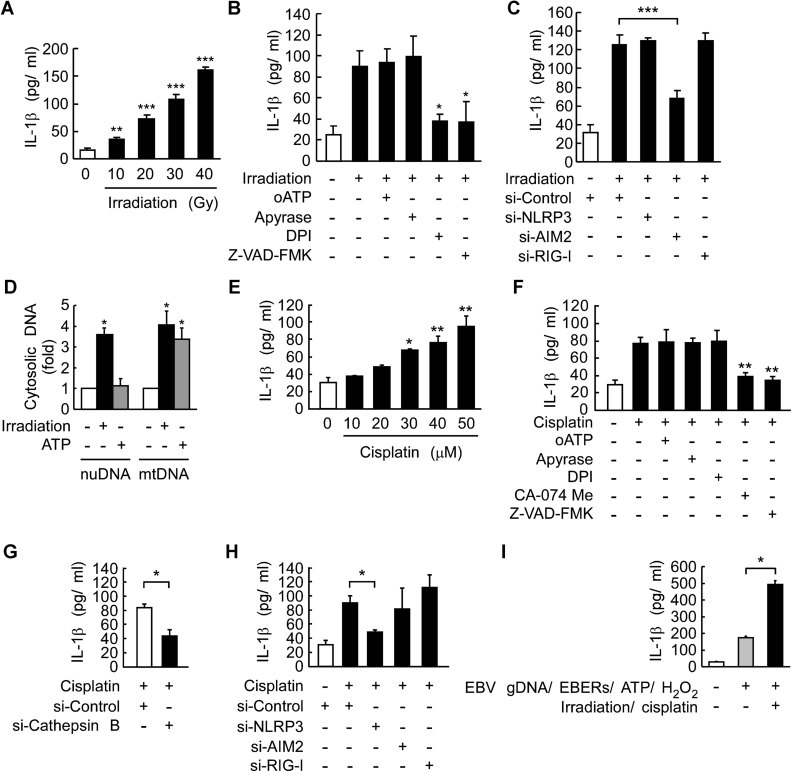
Enhanced activation of inflammasomes by therapeutic treatments Dose-dependent induction of IL-1β by irradiation. HK1 cells were treated with various doses of irradiation for 24 h. **p = 0.001; ***p = 2.7E−05, 9.8E−06 and 9.7E−08 for 20, 30 and 40 Gy, respectively. All results are presented as the mean ± SD of six independent experiments and analysed by Student's *t* test.ROS production is required for the induction of IL-1β by irradiation. HK-1 cells were irradiated (30 Gy) with or without a 30 min pretreatment with oATP (100 µM), apyrase (2.5 unit/ml), DPI (10 µM) or Z-VAD-FMK. *p = 0.02 and 0.035 for DPI and Z-VAD-FMK, respectively. All results are presented as the mean ± SD of three independent experiments and analysed by Student's *t* test.Requirement of AIM2 for irradiation-induced IL-1β production. HK-1 cells transfected with NLRP3-targeting, AIM2-targeting, RIG-I-targeting, and control siRNA for 48 h were irradiated. ***p = 0.0003, all results are presented as the mean ± SD of three independent experiments and analysed by Student's *t* test.Quantitative PCR analysis of cytosolic DNA in irradiation-treated HK1 cells. HK1 cells were treated with irradiation (30 Gy) for 24 h or ATP (5 mM) for 4 h. Nuclear DNA and mitochondrial DNA were indicated as nuDNA and mtDNA, respectively. *p = 0.005 (nuDNA analysis); *p = 0.016 and 0.017 for irradiation and ATP, respectively (mtDNA analysis). All results are presented as the mean ± SD of three independent experiments and analysed by Student's *t* test.Dose-dependent induction of IL-1β by cisplatin. HK1 cells were treated with various doses of cisplatin for 24 h. *p = 0.012. **p = 0.007 and 0.003 for 40 and 50 µM, respectively. All results are presented as the mean ± SD of three independent experiments and analysed by Student's *t* test.Requirement of cathepsin B activity for the induction of IL-1β by cisplatin. HK-1 cells were incubated with 40 µM cisplatin with or without a 30 min pretreatment with oATP, apyrase, DPI, CA-074 Me (10 µM) or Z-VAD-FMK. **p = 0.0017 and 0.0005 for CA-074 Me and Z-VAD-FMK, respectively. All results are presented as the mean ± SD of three independent experiments and analysed by Student's *t* test.Requirement of cathepsin B for cisplatin-induced IL-1β production. HK-1 cells transfected with cathepsin B-targeting and control siRNA for 48 h were treated with cisplatin. *p = 0.037. The results are presented as the mean ± SD of three independent experiments and analysed by Student's *t* test.Requirement of NLRP3 for cisplatin-induced IL-1β production. HK-1 cells transfected with NLRP3-targeting, AIM2-targeting, RIG-I-targeting and control siRNA for 48 h were treated with cisplatin. *p = 0.029. The results are presented as the mean ± SD of three independent experiments and analysed by Student's *t* test.Enhanced induction of tumour microenvironmental factor-stimulated IL-1β by therapeutic treatments. HK1 cells were treated with tumour microenvironmental factors, EBV gDNA/EBERs/ATP/H_2_O_2_ as previously described with or without therapies, irradiation and cisplatin. IL-1β production was used to measure inflammasome activity. *p = 0.00004, the results are presented as the mean ± SD of four independent experiments and analysed by Student's *t* test.

We next evaluated whether inflammasomes could be activated in NPC cells by the current treatment remedies, irradiation and cisplatin, which are known to induce ROS production (Kruidering et al, 1997; Valerie et al, 2007) and DNA damage (Siddik, 2003), resulting in cell death and the release of ATP (Martins et al, 2009). In addition, intracellular cisplatin is accumulated in lysosomes (Safaei et al, 2005), which are the reservoir of the NLRP3 inflammasome activator, cathepsin B (Schroder & Tschopp, 2010; Schroder et al, 2010). We found that IL-1β secretion was dose-dependently induced by irradiation (Fig 4A). Irradiation induced ROS production but not ATP release (Supporting Information Fig S7A and S7B), and the irradiation-induced IL-1β was specifically blocked by DPI (an NADPH oxidase inhibitor) and Z-VAD-FMK, but not by oATP (a P2X7-receptor antagonist) or apyrase (an ATP scavenger; Fig 4B). Notably, irradiation-induced IL-1β was reduced by depletion of AIM2, but not of NLRP3 or RIG-I (Fig 4C). To identify the direct, endogenous ligand of AIM2, we measured the levels of cytosolic DNA in NPC cells. As shown in Fig 4D, irradiation treatment resulted in elevated cytosolic levels of both nuclear (3.6-fold) and mitochondrial (4.1-fold) DNA. This level of induction was similar to results reported previously for the irradiation-mediated release of mitochondrial DNA into cytosol (Nakahira et al, 2011; Shimada et al, 2012). Conversely, we found that cisplatin treatment dose-dependently induced IL-1β secretion (Fig 4E) and ATP release but not ROS production (Supporting Information Fig S7C and S7D). Cisplatin-induced IL-1β was specifically blocked by CA-074-Me (cathepsin B inhibitor) and Z-VAD-FMK (Fig 4F), and was further demonstrated in cathepsin B-depleted cells treated with cisplatin (Fig 4G and Supporting Information Fig S4D). Interestingly, cisplatin-induced IL-1β was significantly reduced by depletion of NLRP3, but not of AIM2 or RIG-I (Fig 4H). Similar to HK1 cells, the inflammasome and IL-1β secretion were also activated by irradiation and cisplatin in NPC-TW02 cells (Supporting Information Fig S5C). The induction of AIM2 and NLRP3 inflammasome assembly in HK1 cells by irradiation and cisplatin, respectively, was confirmed by immunoprecipitation of endogenous ASC from irradiated and cisplatin-treated cell lysates with an ASC-specific antibody (Supporting Information Fig S8). Therefore, these results suggest that irradiation induces ROS-dependent AIM2 activation, while cisplatin induces cathepsin B-dependent NLRP3 activation (Schroder et al, 2010). Importantly, we demonstrate that therapeutic treatment activates the inflammasome in NPC cells and can enhance IL-1β production (from ∼174 to ∼492 pg/ml) in the presence of tumour microenvironmental factors (Fig 4I).

### Tumour-derived IL-1β inhibits tumour growth *in vivo*

**Figure 5:**
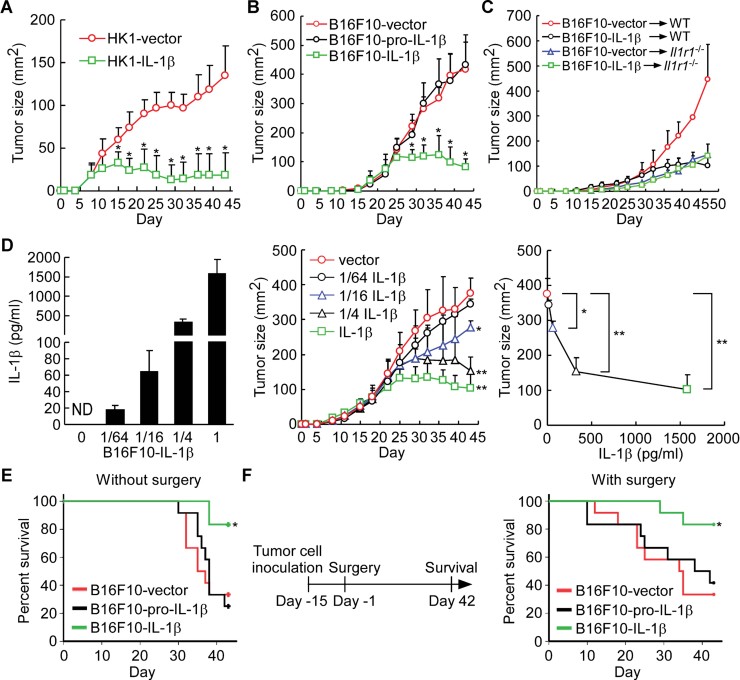
Inhibition of growth by tumour-derived IL-1β *in vivo* Tumour growth of IL-1β-expressing HK1 cells in a xenograft model. Nude mice were injected with HK1-vector or HK1-IL-1β cells (*n* = 9 per group). *p = 4.6E−04, 6.2E−06, 4.0E−06, 4.1E−07, 5.4E−09, 5.0E−08, 1.0E−06, 5.3E−07 and 4.3E−07 at day 15, 18, 22, 25, 29, 32, 36, 39 and 43 post-inoculation, respectively. All results are presented as the mean ± SD of three independent experiments and analysed by Student's *t* test.Tumour growth of IL-1β- and pro-IL-1β-expressing B16F10 cells in a syngeneic mouse model. Wild-type mice were injected with B16F10-vector, B16F10-pro-IL-1β and B16F10-IL-1β cells (*n* = 12 per group). *p = 1.1E−06, 1.5E−06, 6.9E−03, 9.7E−04 and 5.7E−03 at day 29, 32, 36, 39 and 43 post-inoculation, respectively. All results are presented as the mean ± SD of three independent experiments and analysed by Student's *t* test.Tumour growth of B16F10-vector and B16F10-IL-1β cells in wild-type and *Il1r1*^−/−^ mice (*n* = 4–6 per group). All results are presented as the mean ± SD of three independent experiments.Dose effect of IL-1β secretion on tumour growth inhibition. IL-1β in supernatants from B16F10-IL-1β cells premixed with B16F10-vector cells by indicated ratio (left panel). Tumour growth of B16F10-IL-1β mixed cells in a syngeneic mouse model (middle panel, *n* = 8 per group). Correlation between the tumour sizes at day 43 and IL-1β secretion of B16F10-IL-1β premixed cells (right panel). *p = 0.022 (1/16 IL-1β *vs.* vector); **p = 8.5E−04 (1/4 IL-1β *vs.* vector) and 1.2E−05 (IL-1β *vs.* vector). All results are presented as the mean ± SD of three independent experiments and analysed by Student's *t* test.Survival rate in mice bearing IL-1β- and pro-IL-1β-expressing B16F10 cells. Syngeneic mice were injected with B16F10-vector, B16F10-pro-IL-1β or B16F10-IL-1β cells (*n* = 12 per group) and survival curves were plotted using the Kaplan–Meier method and compared using the log-rank test. *p = 0.009. The results were obtained from three independent experiments.Survival rate in mice with surgical removal of primary tumours. B16F10-vector, B16F10-pro-IL-1β and B16F10-IL-1β tumours were established and then surgically removed after 14 days (*n* = 12 per group) and survival curves were plotted using the Kaplan–Meier method and compared using the log-rank test. *p = 0.036. The results were obtained from three independent experiments.

We examined the effect of tumour-derived IL-1β on tumour growth *in vivo* by using HK1-IL-1β and HK1-vector cells (Supporting Information Fig S9A and S9B). As shown in Fig 5A, HK1-derived IL-1β secretion significantly abolished tumour growth in nude mice *in vivo* starting at day 15, although tumour growth between HK1-vector and HK1-IL-1β cells was similar during the first week. The inhibitory effect of tumour-derived IL-1β on tumour growth was further evaluated in immunocompetent wild-type mice. B16F10 mouse melanoma cells, which do not express pro-IL-1β, NLRP3 and AIM2 (data not shown), were chosen to generate B16F10-IL-1β, B16F10-pro-IL-1β (expressing non-secretable IL-1β) and B16F10-vector cells (Supporting Information Fig S9C and S9D). B16F10 cell-derived IL-1β significantly abolished tumour growth in wild-type syngeneic C57BL/6 mice and nude mice at days 29 and 36, respectively, although the tumour growth was similar during the first 3 weeks for B16F10-IL-1β, B16F10-pro-IL-1β and B16F10-vector cells (Fig 5B and Supporting Information Fig S10).

Since IL-1β does not directly inhibit tumour cell proliferation (Supporting Information Fig S9B and S9D), the underlying mechanisms involved in the IL-1β-mediated inhibitory effect *in vivo* most likely occur through an indirect paracrine manner. Therefore, the role of the host's IL-1β receptor-mediated signalling in tumour growth was further studied in *Il1r1*^−/−^ mice. As shown in Fig 5C, IL-1β negative B16F10-vector tumours grew faster in wild-type mice than in *Il1r1*^−/−^ mice, suggesting that IL-1β produced by inflammatory cells and stromal cells may promote tumour growth. In addition, the tumour size of B16F10-IL-1β- and B16F10-vector tumours was similar in *Il1r1*^−/−^ mice, indicating that the inhibiting effect of tumour-derived IL-1β on tumour growth was dependent on the host response. Our data support the view that IL-1β has contradictory roles in protumour and antitumour activity, depending on the host immune response. Thus, in order to determine the amount of tumour-derived IL-1β required for inhibition of tumour growth, B16F10-IL-1β and B16F10-vector cells were mixed at different ratios (0:1, 1:64, 1:16, 1:4 and 1:0) to obtain low to high levels of secreted cytokine (0, 17.8, 64.2, 328.6 and 1580.3 pg/ml, respectively in Fig 5D, left panel). The cell mixtures were inoculated into wild-type mice, and tumour sizes were monitored for 43 days. The outgrowing tumours indeed contain a mixture of IL-1β expressing and non-expressing cells (Supporting Information Fig S11). As shown in Fig 5D, middle panel, tumour growth was inhibited by tumour-derived IL-1β in a dose-dependent manner. At day 43, mice carrying cells secreting relatively low levels of IL-1β (1/16 B16F10-IL-1β) harboured significantly smaller tumours (277 mm^2^) than those of B16F10-vector cells (376 mm^2^). Our data showed that the threshold for the antitumour activity of IL-1β was 64.2 pg/ml (Fig 5D, right panel).

The survival rates of mice carrying B16F10-IL-1β tumours (83.3%) were significantly better than for mice carrying tumours arising from B16F10-pro-IL-1β (25.0%, *p* = 0.003) or B16F10-vector (33.3%, *p* = 0.006) cells (Fig 5E). We further studied the effect of tumour-derived IL-1β on tumour recurrence following surgical removal of the large primary tumours. Mice with IL-1β-secreting B16F10-IL-1β tumours showed better survival (83.3%) than those with tumours arising from B16F10-pro-IL-1β (41.7%, *p* = 0.034) or B16F10-vector (33.3%, *p* = 0.01) cells (Fig 5F). Collectively, the data show that high levels of tumour-derived IL-1β can inhibit tumour growth and local recurrence. This is consistent with the clinical correlation between high levels of inflammasome protein and IL-1β expression and better survival in NPC patients after treatment (Fig 1D). Thus, our findings strongly suggest that tumour-derived IL-1β can contribute to local tumour control and better survival.

### Tumour-derived IL-1β can induce neutrophil infiltration into tumours

**Figure 6:**
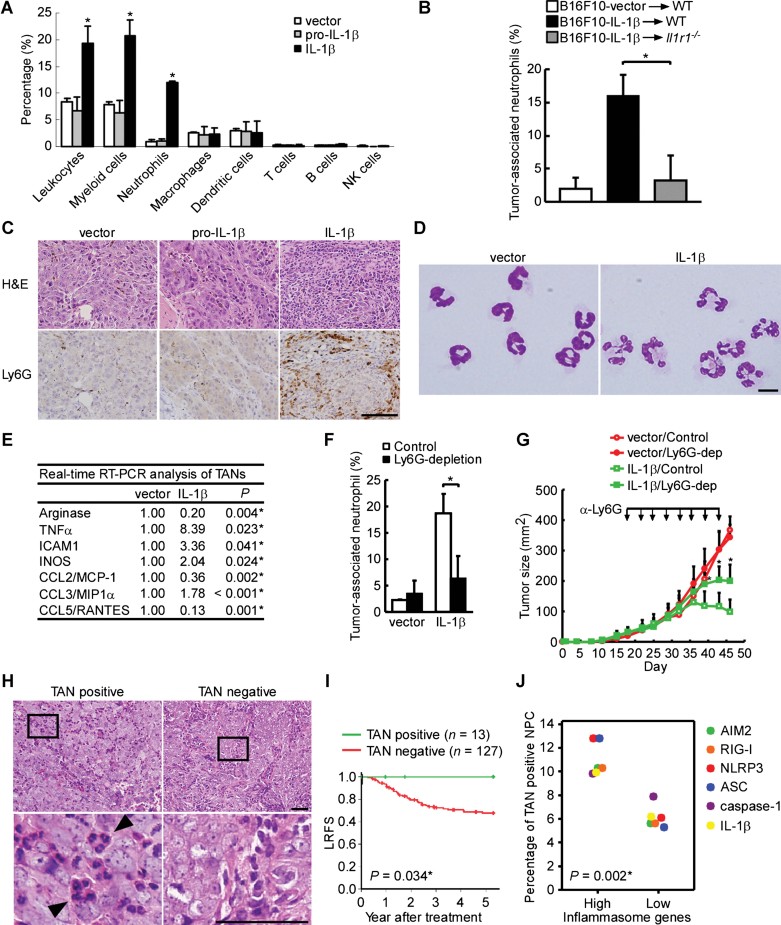
TANs as important effector cells for the IL-1β-mediated antitumour activity Leukocyte subsets in tumours. Percentage of leukocytes (CD45^+^), myeloid cells (CD11b^+^), neutrophils (CD11b^+^/Ly6G^+^), macrophages (CD11b^+^/F4/80^+^), dendritic cells (CD11c^+^), T cells (CD3^+^), B cells (B220^+^) and NK cells (NK1.1^+^) in the B16F10-IL-1β-, B16F10-pro-IL-1β- or B16F10-vector-bearing tumours (*n* = 3 per group) was determined by flow cytometry. *p = 0.0364, 0.0222 and 0.0004 for leukocytes, myeloid cells and neutrophils, respectively. The results are presented as the mean ± SD of three independent experiments and analysed by Student's *t* test.Induction of intratumoural neutrophils by IL-1β. Percentage of TANs (CD11b^+^/Ly6G^+^) in the B16F10-IL-1β and B16F10-vector-bearing tumours (*n* = 3 per group) in wild-type and *Il1r1*^−/−^ mice was determined by flow cytometry. *p = 0.003. The results are presented as the mean ± SD of three independent experiments and analysed by Student's *t* test.Increased TANs in B16F10-IL-1β tumour. Ly6G immunohistochemistry performed on tumour tissues. Scale bars, 100 µm.The morphology of TANs. Neutrophils were sorted from CD11b^+^/Ly6G^+^ cells in B16F10-vector or B16F10-IL-1β tumours. Scale bars, 10 µm.Quantitative RT-PCR analysis. The fold change of gene expression in purified neutrophils (CD11b^+^/Ly6G^+^) of B16F10-IL-1β- and B16F10-vector-tumours, using the expression level in TANs from B16F10-vector-tumours as the denominator, was calculated (*n* = 3 per group). The results were obtained from three independent experiments and analysed by Student's *t* test.Neutrophil depletion. Mice bearing B16F10-IL-1β- or B16F10-vector-tumours were injected with either the anti-Ly6G 1A8 or a control IgG antibody intraperitoneally twice a week (*n* = 4 per group). After 2 weeks, the percentages of TANs (CD11b^+^/Ly6G^+^) in whole tumour cells were determined by flow cytometry. *p = 0.005. The results are presented as the mean ± SD of four independent experiments and analysed by Student's *t* test.Effect of neutrophil depletion on tumour growth. Mice bearing B16F10-IL-1β- or B16F10-vector-tumours were injected with antibodies described in (**F**) intraperitoneally (arrowheads) twice a week during the study period (*n* = 7–9 per group). *p = 0.006, 0.003 and 0.002 at day 39, 43 and 46 post-inoculation, respectively. All results are presented as the mean ± SD and analysed by Student's *t* test. Each experiment was repeated at least twice.TANs in NPC tumours. The H&E results are shown at 400× magnification (upper panel) and the enlarged box areas (lower panel). Arrowhead indicates the TANs. Scale bars, 50 µm.Kaplan–Meier survival analysis of LRFS.Correlation of TANs and pro-IL-1β and inflammasome components in NPC biopsies. The positive rates of TANs in patients with high expression levels of AIM2, RIG-I, NLRP3, ASC, caspase-1 and IL-1β *versus* the positive rates of TANs in patients with low expression levels of the above-described proteins are analysed using Student's *t* test (Supporting Information Table S13).

Since the inhibitory effect of IL-1β on tumour growth was dependent on IL-1β receptor-mediated signalling of the host immune system (Fig 5B and C), we further investigated the composition of tumour-infiltrated leukocyte subsets from tumours by flow cytometry. We found that the infiltration of leukocytes, myeloid cells and neutrophils was dramatically higher in B16F10-IL-1β tumours, compared with B16F10-pro-IL-1β and B16F10-vector tumours (Fig 6A). In contrast, the proportion of macrophages, dendritic cells, T cells, B cells and NK cells was not affected by the IL-1β-secreting tumours. However, the effect of tumour-derived IL-1β on neutrophil infiltration was completely abolished in *Il1r1*^−/−^ mice (Fig 6B). This suggested that the tumour-derived IL-1β induced neutrophil infiltration through the activation of IL-1β receptor signalling in host cells. Further confirmation of neutrophil infiltration into B16F10-IL-1β tumours was provided by immunohistochemical staining of tumour tissues using a specific antibody against neutrophil marker Ly6G. As shown in Fig 6C, largely infiltrating neutrophils were observed in B16F10-IL-1β tumours, but not in B16F10-pro-IL-1β and B16F10-vector tumours. These intratumoural CD11b^+^/Ly6G^+^ cells (tumour-associated neutrophils, TANs) indeed had a clear neutrophil-like morphology (Fig 6D). Interestingly, most of the TANs in B16F10-IL-1β tumours were more lobulated and hypersegmented; in contrast, those in B16F10-vector tumours maintained the characteristic banded appearance typical of blood neutrophils. Our data indicate that neutrophils are the mainly infiltrated cell type recruited by tumour-derived IL-1β.

### The TANs present in IL-1β-expressing tumours have an immunostimulatory phenotype

Since TANs are composed of two subtype cells, antitumour N1 TANs (immunostimulator) and protumour N2 TANs (immunosuppressor) (Fridlender et al, 2009; Hofman, 2010; Mantovani, 2009), we compared the mRNA levels of selected cytokines, adhesion molecules and chemokines in TANs from B16F10-IL-1β tumours *versus* B16F10-vector tumours by quantitative RT-PCR (Fig 6E). The levels of arginase mRNA, an important immunosuppressor of the adaptive immune system (Rodriguez & Ochoa, 2008), were 5.0-fold lower in TANs from B16F10-IL-1β tumours. In contrast, the mRNA levels of the important immunostimulators, TNF-α, ICAM1 and iNOS were significantly increased. The levels of neutrophil chemoattractants, CCL2 and CCL5 were significantly reduced, whereas CCL3 partially increased. Our data show that tumour-derived IL-1β can polarize TANs towards an N1 TAN-like immunostimulatory phenotype.

### TANs are important effector cells for IL-1β-mediated antitumour activity

We next studied the functional significance of TANs in IL-1β-mediated tumour control by depleting the Ly6G^+^ cells in tumour-bearing animals. Tumour cells were inoculated in the footpad, followed by intraperitoneal injection of an anti-Ly6G monoclonal antibody 1A8 or isotype-matched control IgG intraperitoneally, and tumour growth was then determined. A significant reduction (66%) in TANs from B16F10-IL-1β tumour-bearing mice was noted 2 weeks after treatment (Fig 6F). Palpable tumours were observed at day 18, and antibodies were introduced as described above twice per week until day 43. As shown in Fig 6G, the tumour size of B16F10-IL-1β tumour-bearing mice with neutrophil depletion increased significantly at day 39 (*p* = 0.006) as compared to B16F10-IL-1β tumour-bearing mice treated with isotope antibody. In contrast, depletion of neutrophils in B16F10-vector tumour-bearing mice did not affect tumour growth. Together, these data indicate that depletion of TANs resulted in significant abrogation of the IL-1β-mediated antitumour activity, suggesting that TANs contribute to the antitumour activity of tumour-derived IL-1β.

### Association of TANs with better survival in NPC patients

We further analyzed the significance of TANs on tumour development in NPC patients. Thirteen out of the 140 tested NPC specimens were TAN-positive as shown by hematoxylin and eosin (H&E) staining (Fig 6H). Kaplan–Meier survival analysis of the H&E results showed that the presence of TANs correlated with better LRFS (Fig 6I). Notably, none of the patients whose tumours were TAN-positive had local recurrence within 5 years after treatment (Fig 6I). In addition, the correlation of the protein levels of IL-1β and different inflammasome components with the positive rate of TANs was analysed using the NPC samples studied in Figs 1A and 6H. As shown in Fig 6J, the positive rates of TANs were higher in the patients with high level of AIM2, RIG-I, NLRP3, ASC, caspase-1 or IL-1β. Inflammasome genes were significantly correlated with neutrophil infiltration in NPC patients (Supporting Information Table S13). Taken together, our data in mice and humans are consistent with TANs playing a major role in local tumour control.

### Hierarchical cluster analysis of AIM2, RIG-I and NLRP3 inflammsome gene expression levels in 114 cancer cell lines

In order to delineate the function of inflammasomes in other cancer types, we evaluated the expression patterns of AIM2, RIG-I, NLRP3, ASC and caspase-1 in 114 cancer cell lines involving 22 cancer types. For this analysis, we used 324 Affymetrix U133 Plus 2.0 array data sets from the eight NPC cell lines generated in this study and 106 cell lines from the public GSK Cancer Cell Line Genomic Profiling Data (Greshock et al, 2010), according to a distance tree obtained through hierarchical cluster analysis. The cancer cell lines were separated into four clusters (Supporting Information Fig S12A and Table S14), and all eight NPC cell lines were clustered together and were closely grouped with 9 of 17 lymphoma and 2 of 8 brain cancer cell lines (designated as cluster 1) but not with cell lines of other cancer types (Supporting Information Fig S12B and S12C). Since both lymphoma and NPC are associated with EBV infection, we found that high levels of AIM2, RIG-I and NLRP3 inflammasome expression are significantly correlated with EBV infection in lymphoma cell lines (*p* = 0.043; Supporting Information Fig S12D), suggesting that EBV infection is a potential factor for AIM2, RIG-I and NLRP3 inflammasome overexpression in cancer cells of both epithelial and lymphocyte origin.

## DISCUSSION

**Figure 7:**
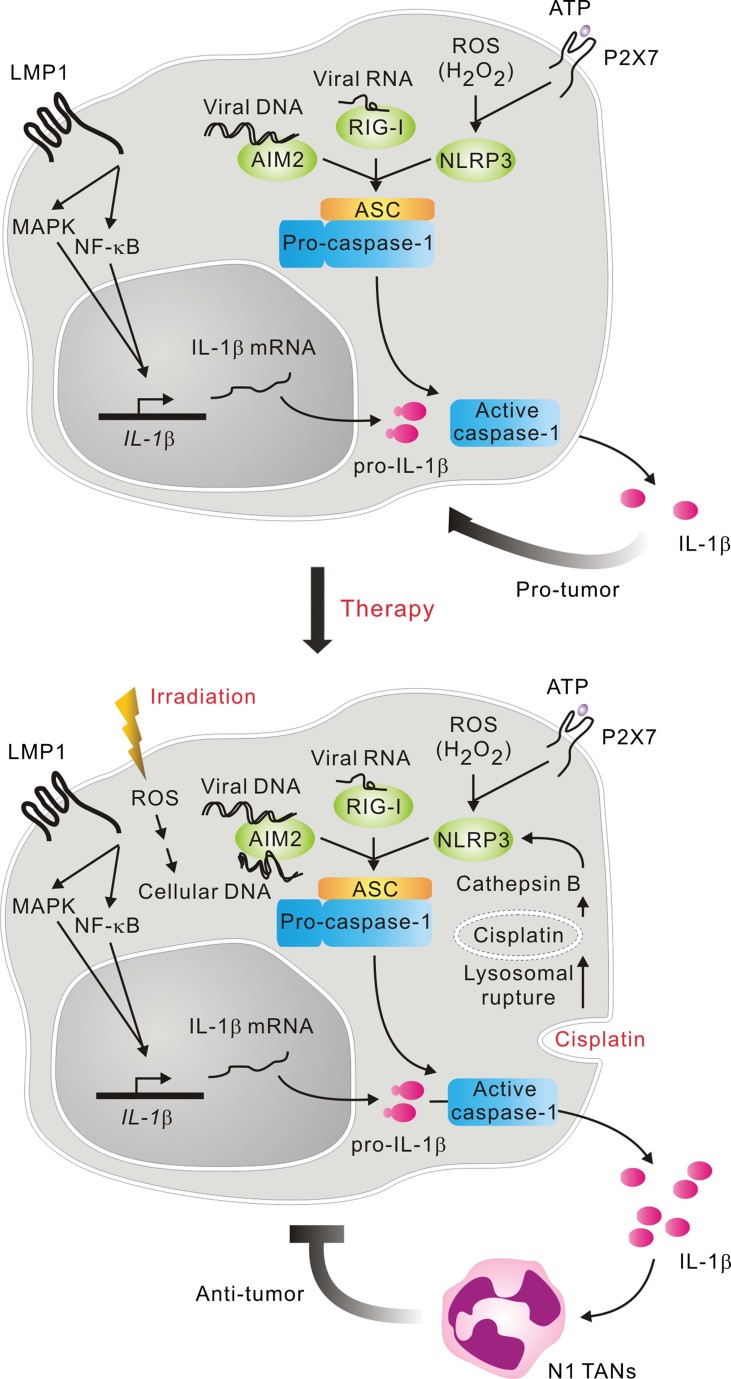
Role of inflammasomes in tumour cell responses to microenvironmental factors and therapy Before treatment, tumour cells are latently infected with EBV and express LMP1, which can induce pro-IL-1β production through NF-κB and MAPK signalling. In the tumour microenvironment, tumour cells encounter PAMPs (EBV gDNA and EBV-encoded RNAs, and EBER1 and EBER2) and DAMPs (intracellular ROS or H_2_O_2_, and extracellular ATP) produced by dying or stressed cells. The PAMPs and DAMPs can stimulate AIM2, RIG-I and NLRP3, which then form inflammasomes with ASC and caspase-1, resulting in cleavage of pro-IL-1β and low-level basal IL-1β secretion. Low levels of tumour-derived IL-1β can facilitate tumour growth. Treatment of NPC patients with irradiation leads to ROS production that leads to the release of nuclear and mitochondrial DNA into the cytosol of tumour cells, while chemotherapy (cisplatin) causes cathepsin B to be released from ruptured lysosomes due to cisplatin accumulation. Thus, AIM2 and NLRP3 in tumour cells are further activated to secret IL-1β at higher levels. Finally, high levels of tumour-derived IL-1β achieved by therapeutic treatment can inhibit tumour growth and local relapse by recruiting neutrophils, especially immunostimulatory N1 TANs.

In this report, we demonstrated a link between neutrophils and tumour suppression through inflammasome activation. In NPC, NLRP3, AIM2 and RIG-I inflammasomes were overexpressed in the tumour cells and were required for IL-1β production in response to PAMPs and DAMPs in the tumour microenvironment and therapeutic treatment. In addition, high levels of tumour-derived IL-1β can recruit a large number of TANs, which significantly inhibit tumour growth in mice. Importantly, the expression of NLRP3, AIM2 and RIG-I inflammasomes and IL-1β, as well as the presence of TANs, were associated with better survival in NPC patients. This is the first study to demonstrate that inflammasomes in the tumour cells can respond to PAMPs, DAMPs and therapeutic treatment, and contribute to TANs-dependent tumour suppression (Fig 7).

Inflammation has contradictory roles in carcinogenesis through production of tumour-promoting proinflammatory cytokines and facilitation of antitumour immune responses (Hagemann et al, 2007; Zitvogel et al, 2012). In viral infection-associated cancers, *e.g.* EBV-associated NPC, both PAMPs and DAMPs exist in the tumour microenvironment, where they are potential sources of stimuli for inflammasome activation *in vivo*. In addition, the tumour cells express viral antigens that can provide specificity for cytotoxic T cells (Berger et al, 2009; Grivennikov et al, 2010). Thus, the activation of tumour inflammasomes may facilitate antitumour immunity by initiating inflammation. Our findings show that the expression of inflammasomes and the proinflammatory cytokine, IL-1β, in tumour cells is significantly correlated with better survival in NPC patients (Fig 1), suggesting a role of tumour inflammasomes in tumour suppression. Although IL-1β has a protumourigenic effect [Fig 5C in this study; and (Apte & Voronov, 2008)], inflammasome and IL-1β-mediated antitumour activity have been reported recently by two other studies in gene-deficient mice, which described the effect of the NLRP3 and IL-1β produced by hematopoietic cells against cancer (Allen et al, 2010; Ghiringhelli et al, 2009). Consistent with their findings, our data show that the inflammasomes in malignant cells are responsive to PAMPs, DAMPs and therapeutic treatment (Figs 3 and 4), and the interaction of tumour-derived IL-1β (over 64.2 pg/ml) and its receptors on the host cells can inhibit tumour growth and recurrence (Fig 5). In addition, tumour growth and recurrence are inhibited by IL-1β, rather than pro-IL-1β (Fig 5B), suggesting that cleavage of pro-IL-1β to IL-1β by the inflammasome is critical for tumour suppression. Taken together, our work reveals that tumour cells are as able as immune cells to use the inflammasome for responding to infection, stress and therapeutic treatment. Paradoxically, when tumour-derived IL-1β reaches a certain threshold, it can also limit tumour growth *in vivo*.

Our findings are in line with the emerging concept of tumour inflammation, according to which cancer cells are able to manipulate the host response by recruiting immune cells (Demaria et al, 2010; Grivennikov et al, 2010; Hagemann et al, 2007). We demonstrate that tumour-derived IL-1β can alarm the immune system to induce an influx of neutrophils to tumour sites (Fig 6A and B). These findings are consistent with recent reports that NLRP3 activation and IL-1β in experimental liver injury can direct neutrophil trafficking by drawing neutrophils out of the circulation to adhere to the vascular endothelium at inflammation sites (McDonald et al, 2010). More significantly, depletion of these neutrophils significantly blunts the antitumour effect of IL-1β (Fig 6G), showing that IL-1β-induced TANs act as the effectors of tumour elimination. These IL-1β-induced TANs express more immunoactivating cytokines and less immune-suppressing arginase (Fig 6E). Thus, besides the recent reports showing the tumour cytotoxicity of N1 TANs through TGF-β blockade (Fridlender et al, 2009) and tumour-entrained neutrophils by CCL2 (Granot et al, 2011), we identify a new mechanism mediated by IL-1β. Furthermore, the presence of TANs is associated with better survival in NPC patients (Fig 6I) and advanced gastric carcinoma (Caruso et al, 2002). The partial effect of granulocyte depletion on recovering tumour growth may be attributed to the activity of remaining immunostimulatory TANs. In addition, tumour-derived IL-1β may potentially induce dendritic cell-dependent adaptive immunity against tumours (Ghiringhelli et al, 2009). Taken together, our findings support the hypothesis that TANs are recruited by tumour-produced cytokines, *e.g.* IL-1β, and exert a host-mediated cytotoxic effect against the tumour.

A correlation between an elevated neutrophil to lymphocyte ratio (NLR) in the blood stream and poor survival was recently reported in NPC (An et al, 2011). While the underlying mechanisms remain elusive, it may be attributed to the following reasons. First, elevated NLR-associated lymphocytopenia may reduce lymphocyte-dependent cytotoxic cell death, which is important for antitumour activity. Second, elevated NLR-associated neutrophilic leukocytosis may increase the production of angiogenic growth factors that function in tumour-related angiogenesis and metastasis (An et al, 2011). Improving the current treatment for patients with elevated NLR is important. Our and Fridlender et al's findings on the recruitment and activation of TANs by IL-1β and TGF-β blockade shed some light on the possibility of conversion of circulating protumour neutrophils to intratumoural antitumour neutrophils (Fridlender et al, 2009). One might consider taking advantage of the antitumour activity of N1 TANs to improve the treatment of the patients with elevated NLR-associated neutrophilic leukocytosis.

Irradiation and cisplatin are widely used for cancer treatment. For the first time, we showed that irradiation can activate the cytosolic DNA receptor, AIM2, via ROS production in malignant cells, correlating with the release of nuclear and mitochondrial DNA into the cytosol [Fig 4D and (Patrushev et al, 2006)]. Similar to a recent report indicating that the release of mitochondrial DNA during ATP-triggered apoptosis can activate NLRP3 inflammasome in macrophages (Nakahira et al, 2011; Shimada et al, 2012), we found that inflammasomes activated by ROS production and cytosolic DNA release during apoptosis can facilitate antitumour immunity through IL-1β-mediated inflammation. We also showed that cisplatin can induce IL-1β secretion in NPC cells, which is in agreement with a previous study indicating that cisplatin can activate caspase-1 (Kondo et al, 1995) and accumulates in lysosomes (Safaei et al, 2005), which are the reservoir of cathepsin B (Schroder & Tschopp, 2010; Schroder et al, 2010). Here, we link cisplatin and inflammasomes and show that the cathepsin B-dependent NLRP3 inflammasome can respond to cisplatin and further induce caspase-1 activation and IL-1β production (Fig 4E–H). Furthermore, cathepsin B is highly expressed in NPC (Chang et al, 2010). Thus, our results suggest that therapeutic treatment can cooperate with PAMPs and DAMPs in a tumour microenvironment to induce more IL-1β secretion, which consequently results in efficient tumour suppression by recruiting neutrophils, especially immunostimulatory N1 TANs. The association between therapeutic induction of IL-1β and neutrophil recruitment cannot be demonstrated because the collection of primary tumour biopsies from patients who had recently undergone treatment is not ethical and would not be allowed. Nonetheless, we provide strong evidence that the chemoradiotherapeutic activation of the NLRP3 and AIM2 inflammasomes, via cathepsin B and ROS, is likely to emerge as a crucial player in the regulation of cancer immunotherapy. Compared with the results of clinical trials showing that IL-1β treatment through intravenous infusion and subcutaneous administration effectively increases the level of peripheral neutrophils in cancer patients (Veltri & Smith, 1996), our findings show that manipulating tumour-derived IL-1β by chemoradiotherapy to induce N1 TANs should be considered as a strategy for improving immunotherapy, and that inflammasomes are promising therapeutic targets in cancers.

Our results showed that pro-IL-1β is induced by LMP1 in 4 NPC cell lines, NPC-TW01, -TW02, -TW04 and HK1; similar induction was previously reported in NPC-TW01 (Huang et al, 2010). Since inflammasome-mediated release of IL-1β can drive chronic inflammation with protumour activity (Fig 5C; Apte & Voronov, 2008), this might explain why in EBV-associated cancers, the inflammasome genes are induced and activated by viral factors such as LMP1 and EBERs (Fig 7). The practical implications of this study are the discovery of new roles for inflammasomes, which are favourable prognostic biomarkers for cancer patients, and the identification of inflammasomes are promising therapeutic targets in cancers.

## MATERIALS AND METHODS

### Patients and clinical characteristics

The retrospective cohort comprised 144 NPC patients who had been admitted to Chang Gung Memorial Hospital (CGMH) at Lin-Kou from 1990 to 2000. Clinical stage was defined according to the 2002 cancer staging system revised by the American Joint Committee on Cancer. Histological typing was done according to the World Health Organization (WHO) classification criteria (Chen et al, 2008). This study was reviewed and approved by the institutional review board and ethics committee of CGMH. Informed consent was obtained from all subjects and the experiments conformed to the principles set out in the WMA Declaration of Helsinki (http://www.wma.net/en/30publications/10policies/b3/) and the NIH Belmont Report (http://ohsr.od.nih.gov/guidelines/belmont.html). Patients diagnosed with distant metastatic disease at presentation (M1 stage) and/or those who had undergone previous treatment at another institute were excluded from the present study. Patient characteristics and clinical features are summarized in Supporting Information Tables S15–S17. The primary end-point was LRFS and DFS, which was calculated from the date of diagnosis to the date of death or the last follow-up.

### Immunohistochemical staining

Immunohistochemical analyses were described previously (Chen et al, 2008, 2010b, 2011). The antibodies for individual proteins can be found in Supporting Information Table S18. Protein expression was assessed by quantitative scoring of the staining intensity and the proportion of positively stained cells. The staining intensity was graded as 0, 1, 2 or 3 to indicate undetectable, weak, moderate and strong staining, respectively. These scores were multiplied by the percentage of cells that showed positive staining; the resulting scores, which were taken as reflecting protein expression, were used to classify the specimens/patients into two groups: ‘high-level’ expression (ASC scores > 120, caspase-1 scores > 100, IL-1β scores > 40, NLRP3 scores > 160, AIM2 scores > 140, RIG-I scores > 10, CIITA scores > 50, NLRC4 scores > 100, NLRP7 scores > 60) and ‘low-level’ expression (ASC scores ≤ 120, caspase-1 scores ≤ 100, IL-1β scores ≤ 40, NLRP3 scores ≤ 160, AIM2 scores ≤ 140, RIG-I scores ≤ 10, CIITA scores ≤ 50, NLRC4 scores ≤ 100, NLRP7 scores ≤ 60). ASC-, caspase-1-, IL-1β-, NLRP3-, AIM2-, RIG-I-, CIITA-, NLRC4- and NLRP7-positive tumour cells in representative microscopic fields were scored independently by two experienced pathologists.

### Definition of TANs in NPC patients

Hematoxylin and eosin-stained sections were examined to identify TANs (Reid et al, 2011). Only foci with neutrophils within and/or immediately adjacent to tumour cells were taken into consideration. The areas immediately adjacent to necrotic tissue were disregarded. Ten non-overlapping fields were examined. The number of TANs was assessed and the areas with ≤10 neutrophils/100 epithelial cells were considered negative and areas with >10 neutrophils/100 epithelial cells were considered as positive for TANs. The cut-off value, >10 neutrophils/100 epithelial cells, was defined as TANs positive, which was determined from Receiver Operating Characteristics curve analysis. The criteria have also been used in a recent publication by Reid MD et al (Reid et al, 2011).

### Reagents

ATP, poly (dA:dT), oATP, apyrase, DPI, CA-074 Me, Z-VAD-FMK and cisplatin were purchased from Sigma–Aldrich and H_2_O_2_, BAY11-7082, SP600125, SB203580 and PD98059 from Calbiochem.

### Cell culture and treatments

NPC-TW01, -TW02, -TW04 and HK1 cells were cultured under conditions described before (Chen et al, 2010b, 2011). THP-1, CNE-1, HONE-1 and HK1-EBV cells were grown in RPMI-1640 medium and BM1 cells were grown in Dulbecco's modified Eagle's medium (DMEM), supplemented with 10% fetal bovine serum, 100 U/ml penicillin and 100 µg/ml streptomycin at 37°C under 5% CO_2_. HK1-EBV cells were maintained with 500 µg/ml G418. Lentiviruses were established by following the protocol of the RNAi Core, Taiwan (http://rnail.genmed.sinica.edu.tw), and used to generate human IL-1β-expressing or vector-control HK1 cells, as well as mouse pro-IL-1β-expressing, mouse IL-1β-expressing or vector-control B16F10 cells. For transfection assays, sonicated EBV gDNA (around 500 bp), poly (dA:dT), pEGFP, EBER1/2, pHEBo, pHEBo-EBER1/2 were transfected by Lipofectamine 2000 (Invitrogen). For irradiation treatment, HK1 cells were irradiated at 10, 20, 30 or 40 Gy using Gammacell 3000 Elan (MDS Nordion) at 5.0 Gy/min.

### Animal tumour models

Mouse experiments were performed under the approval of the Institutional Animal Care and User Committee of Chang-Gung University. C57BL/6 and nude mice (6-week-old male) were obtained from National Laboratory Animal Center, Taiwan. *Il1r1*^−/−^ mice in the C57BL/6 background were purchased from The Jackson Laboratory, and bred in our animal facility under SPF conditions. The tumours were established at the intra-foot pad (i.f.p.) with 1 × 10^5^ tumour cells. Both survival and local tumour growth were determined twice weekly. For the surgery treatment, the tumours were established with 1 × 10^6^ cells by i.f.p. injection and the tumour-bearing leg was amputated below the knee at day 14. For FACS, RNA and cell subset isolation studies, tumours were harvested from the mice, and digested with 60 µg/ml DNase I (Sigma–Aldrich) and 1 mg/ml collagenase D (Roche) at 37°C for 20 min. All experiments were repeated at least twice.

### Flow cytometric analysis of tumour-infiltrating cells and isolation of CD11b^+^/Ly6G^+^ neutrophils

Tumour cells were analysed by FACSCalibur flow cytometer and CellQuest pro software (BD Biosciences). The CD11b^+^/Ly6G^+^ neutrophils were sorted using a FACSAria cell sorter (BD Biosciences). Fluorescently labelled antibodies were listed in Supporting Information Table S19.

### Evaluation of the morphology of tumour neutrophils

The sorted CD11b^+^/Ly6G^+^ cells from tumours were collected using a Shandon Cytospin 4 (Shandon Southern Instrument, Inc.) at 800 rpm for 5 min and stained with Liu's stain solution (Muto Pure Chemicals).

**The paper explained**

PROBLEM:

Inflammasomes sense infection and cellular damage, and are critical for triggering inflammation through IL-1β production. To date, there has been no study on the roles of inflammasomes and their clinical implications in virus-associated human cancers. Inflammasomes are activated by two major groups of activators, pathogen-associated molecular patterns (PAMPs) and damage-associated molecular patterns (DAMPs), which are potentially present in the tumour microenvironment of infection-associated cancers. However, the expression profile of inflammasomes in malignant cells remains unclear. NPC is closely associated with EBV infection. The current treatment remedies are irradiation and cisplatin; however, local recurrence after the treatment remains a major clinical problem. In addition, the link between inflammasome function in NPC cells, on the one hand, and EBV infection- and stress-induced inflammation and cancer, on the other, is unknown.

RESULTS:

We addressed the problem by carrying out comprehensive analyses of inflammasomes using clinical samples and mechanistic studies *in vitro* and *in vivo*. AIM2, RIG-I and NLRP3 inflammasomes in NPC tumour cells, as well as tumour-associated neutrophils were identified as new markers for favourable prognosis in NPC. Inflammasomes in tumour cells can be activated by tumour microenvironmental factors including microbial products (EBV genomic DNA and EBV-encoded RNAs, EBERs) and tumour-derived danger signals (ATP and H_2_O_2_). Importantly, the therapeutic treatments, irradiation and cisplatin, can activate AIM2 and NLRP3 inflammasomes, respectively, and further enhance IL-1β production in the presence of tumour microenvironmental factors. Tumour-derived IL-1β can mediate anti-tumour activity by recruiting immunostimulatory neutrophils, which function as the effector cells for the inhibitory effect of tumour-derived IL-1β on tumour growth. In addition, high expression of AIM2, RIG-I and NLRP3 inflammasomes is significantly correlated in EBV-positive lymphoma cell lines, suggesting that EBV infection is a potential factor for inflammasome overexpression in cancer cells.

IMPACT:

This is the first study to establish the roles of inflammasomes in tumour cells responding to tumour microenvironmental factors and therapeutic treatments, and contributing to tumour-associated neutrophil-dependent tumour suppression. The elevated expression of NLRP3, AIM2 and RIG-I inflammasomes and IL-1β in malignant cells and tumour-associated neutrophils can be useful as prognostic biomarkers in NPC patients and may be important targets for developing treatments in EBV-associated cancers.

### *In vivo* depletion of Ly6G^+^ neutrophils

Neutrophils were depleted by using 100 µg of purified monoclonal anti-Ly6G antibody 1A8 or isotype-matched control IgG (BD Biosciences) via intraperitoneal injections (i.p.) starting 18 days after inoculation with tumour cells, and followed by i.p. injection twice a week throughout the entire experimental period. Tumour neutrophil depletion was confirmed by flow cytometry.

### Immunoprecipitation and Western blotting

Immunoprecipitation and Western blotting have been previously described (Chen et al, 2009). The detailed information of antibodies is shown in Supporting Information Table S20.

### Quantitative PCR

Quantitative RT-PCR analysis has been previously described (Chen et al, 2009). Primers are presented in Supporting Information Table S21. The gene expression results of human and mouse specimens were normalized to the expression of *GADPH* and *MRPL32*, respectively. For measurement of cytosolic DNA, we used the method modified by Nakahira K. et al. (Nakahira et al, 2011). 5 × 10^6^ cells were homogenized with a Dounce homogenizer in 300 µl of 100 mM Tricine–NaOH solution, pH 7.4, containing 0.25 M sucrose, 1 mM EDTA and protease inhibitor, then were centrifuged at 700*g* for 10 min at 4°C. 250 µl of supernatant were taken and further centrifuged at 10,000*g* for 30 min at 4°C. Two hundred microlitres of supernatant were taken as the cytosolic fraction. The amount of nuclear DNA encoding 18S ribosomal RNA and mitochondrial DNA encoding cytochrome *c* oxidase 1 were measured by quantitative RT-PCR with same volume of the cytosolic fraction.

### *In vitro* synthesis of EBER1 and EBER2

EBER1 and EBER2 cDNAs generated from C666-1 NPC cells by reverse transcription were cloned into pGEMTeasy (Promega). Purified PCR products of T7 promoter-fused EBER1 or EBER2 were used as a template of *in vitro* transcription (Epicenter). The primers used for cloning are listed in Supporting Information Table S21.

### RNA interference

RNA interference has been previously described (Chen et al, 2010b). The oligonucleotide sequences of dsRNA duplexes are presented in Supporting Information Table S22.

### IL-1β ELISA

Cell culture supernatants were assayed for human IL-1β (eBioscience) and mouse IL-1β (R&D Systems).

### Cell proliferation assay

5 × 10^4^ cells were seeded, and total cell numbers were counted each day for 4 days.

### Transfection of LMP1-expressing plasmids

The LMP1 constructs and transfection methods have been described previously (Chen et al, 2010a).

### Statistical analysis

Statistical analyses were performed using the SPSS 13.0 statistical software package. Clinicopathologic features were evaluated using the Pearson Chi-Square test. Survival curves were plotted using the Kaplan–Meier method and compared using the log-rank test. The Cox proportional hazards model was applied for multivariate analysis. The cut-off values to define high intensity of IHC staining in tumour cells were determined from Receiver Operating Characteristics curve analysis. *In vitro* data and tumour growth in mouse were analysed with the two-tailed Student's *t* test. Differences were considered significant at *p* < 0.05. The error bars were calculated and represented in terms of mean ± SD.

## Supplementary material

Detailed facts of importance to specialist readers are published as ”Supporting Information”. Such documents are peer-reviewed, but not copy-edited or typeset. They are made available as submitted by the authors.
