# Supplementary material for: Tumour inflammasome-derived IL-1β recruits neutrophils and improves local recurrence-free survival in EBV-induced nasopharyngeal carcinoma
Source: EMBO Mol Med. 2012 Oct 15;4(12):1276–93. doi: 10.1002/emmm.201201569 (PMC3531603; doi:10.1002/emmm.201201569)
Supplement: Supplementary file 1 [file emmm0004-1276-SD1.pdf]

Manuscript EMM-2012-01569

Tumor inflammasome-derived IL-1β recruits neutrophils and improves local recurrence-free survival in EBV-induced nasopharyngeal carcinoma

Lih-Chyang Chen, Li-Jie Wang, Nang-Ming Tsang, David M. Ojcius, Chia-Chun Chen, Chun-Nan OuYang, Chuen Hsueh, Ying Liang, Kai-Ping Chang, Chiu-Chin Chen, and Yu-Sun Chang

Corresponding author: Yu-sun Chang, Chang Gung University

Review timeline:

Submission date:	14 May 2012
Editorial Decision:	12 June 2012
Revision received:	22 August 2012
Editorial Decision:	31 August 2012
Revision received:	07 September 2012
Accepted:	13 September 2012

Transaction Report:

(Note: With the exception of the correction of typographical or spelling errors that could be a source of ambiguity, letters and reports are not edited. The original formatting of letters and referee reports may not be reflected in this compilation.)

1st Editorial Decision 12 June 2012

Thank you for the submission of your research manuscript to EMBO Molecular Medicine. We have now heard back from the three referees whom we asked to evaluate your manuscript. As you will see from the reports below, the referees find the topic of your study of potential interest. However, they raise substantial concerns on your work, which should be convincingly addressed in a major revision of the present manuscript.

Although the referees find the study to be of potential interest, they also raise a number of concerns about the technical quality of some of the data that should be improved (lack of controls, missing detailed descriptions...). In addition referee #2 would really want to see data from a second cell line to strengthen the results and consequently, conclusions of the study. Referee #3 is confused about the rationale behind IL-1b promoting tumor development as this is not properly addressed in the mouse model.

Given these evaluations, I would like to give you the opportunity to revise your manuscript, with the understanding that the referees' concerns must be fully addressed and that acceptance of the manuscript would entail a second round of review. Please note that that it is our journal's policy to allow only a single round of revision, and that acceptance or rejection of the manuscript will therefore depend on the completeness of your responses and the satisfaction of the referees with it.

Revised manuscripts should be submitted within three months of a request for revision; they will otherwise be treated as new submissions, except under exceptional circumstances in which a short extension is obtained from the editor. Also, the length of the revised manuscript may not exceed

60,000 characters (including spaces).

I look forward to seeing a revised form of your manuscript as soon as possible.

Yours sincerely,

Editor
EMBO Molecular Medicine

***** Reviewer's comments *****

Referee #1:

Chen et al. Show in their paper that EBV-associated nasopharyngeal carcinoma (NPC) is assocated with neutrophil recruitment, where it constitutes a positive prognostic factor. Moreover, inflammasome components (NLRP3, AIM2, RIG-I, ASC, caspase-1) and their product IL-1beta are found to be expressed in NPC specimens, and high mRNA expression levels were associated with better survival in NPC patients. The mechanisms leading to inflammasome activation by EBV have been studied. In an animal model, IL-1beta is suggested to be required for the infiltration of neutrophils, which would mediated antitumor effects. Altogether, the paper contains several convicing datasets that plead in favor of the implication of EBV-induced IL-1beta production in the attraction of neutrophils into the tumor bed.

The following relatively minor problems arise:

- The title of the paper should mention that the tumors are EBV-induced.
- A table summarizing the patient characteristics, as well as the measurement that have been done, should be provided for the supplemental material.
- All immunoblots lack size markers.
- The effect of granulocyte depletion demonstrated in Fig. 6G is partial. The authors should comment on this result.
- The boxes in Fig. 6H do not correspond to the enlarged panels.
- The authors should attempt a correlation between the density of granulocytes infiltrats in patients and their impact on survival. As it stands, it is not clear how they chose the threshold to separate positive and negative patients.
- Do the mRNA levels for IL-1beta and different inflammasome compoents measured in patient samples correlate with the density of the neutrophil infiltrate? This is an important question since it allows to validate or invalidate the working hypothesis proposed by the authors.

Referee #2:

This study suggests that inflammasome-triggered IL-1beta production in nasopharyngeal carcinoma (NPC) leads to neutrophil recruitment and favorable prognosis. The key supporting data are summarized in Figure 1 and Figure 6I. Much of the intervening basic biology data are uncontroversial and consistent with a large body of published work.

The study was built on the initial observation that 8/33 inflammosome-related genes were overexpressed in NPC tumors relative to adjacent normal tissues. This is, however, misleading and contradictory, as the data in Table 1 show that only 4 (CIITA, AIM2, ASC and IL-1beta) were significant. The rationale for the inclusion of RIG-1 and NLRP3 in the subsequent experiments is therefore obscure.

I also have 2 major concerns regarding the data in Fig 1. First, the definitions for 'Low' and 'High' expression of the inflammasome proteins appear arbitrary and different for each antibody. Secondly, no statistician has been identified in the author contributions; I would therefore be more comfortable

if the methodology and the analyses were independently reviewed by an expert statistician.

Regarding Figure 1D, I am confused as to the difference between these analyses and the earlier data in Figure 1B. It has not been explained what is the definition of "AIM2 inflammasome high" etc (as opposed to "AIM2 high" etc).

The data in Fig 2 are not particularly novel: at least 2 prior reports show LMP1 upregulates IL-1beta, and the role of CTAR1 and CTAR2 in activation via NF-kB is predictable. These data therefore contribute little to the paper.

Figure 3A: Five lines are analysed, and the legend indicates that THP-1 was a positive control. We are not told what is THP-1, nor how it is a positive control (AIM2 and pro-IL-1beta are both negativein THP-1). No negative control line has been included for RIG-I, NLRP3, ASC or caspase-1. The rationale for choosing HK1 for further analyses of IL-1beta induction is unclear, as this was the only NPC line already expressing pro-IL-1beta. Indeed, it is a weakness of the study that most of the key data are derived from just one cell line, which may not be representative.

Figure 4 shows that gamma irradiation and cisplatin activate IL-1beta expression. This by itself is not novel, although it is now shown that the AIM2 inflammasome component is required for irradiation-induced cytokine, whilst NLRP3 required for cisplatin-induced cytokine. Fig 4I is inadequately described; is the third histogram actually showing cells that have been transfected with EBV DNA and EBERs, treated with ATP, H202 and cisplatin, and also irradiated?

Figure 5B-F describes in vivo experiments with a model murine tumor line to demonstrate the well-established fact that IL-1beta can have anti-tumor effects. Whilst unsurprising, interpretation of the dose-responsive effects in the experiment described in Figure 5D (middle panel) is only valid if the outgrowing tumors are shown to contain a mixture of IL-1beta expressing and non-expressing cells.

Figure 6 points to the potentially important conclusion that NPC-derived IL-1beta can induce recruitment/accumulation of tumor-associated neutrophils (TANs) whose presence in NPC is associated with better survival. This observation should be discussed in the light of the previously reported poor prognosis of NPC patients with an elevated neutrophil/lymphocyte ratio (Xin An, 2011, Tumor Biol).

Referee #3 (Comments on Novelty/Model System):

This is a great paper. Every once in a while, one gets to review a really complete piece of work. This is as strong a paper as I've seen in a long time. I think any criticisms will be minor.

Referee #3 (Other Remarks):

Chen et al find that nasopharyngeal carcinoma (NPC) derived IL-1b serves as a major recruiter of host derived anti-tumor neutrophils that directly impacts disease progression in both NPC patients and a related mouse model system. The production of IL-1b is mediated through activation of tumor inflammasome proteins that are either activated under basal conditions or become activated following therapy. Direct activation of inflammasome proteins leading to tumor production of IL-1b is observed in NPC derived cell lines and in B16 melanoma derived model cells.

This is simply an outstanding piece of work. It is amazing in its depth, scope and completeness. If this is what it takes to get papers published these days, we are all in trouble. This is a HUGE amount of work, probably at least 2 huge manuscripts all combined into one report. We have years of patient samples, defining new prognostic markers in a major cancer affecting Asian populations. We have extensive cell line experiments with extremely complete siRNA knockdown experiments demonstrating production and release of IL-1b by NPC cell lines. We have extensive mouse models, including adoptive transfer experiments into IL-1R KO mice, demonstrating the association of tumor production of IL-1b with a host derived neutrophil response leading to tumor regression. And we finish with a direct demonstration of tumor neutrophils in human biopsy specimens. This paper is a tour-de-force of modern molecular medicine.

Of course, there are always things that we would like to see that aren't (or can't) be included. It is too bad that the authors don't have any biopsy material from treated patients to demonstrate directly that therapy for NPC induced a neutrophil dominated inflammatory response. Of course, obtaining biopsies from treated patients is probably not medically indicated, hence the material is not available. This is probably the reason that only 13 of the 140 surveyed tissue biopsies had tumor associated neutrophils in the first place -- these patients must have been undergoing spontaneous inflammatory responses. Presumably, following treatment many more patients would have developed inflammation (as was seen in the mouse model). The mouse modeling experiments are done with B16 melanoma cells, which are of course not NPC cells. But the later can't be used in immunocompenent mice (and there is one experiment with HK1 in nude mice). There is some confusion about why host derived IL-1b may actually promote tumor development -- this is not well addressed in the mouse model. Plus, from an immune point of view it is not clear how neutrophil mediated inflammation leads to tumor clearance -- it is probably related to development of CD8+ CTLs directed against tumor cells (something the authors can address in their next paper using the B16 model). Still, overall, this is an amazing combination of clinical observations and experiments to demonstrate the importance of caspase activation and tumor derived IL-1b in NPC pathogenesis.

It is also clear that this entire model may be restricted to EBV associated tumors, which is well stated by the authors. Still, NPC is a MAJOR malignancy in Asians, hence the overall import is not affected by the view that this phenomena seems to be specific to only one cancer type.

Minor suggestions:

1) Is Fig. 5C lacking a control? B16IL-1b cells into WT? If the authors have this data it can be included.
2) The neutrophil depletion experiments (Fig. 5G) should have been carried out longer, as neutrophil depletion showed an intermediate effect. Do the authors have more data?
3) Fig. 3 -- make sure the font and Y-axis labeling are the same on all graphs. Fig. 3F has a different font and some of the graphs have really crowded Y - axis labeling.
4) The last part of the discussion describes the transcriptional profiling in various tumors to demonstrate that over expression of caspase components is mainly found in NPC and some lymphoma patients. All of this should go into the results section instead of being appended to the end of the Discussion.

Fantastic paper. Thank God I don't work on NPC. The competition is too stiff.

1st Revision - Authors' Response 22 August 2012

Referee #1:

Chen et al. show in their paper that EBV-associated nasopharyngeal carcinoma (NPC) is associated with neutrophil recruitment, where it constitutes a positive prognostic factor. Moreover, inflammasome components (NLRP3, AIM2, RIG-I, ASC, caspase-1) and their product IL-1beta are found to be expressed in NPC specimens, and high mRNA expression levels were associated with better survival in NPC patients. The mechanisms leading to inflammasome activation by EBV have been studied. In an animal model, IL-1beta is suggested to be required for the infiltration of neutrophils, which would mediate antitumor effects. Altogether, the paper contains several convincing datasets that plead in favour of the implication of EBV-induced IL-1beta production in the attraction of neutrophils into the tumour bed.

The following relatively minor problems arise:

- The title of the paper should mention that the tumours are EBV-induced.

We thank the reviewer's comments. In the revised manuscript, we have added "EBV-induced" in the title of the paper. The title of the revised version is "Tumour inflammasome-derived IL-1β recruits neutrophils and improves local recurrence-free survival in EBV-induced nasopharyngeal carcinoma" (page 1, lines 1-3 in the revised version).

- A table summarizing the patient characteristics, as well as the measurement that have been done, should be provided for the supplemental material.

We have added Supporting Information Table S16 and Table S17, which summarize the characteristics of patients analysed by immunohistochemical staining and TANs studies, respectively.

Supporting Information Table S16. Clinicopathologic features of 104 NPC patients used in immunohistochemical staining study

Characteristic	Number of patients
Age[a]	
> Median	50
≤ Median	54
Gender	
Male	79
Female	25
T stage	
1-2	62
3-4	42
N stage	
0-1	60
2-3	44
Clinical stage	
I-II	18
III-IV	86
Histological type	
Keratinizing carcinoma	4
Non-keratinizing carcinoma	
Undifferentiated subtype	86
Differentiated subtype	13
Basaloid squamous cell carcinoma	1
Chemotherapy	
No	69
Yes	35
TANs	
Positive	9
Negative	95

[a] Median age is 45.

Supporting Information Table S17. Clinicopathologic features of 140 NPC patients used in TANs study

Characteristic	Number of patients
Age[a]	
> Median	67
≤ Median	73
Gender	
Male	103
Female	37
T stage	
1-2	86
3-4	54
N stage	
0-1	72
2-3	68
Clinical stage	
I-II	25
III-IV	115
Histological type	
Keratinizing carcinoma	4
Non-keratinizing carcinoma	
Undifferentiated subtype	118
Differentiated subtype	17
Basaloid squamous cell carcinoma	1
Chemotherapy	
No	96
Yes	44
TANs	
Positive	13
Negative	127

[a] Median age is 45.

- All immunoblots lack size markers.

We have added size markers for all immunoblotting data including Fig 2, Fig 3A, Supporting Information Fig S3, Fig S4, Fig S5, Fig S6, and Fig S8 in the revised manuscript.

- The effect of granulocyte depletion demonstrated in Fig. 6G is partial. The authors should comment on this result.

We thank the reviewer's comments. The partial effect of tumour growth by granulocyte depletion may be attributed to the activity of remaining immunostimulatory TANs. In addition, tumour-derived IL-1β may potentially induce dendritic cell-dependent adaptive immunity against tumors, which has been reported by Ghiringhelli F *et al*. (Nature Medicine 15: 1170-1178, 2009). The above description has been added to the Discussion section (page 25, line 17 to page 26, line 1) in the revised version.

- The boxes in Fig. 6H do not correspond to the enlarged panels.

We have corrected the mistakes. Please check the Fig 6H in the revised manuscript.

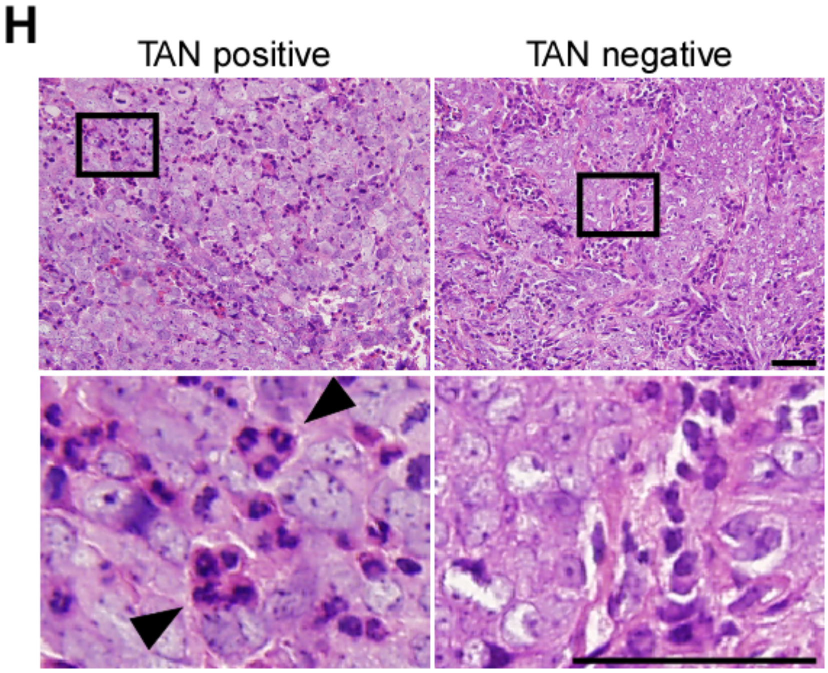

- The authors should attempt a correlation between the density of granulocytes infiltrates in patients and their impact on survival. As it stands, it is not clear how they chose the threshold to separate positive and negative patients.

The cut-off value, > 10 neutrophils/100 epithelial cells, was defined as TANs positive, which was determined from Receiver Operating Characteristics curve analysis. The criteria have also been used in a recent publication by Reid MD *et al*. (Modern Pathology 24: 1612-1619, 2011). This information has been added to the METHODS section, Definition of TANs in NPC patients (page 32, lines 3-6 in the revised version).

- Do the mRNA levels for IL-1beta and different inflammasome components measured in patient samples correlate with the density of the neutrophil infiltrate? This is an important question since it allows to validate or invalidate the working hypothesis proposed by the authors.

We thank the reviewer's comments. Since mRNA samples from the 140 patient samples were not available for analysis, we used the protein staining levels (scores) obtained by immunohistochemistry results to perform the validation analysis. As shown in the revised

manuscript, we have added the Fig 6J and Table S13, which show the positive correlations of the density of the infiltrated neutrophil and the protein levels of IL-1β, AIM2, RIG-I, NLRP3, caspase-1, and ASC in patient samples (page 20, line 18 to page 21, line 2).

J

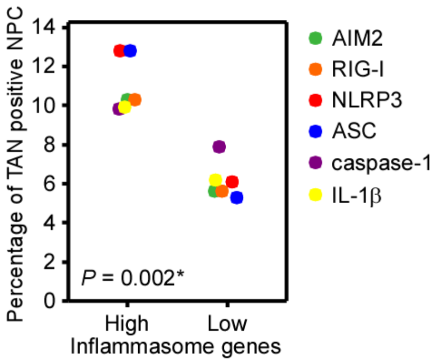

Figure 6. TANs as important effector cells for the IL-1β-mediated antitumor activity. (J) Correlation of TANs and pro-IL-1β and inflammasome components in NPC biopsies. The positive rates of TANs in patients with high expression levels of AIM2, RIG-I, NLRP3, ASC, caspase-1, and IL-1β versus the positive rates of TANs in patients with low expression levels of the above described proteins are analysed using Student's *t* test (Table S13). *$P < 0.002$.

Supporting Information Table S13. Percentage of TAN-positive NPC

	Inflammasome genes	
	High (%)[a]	Low (%)[b]
AIM2	10.3	5.6
RIG-I	10.3	5.6
NLRP3	12.8	6.2
ASC	12.8	5.3
caspase-1	9.8	7.9
IL-1β	9.9	6.1
average	11.0	6.1
Student's *t* test		0.002*

* With statistic significance. [a] The positive rates of TANs in patients with high expression levels of AIM2, RIG-I, NLRP3, ASC, caspase-1, and IL-1β. [b] The positive rates of TANs in patients with low expression levels of the above described proteins.

Referee #2:

This study suggests that inflammasome-triggered IL-1beta production in nasopharyngeal carcinoma (NPC) leads to neutrophil recruitment and favourable prognosis. The key supporting data are summarized in Figure 1 and Figure 6I. Much of the intervening basic biology data are uncontroversial and consistent with a large body of published work.

The study was built on the initial observation that 8/33 inflammosome-related genes were overexpressed in NPC tumours relative to adjacent normal tissues. This is, however, misleading and contradictory, as the data in Table 1 show that only 4 (CIITA, AIM2, ASC and IL-1beta) were significant. The rationale for the inclusion of RIG-1 and NLRP3 in the subsequent experiments is therefore obscure.

We thank the reviewer's comments. To search for the potentially functional genes involved in inflammasomes, we selected the overexpressed genes with expression levels are >2 (tumour versus adjacent normal tissues). By using such criteria, we have included RIG-I (2.212 fold) and NLRP3 (3.645 fold) for further study. The less significant *p* values of RIG-I and NLRP3 are likely due to the smaller sample size analysed in this Q-PCR study. (page 7, lines 9-14 in the revised version)

I also have 2 major concerns regarding the data in Fig 1. First, the definitions for 'Low' and 'High' expression of the inflammasome proteins appear arbitrary and different for each antibody. Secondly, no statistician has been identified in the author contributions; I would therefore be more comfortable if the methodology and the analyses were independently reviewed by an expert statistician.

We thank the reviewer's comments.
(1) The expression levels of each inflammasome proteins were first determined by two pathologists (Chuen Hsueh and Ying Liang, co-authors of this manuscript). Since the nature of each inflammasome protein and the sensitivity of each antibody are different, the cut-off values to define high intensity of IHC staining in tumour cells were determined from Receiver Operating Characteristics curve analysis.

(2) Although no statistician has been listed as co-author, the biostatisticians in the Biostatistics Core of Chang Gung Molecular Medicine Research Center have routinely provided service and consultation to all statistics analyses in this study. Please see the Acknowledgement section. (page 38, line 15 in the revised version)

Regarding Figure 1D, I am confused as to the difference between these analyses and the earlier data in Figure 1B. It has not been explained what is the definition of "AIM2 inflammasome high" etc. (as opposed to "AIM2 high" etc.).

We thank the reviewer's comments. In Figure 1D, "AIM2 inflammasome high" means the levels of all 4 AIM2 inflammasome components including AIM2, ASC, caspase-1, and IL-1β in NPC biopsy tissues are scored as high levels by immunohistochemistry analyses. It is different from "AIM2 high" (Fig 1B), which indicates the elevated or high level of AIM2 protein detected in the NPC tumour cells regardless the expression levels of other components (can be scored as High or Low). The patients with high levels of all 4 components showed better local recurrence-free survival than the patients with low expression levels of all 4 components. Similarly, "RIG-I inflammasome high" or "NLRP3 inflammasome high" means high levels of RIG-I or NLRP3 combined with high levels of ASC, caspase-1, and IL-1β. We have clarified the description in the "Fig 1D legend" of the revised manuscript (page 53, lines 14-19 in the revised version).

The data in Fig 2 are not particularly novel: at least 2 prior reports show LMP1 upregulates IL-1beta, and the role of CTAR1 and CTAR2 in activation via NF-kB is predictable. These data therefore contribute little to the paper.

We thank the reviewer's comments. In the study by Huang YT *et al*. (Head & Neck, 32: 869-876, 2010), LMP1-induced upregulation of IL-1beta was tested in one NPC cell line, NPC-TW01. Our data in Fig 2A showed that LMP1 activates pro-IL-1β in 3 additional NPC cell lines, NPC-TW02, NPC-TW04, and HK1. We also proved the functional role of CTAR1 and CTAR2 in pro-IL-1β induction via NF-κB in NPC cell lines used in our study. We considered this information is very useful for our mechanistic studies of the inflammasome activation in NPC cells (TW01, TW02 and TW04), which only express very low level of pro-IL-1β (Fig. 2A) without induction. Since pro-IL-1β is considered as the Signal 1 of inflammsome activation, thus, LMP1 can provide the Signal 1 for inflammsome activation in these cell lines.

Figure 3A: Five lines are analysed, and the legend indicates that THP-1 was a positive control. We are not told what is THP-1, nor how it is a positive control (AIM2 and pro-IL-1beta are both negative in THP-1). No negative control line has been included for RIG-I, NLRP3, ASC or caspase-1. The rationale for choosing HK1 for further analyses of IL-1beta induction is unclear, as this was the only NPC line already expressing pro-IL-1beta. Indeed, it is a weakness of the study that most of the key data are derived from just one cell line, which may not be representative.

(1) We thank the reviewer's comments. THP-1, a monocyte cell line has been frequently used for inflammasome activation study (Martinon F *et al*., Molecular Cell 10: 417-426, 2002). In this cell line, AIM2 expression is absent, but can be induced by interferon-β (Burckstummer T *et al*., Nature Immunology 10: 266-272, 2009), whereas pro-IL-1b (signal 1 of inflammasome activation) can be induced by LPS (Martinon F *et al*., Molecular Cell 10: 417-426, 2002). Therefore, we used this cell

line as a positive control to demonstrate the presence of some protein components for inflammasome activation. On the other hand, HEK293T, which lack the components for inflammasome activation, was used as a negative control cell (Martinon F *et al*., Molecular Cell 10: 417-426, 2002). In the revised manuscript, we have clarified the reviewer's concern by adding the following sentence : "THP-1 monocytes and HEK293T cells, which have been characterized before for the expression of inflammasome components by western blot (Martinon F *et al*., Molecular Cell 10: 417-426, 2002) were used as positive and negative control for inflammasome activation study, respectively (Fig 3A and Fig S3)." (page 11, lines 10-13 in the revised version).

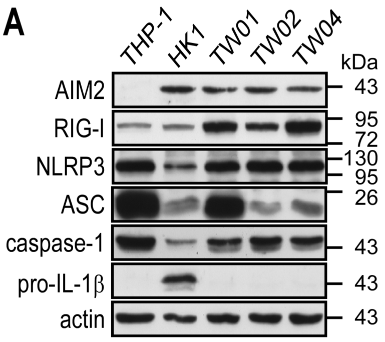

Figure 3. Activation of inflammasomes by EBV-associated factors and microenvironmental stressors. (A) Expression of inflammasome components in NPC cell lines. The protein levels of individual components and actin (loading control) were determined by Western blotting of cell lysates. The THP-1 cells were used as a positive control.

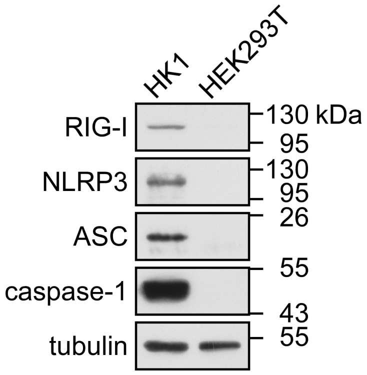

Supporting Information Fig S3. Expression of RIG-I, NLRP3, ASC, and caspase-1 in HK1 cells. The levels of RIG-I, NLRP3, ASC, and caspase-1 protein were determined by Western blotting. Tubulin was used as a loading control. The HEK293T cells were used as a negative control.

(2) HEK293T as the negative control was added in revised version (Fig S3)

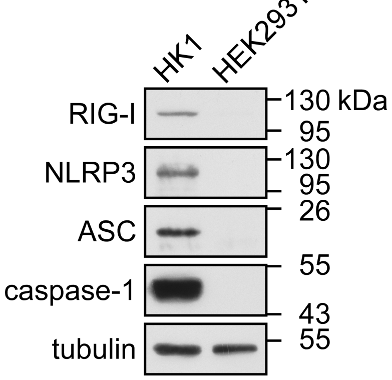

Supporting Information Fig S3. Expression of RIG-I, NLRP3, ASC, and caspase-1 in HK1 cells. The levels of RIG-I, NLRP3, ASC, and caspase-1 protein were determined by Western blotting. Tubulin was used as a loading control. The HEK293T cells were used as a negative control.

(3) We have added the rationale for choosing HK1. The following sentence has been added to the revised manuscript. "After screening four NPC cell lines for expression of key inflammasome components (Fig 3A), we chose HK1 cells for the *in vitro* analysis because the basal expression

level of pro-IL-1β in this cell line is similar to that in NPC biopsies (Fig 1A), and inflammasome activation was easily measured without additional induction of the signal 1, pro-IL-1β. For the other NPC cell lines, the induction of pro-IL-1b was required to provide the signal 1 for the inflammasome activation." (page 11, lines 4-10 in the revised version).

(4) We have added new data about inflammasome activation in another NPC cell line, NPC-TW02 (Fig S5). Similar to the findings in HK1 cells, IL-1β secretion were also induced by various inflammasome stimulators including poly (dA:dT), EBER, ATP, H_2O_2, irradiation, and cisplatin in NPC-TW02 cells. (page 12, lines 12-16; page 14, lines 8-10 in the revised version)

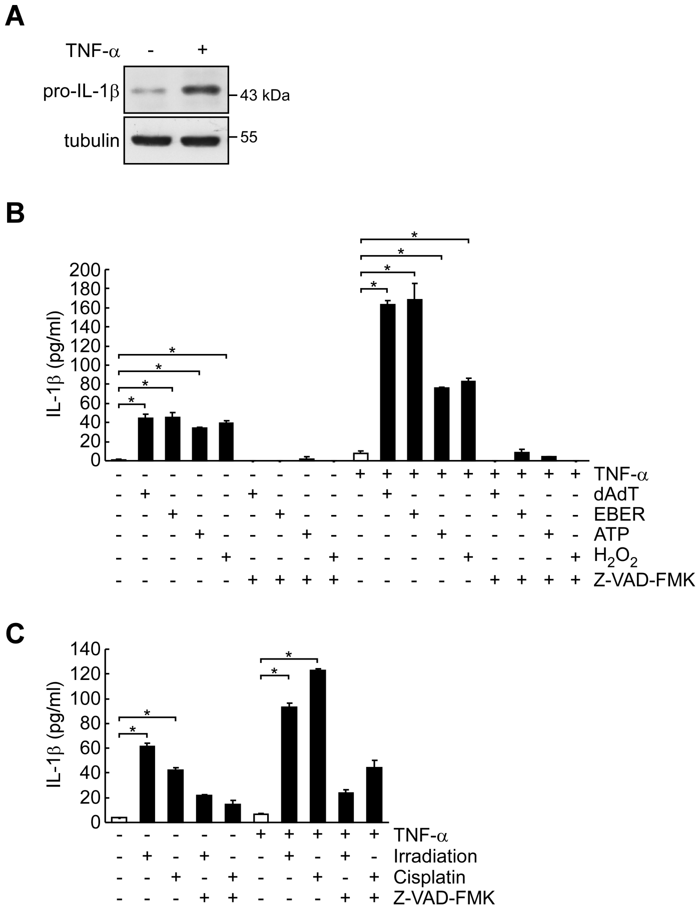

Supporting Information Fig S5. Induction of IL-1β secretion by various inflammasome stimulators in NPC-TW02 cells. (A) Induction of pro-IL-1β expression in NPC-TW02 cell line by TNF-α. NPC-TW02 cells were incubated with 20 ng/ml of TNF-α for 48 h and the protein levels of pro-IL-1β and tubulin (loading control) were determined by Western blotting. (B) IL-1β induction by poly(dA:dT), EBER, ATP, and H_2O_2. NPC-TW02 cells incubated with 20 ng/ml of TNF-α for 48 h were transfected with poly(dA:dT) or with EBER for 12 h or treated with ATP (5 mM) for 4 h or with H_2O_2 (10 μM) for 24 h with or without a pretreatment with Z-VAD-FMK (10 μM) for 30 min. (C) IL-1β induction by irradiation and cisplatin. NPC-TW02 cells incubated with 20 ng/ml of TNF-α for 48 h were treated with irradiation (30 Gy) or cisplatin (40 μM) for 24 h with or without a 30 min pretreatment with Z-VAD-FMK. IL-1β production was used to measure inflammasome activity. All results are presented as the mean ± SD of three experiments. *$P < 0.01$.

Figure 4 shows that gamma irradiation and cisplatin activate IL-1beta expression. This by itself is not novel, although it is now shown that the AIM2 inflammasome component is required for irradiation-induced cytokine, whilst NLRP3 required for cisplatin-induced cytokine. Fig 4I is inadequately described; is the third histogram actually showing cells that have been transfected with EBV DNA and EBERs, treated with ATP, H202 and cisplatin, and also irradiated?

We thank the reviewer's comments. In Fig 4I, the third histogram indeed shows that the cells have been transfected with EBV DNA and EBERs, treated with ATP, H_20_2, cisplatin, and also irradiated.

We have described these in the legend of Fig 4(I) in the original manuscript. "(I) Enhanced induction of tumour microenvironmental factor-stimulated IL-1β by therapeutic treatments. HK1 cells were treated with tumour microenvironmental factors, EBVgDNA/EBERs/ATP/H_2O_2 as previously described with or without therapies, irradiation and cisplatin." (page 57, lines 8-11 in the revised version)

Figure 5B-F describes in vivo experiments with a model murine tumour line to demonstrate the well-established fact that IL-1beta can have anti-tumour effects. Whilst unsurprising, interpretation of the dose-responsive effects in the experiment described in Figure 5D (middle panel) is only valid if the outgrowing tumours are shown to contain a mixture of IL-1beta expressing and non-expressing cells.

We thank the reviewer's comments. We have added the new data in Fig S11 to show that the outgrowing tumours contain a mixture of IL-1β expressing and non-expressing cells (page 16, lines 12-13 in the revised version). As demonstrated in Fig S11, we used PCR with sequence-specific primers to differentiate the two types of cells that were present in the growing tumours.

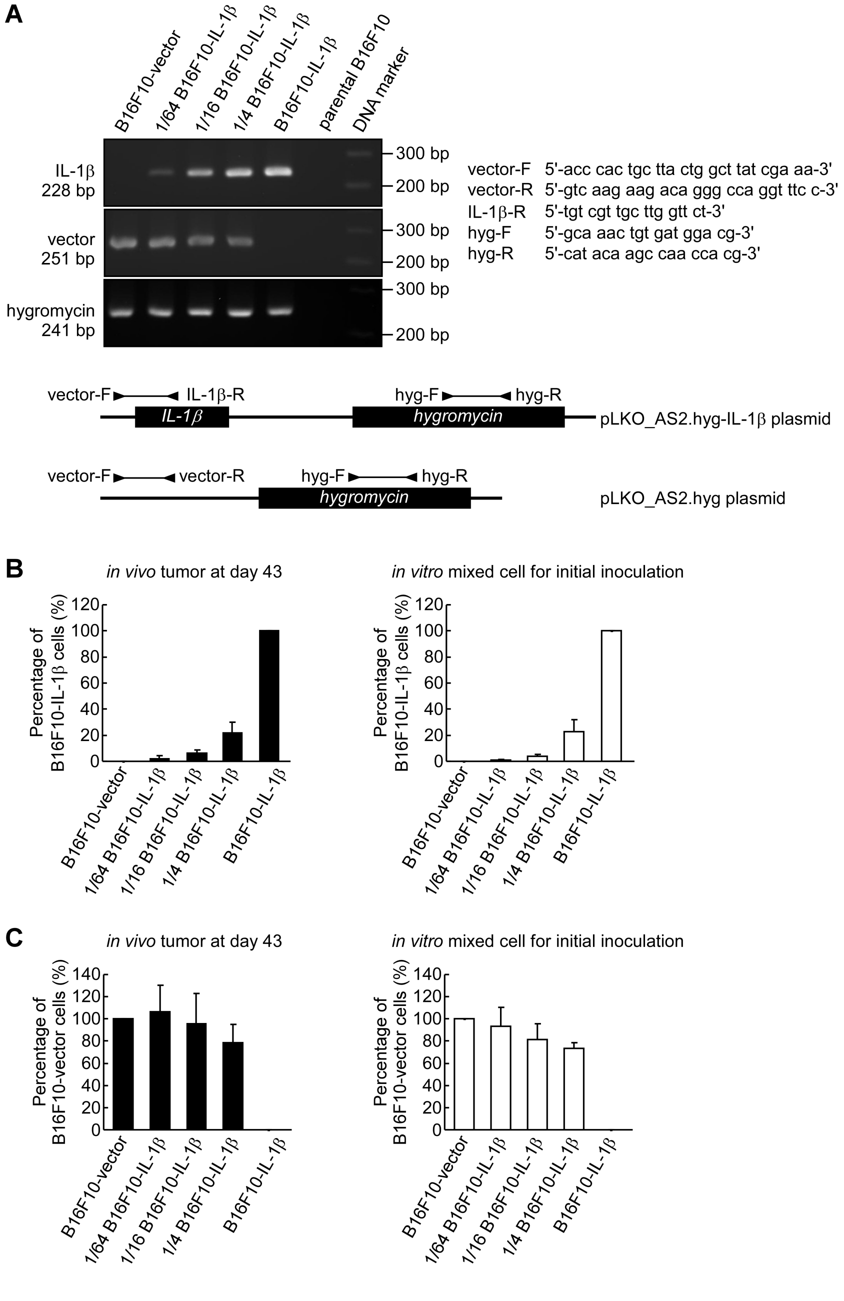

Supporting Information Fig S11. Outgrowing tumours *in vivo* contain both IL-1β expressing (B16F10-IL-1β) and non-expressing (B16F10-vector) cells as determined by specific pairs of primers and quantitative PCR. (A) Establishment of the quantitative PCR method to determine the presence of B16F10-IL-1β and B16F10-vector cells in the mixed cell population. Analysis of B16F10-IL-1β and B16F10-vector cells in the mixed cells was first established by using the cells (B16F10-IL-1β, B16F10-vector, or the mixed) for initial inoculations. Genomic DNA samples prepared from B16F10-IL-1β cells, B16F10-vector cells and a mixture of B16F10-IL-1β and B16F10-vector cells with indicated ratios (1/64, 1/16 and1/4) were amplified by PCR using primers to differentiate the cell backgrounds of each tumour. The B16F10-IL-1β cells were identified by using primers, vector F and IL-1βR (producing a 228-bp PCR product); whereas the B16F10-vector cells were identified by vector F and vector R (producing a 251-bp PCR product). Hygromycin gene located on the pLKO_AS2.hyg plasmid was used as an internal control of B16F10-IL-1β and B16F10-vector cells, and was amplified by using hyg-F and hyg-R primers (producing a 241-bp PCR product). The PCR products amplification yielded after 32 cycles were analysed by agarose gel electrophoresis. Genomic DNA of parental B16F10 cells was used as a negative control. The sequences and positions of primers for PCR assay of the inserted DNA in B16F10-IL-1β cells and B16F10-vector cells are shown. (B) Detection of B16F10-IL-1β cells in the *in vivo* outgrowing tumours. Genomic DNA samples were prepared from the outgrowing tumours harvested at day 43 post-inoculation with B16F10-IL-1β cells, B16F10-vector cells, or mixed cells with indicated ratios. The IL-1β-expressing vector DNA in *in vivo* growing tumours were analysed by quantitative PCR using a pair of primers specific for the B16F10-IL-1β tumour cells, which contained the IL-1β gene-containing pLKO_AS2.hyg plasmid as indicated in (A). The amount of hygromycin gene in each reaction was used as the internal standard. The level of PCR products in tumours initially inoculated with B16F10-IL-1β cells was designated as 100%. Results showed that higher percentages of B16F10-IL-1β cells were detected in *in vivo* tumours of the 1/4 B16F10-IL-1β cells than tumours of the 1/16 and 1/64 cells. The similar results were observed in the mixed cells for initial inoculation. (C) Detection of B16F10-vector cells in the *in vivo* outgrowing tumours. Genomic DNA samples were prepared from the outgrowing tumours harvested at day 43 post-inoculation with B16F10-IL-1β cells, B16F10-vector cells, or mixed cells with indicated ratios as described in (B). The vector DNA in *in vivo* growing tumours were analysed by quantitative PCR using a pair of primers specific for the B16F10-vector tumour cells, which contained the vector pLKO_AS2.hyg plasmid as indicated in (A). The amount of vector-specific PCR products from the tumours derived from B16F10-vector cells were designated as 100%. Results showed that B16F10-vector cells were detected in *in vivo* tumours derived from B16F10-vector cells, or B16F10-vector cells mixed with various ratios (1/4 , 1/16 and 1/64) of B16F10-IL-1β cells. The similar results were observed in the cells (B16F10-IL-1β, B16F10-vector, or the mixed) used for the initial inoculation. The amount of B16F10-vector DNA in tumours was analysed by quantitative PCR and normalized using the PCR level of hygromycin gene in each reaction. The percentages of B16F10-vector cells in mixture cells were similar between in vivo outgrowing tumours and in vitro freshly mixed cells.

Figure 6 points to the potentially important conclusion that NPC-derived IL-1beta can induce recruitment/accumulation of tumour-associated neutrophils (TANs) whose presence in NPC is associated with better survival. This observation should be discussed in the light of the previously reported poor prognosis of NPC patients with an elevated neutrophil/lymphocyte ratio (Xin An, 2011, Tumor Biol).

We thank the reviewer's comments. We have added a paragraph in the "Discussion" section of the revised manuscript (page 26, lines 5-18 in the revised version).
"The correlation of elevated neutrophil to lymphocyte ratio (NLR) in the blood stream and worse survival was recently reported in NPC (An X, *et al.*, Tumor Biology 32: 317-324, 2011). While the underline mechanisms remain elusive, it may be attributed to the following reasons. First, elevated NLR-associated lymphocytopenia may reduce lymphocyte-dependent cytotoxic cell death that is important to antitumor activity. Second, elevated NLR-associated neutrophilic leukocytosis may increase the production of angiogenic growth factors that function to tumour-related angiogenesis and metastasis (An X, *et al.*, Tumor Biology 32: 317-324, 2011). Improving the current treatment for the patients with elevated NLR is important. Our and Fridlender ZG's findings on the recruitment and activation of TANs by IL-1β and TGF-β blockade throw some light on the possibility of conversion of circulating protumor neutrophils to intratumoral antitumor neutrophils (Fridlender ZG *et al.*, Cancer Cell 16: 183-194, 2009). Therefore, it may be worth to take advantage

of antitumor activity of N1 TANs to improve the treatment of the patients with elevated NLR-associated neutrophilic leukocytosis."

Referee #3 (Comments on Novelty/Model System):
This is a great paper. Every once in a while, one gets to review a really complete piece of work. This is as strong a paper as I've seen in a long time. I think any criticisms will be minor.

Referee #3 (Other Remarks):

Chen et al find that nasopharyngeal carcinoma (NPC) derived IL-1b serves as a major recruiter of host derived anti-tumour neutrophils that directly impacts disease progression in both NPC patients and a related mouse model system. The production of IL-1b is mediated through activation of tumour inflammasome proteins that are either activated under basal conditions or become activated following therapy. Direct activation of inflammasome proteins leading to tumour production of IL-1b is observed in NPC derived cell lines and in B16 melanoma derived model cells.

This is simply an outstanding piece of work. It is amazing in its depth, scope and completeness. If this is what it takes to get papers published these days, we are all in trouble. This is a HUGE amount of work, probably at least 2 huge manuscripts all combined into one report. We have years of patient samples, defining new prognostic markers in a major cancer affecting Asian populations. We have extensive cell line experiments with extremely complete siRNA knockdown experiments demonstrating production and release of IL-1b by NPC cell lines. We have extensive mouse models, including adoptive transfer experiments into IL-1R KO mice, demonstrating the association of tumour production of IL-1b with a host derived neutrophil response leading to tumour regression. And we finish with a direct demonstration of tumour neutrophils in human biopsy specimens. This paper is a tour-de-force of modern molecular medicine.

Of course, there are always things that we would like to see that aren't (or can't) be included. It is too bad that the authors don't have any biopsy material from treated patients to demonstrate directly that therapy for NPC induced a neutrophil dominated inflammatory response. Of course, obtaining biopsies from treated patients is probably not medically indicated, hence the material is not available. This is probably the reason that only 13 of the 140 surveyed tissue biopsies had tumour associated neutrophils in the first place -- these patients must have been undergoing spontaneous inflammatory responses. Presumably, following treatment many more patients would have developed inflammation (as was seen in the mouse model). The mouse modelling experiments are done with B16 melanoma cells, which are of course not NPC cells. But the later can't be used in immunocompetent mice (and there is one experiment with HK1 in nude mice). There is some confusion about why host derived IL-1b may actually promote tumour development -- this is not well addressed in the mouse model. Plus, from an immune point of view it is not clear how neutrophil mediated inflammation leads to tumour clearance -- it is probably related to development of CD8+ CTLs directed against tumour cells (something the authors can address in their next paper using the B16 model). Still, overall, this is an amazing combination of clinical observations and experiments to demonstrate the importance of caspase activation and tumour derived IL-1b in NPC pathogenesis.

It is also clear that this entire model may be restricted to EBV associated tumours, which is well stated by the authors. Still, NPC is a MAJOR malignancy in Asians, hence the overall import is not affected by the view that this phenomena seems to be specific to only one cancer type.

The host derived IL-1β that may actually promote tumour development has been reported by many studies (reviewed in Apte RN and Voronov E, Immunological Reviews 222: 222-241, 2008)). As shown in Fig 5C, IL-1β negative B16F10-vector tumours grew faster in wild-type mice than in *Il1r1*$^{-/-}$ mice, suggesting that IL-1β produced by inflammatory cells and stromal cells may promote tumour growth. In addition, the tumour size of B16F10-IL-1β- and B16F10-vector tumours was similar in *Il1r1*$^{-/-}$ mice, indicating that inhibiting effect of tumour-derived IL-1β on tumour growth was dependent on the host response. Furthermore, although we did not have data to address this question, IL-1β may induce tumour angiogenesis via increase of VEGF expression and tumour-mediated immune suppression by myeloid-derived suppressor cells (Apte RN and Voronov E, Immunological Reviews 222: 222-241, 2008).

© EMBO

Activated neutrophils can mediate tumour clearance by cytotoxic activity (Fridlender ZG *et al.*, Cancer Cell 16: 183-194, 2009; Granot Z *et al.*, Cancer Cell 20: 300-314, 2011). Fridlender ZG et al. (2009) show that TGF-β blockade increases the cytotoxicity of tumor-associated neutrophils (TANs) to tumor cells via an oxygen radical-dependent mechanism and these TANs are hypersegmented and express higher levels of proinflammatory cytokines. As shown in Fig 6D, the TANs in B16F10-IL-1β tumours were more lobulated and hypersegmented; in contrast, those in B16F10-vector tumours maintained the characteristic banded appearance typical of blood neutrophils. In addition, the data in Fig 6E show that the levels of arginase mRNA, an important immunosuppressor of the adaptive immune system (Rodriguez PC and Ochoa AC, Immunological Reviews 222: 180-191, 2008), were 5.0-fold lower in TANs from B16F10-IL-1β tumours. In contrast, the mRNA levels of TNF-α, ICAM1, and iNOS, the important immunostimulators, were significantly increased. Our data suggest that tumour-derived IL-1β can polarize TANs toward an antitumor TAN-like immunostimulatory phenotype.

Minor suggestions:

1) Is Fig. 5C lacking a control? B16IL-1b cells into WT? If the authors have this data it can be included.

We thank the reviewer's comments. We have added this data in Fig 5C in revised version.

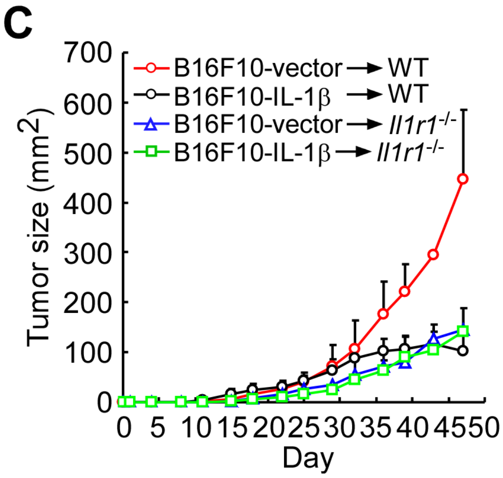

Figure 5. Inhibition of growth by tumour-derived IL-1β *in vivo*. (C) Tumour growth of B16F10-vector and B16F10-IL-1β cells in wild-type and *Il1r1*$^{-/-}$ mice (*n* = 4-6 per group).

2) The neutrophil depletion experiments (Fig. 5G) should have been carried out longer, as neutrophil depletion showed an intermediate effect. Do the authors have more data?

We thank the reviewer's comments. For our animal experiments, we applied Ly6G-specific monoclonal antibody (dose) to mice intraperitoneally, following the dose used in Fridlender ZG *et al.* (Cancer Cell 16: 183-194, 2009). As demonstrated in Fig. 6F, a significant reduction (66%) in TANs from B16F10-IL-1β tumour-bearing mice was noted two weeks after treatment. At this time point (2-week after antibody injection), no difference of tumour growth was observed. Continued injection of the antibody for another 10 days, the ditumourstial growth of tumours between Ly6G- and control antibody-treated was observed (at day 39). The tumour size remained the same at day 43 and day 47 even with another antibody injection. Thus, the intermediate or partial effect of neutrophil depletion on tumour growth may be partly attributed to the activity of remaining immunostimulatory TANs in Fig 6G. In addition, tumour-derived IL-1β may potentially induce dendritic cell-dependent adaptive immunity against tumors, which has been reported by Ghiringhelli F *et al.* (Nature Medicine 15: 1170-1178, 2009). The above description has been added to the Discussion section (page 25, line 17 to page 26, line 1) in the revised version.

3) Fig. 3 -- make sure the font and Y-axis labelling are the same on all graphs. Fig. 3F has a different font and some of the graphs have really crowded Y - axis labelling.

We thank the reviewer's comments. We have corrected this part in revised version.

4) The last part of the discussion describes the transcriptional profiling in various tumours to demonstrate that over expression of caspase components is mainly found in NPC and some lymphoma patients. All of this should go into the results section instead of being appended to the end of the Discussion.

We thank the reviewer's comments. We have modified this part in revised version (page 21, line 6 to page 22, line 3 in the revised version).

Detailed modifications in the cover page, text, and supporting information:

Cover page:

I. TITLE

Modification: "Tumour inflammasome-derived…" (page 1, lines 1-2 in the original version) was changed to "Tumour inflammasome-derived IL-1β recruits neutrophils and improves local recurrence-free survival in EBV-induced nasopharyngeal carcinoma" (page 1, lines 1-3 in the revised version).

Text:

I. RESULTS

Addition: As shown in Fig 6J, the positive rates of TANs were higher in the patients with high level of AIM2, RIG-I, NLRP3, ASC, caspase-1, or IL-1β. To gather statistics, inflammasome genes were significantly correlated with neutrophil infiltration in NPC patients (Table S13). (page 20, line 18 to page 21, line 2).

Addition: THP-1 monocytes and HEK293T cells, which have been characterized before for the presence of inflammasome components by western blot (Martinon et al, 2002) were used as positive and negative control for inflammasome activation study, respectively (Fig 3A and Fig S3). (page 11, lines 10-13 in the revised version).

Addition: After screening four NPC cell lines for expression of key inflammasome components (Fig 3A), we chose HK1 cells for the *in vitro* analysis because the basal expression level of pro-IL-1β in this cell line is similar to that in NPC biopsies (Fig 1A), and inflammasome activation was easily measured without additional induction of the signal 1, pro-IL-1β. For the other NPC cell lines, the induction of pro-IL-1b was required to provide the signal 1 for the inflammasome activation." (page 11, lines 4-10 in the revised version).

Addition: Furthermore, we examined whether inflammasome activation was the general event in NPC cells in the second NPC cell line, NPC-TW02. As shown in Fig S5A and S5B, pro-IL-1β was inducible by TNF-α, and the inflammasome and IL-1β secretion were activated by various inflammasome stimulators, poly (dA:dT), EBER, ATP, and H_2O_2 in NPC-TW02 cells. (page 12, lines 12-16 in the revised version)

Addition: Similar to HK1 cells, the inflammasome and IL-1β secretion were also activated by irradiation and cisplatin in NPC-TW02 cells (Fig S5C). (page 14, lines 8-10 in the revised version)

Addition: The outgrowing tumours indeed contain a mixture of IL-1β expressing and non-expressing cells (Fig S11).

Modification: "In order to …" (page 23, line 13 to page 24, line 17 in the original version) was changed to "Hierarchical cluster analysis of AIM2, RIG-I, and NLRP3 inflammsome gene expression levels in 114 cancer cell lines. In order to delineate the function of inflammasomes in other cancer types, we evaluated the expression patterns of AIM2, RIG-I, NLRP3, ASC, and caspase-1 in 114 cancer cell lines involving 22 cancer types. For this analysis, we used 324 Affymetrix U133 Plus 2.0 array data sets from 8 NPC cell lines generated in this study and 106 cell lines from the public GSK Cancer Cell Line Genomic Profiling Data (Greshock et al, 2010) according to a distance tree obtained through hierarchical cluster analysis. The cancer cell lines were separated into 4 clusters (Fig S12A and Table S14), and all 8 NPC cell lines were clustered together and were closely grouped with 9 of 17

lymphoma and 2 of 8 brain cancer cell lines (designated as cluster 1) but not with cell lines of other cancer types (Fig S12B-S12C). Since both lymphoma and NPC are associated with EBV infection, we found that high levels of AIM2, RIG-I, and NLRP3 inflammasome expression is significantly correlated with EBV infection in lymphoma cell lines ($P = 0.043$; Fig S12D), suggesting that EBV infection is a potential factor for AIM2, RIG-I, and NLRP3 inflammasome overexpression in cancer cells of both epithelial and lymphocyte origin. (page 21, line 6 to page 22, line 3 in the revised version).

II. DISCUSSION

Addition: The partial effect of granulocyte depletion on recovering tumour growth may be attributed to the activity of remaining immunostimulatory TANs. In addition, tumour-derived IL-1β may potentially induce dendritic cell-dependent adaptive immunity against tumors (Ghiringhelli et al, 2009). (page 25, line 17 to page 26, line 1 in the revised version).

Addition: The correlation of elevated neutrophil to lymphocyte ratio (NLR) in the blood stream and worse survival was recently reported in NPC (An et al, 2011). While the underline mechanisms remain elusive, it may be attributed to the following reasons. First, elevated NLR-associated lymphocytopenia may reduce lymphocyte-dependent cytotoxic cell death that is important to antitumor activity. Second, elevated NLR-associated neutrophilic leukocytosis may increase the production of angiogenic growth factors that function to tumour-related angiogenesis and metastasis (An et al, 2011). Improving the current treatment for the patients with elevated NLR is important. Our and Fridlender ZG's findings on the recruitment and activation of TANs by IL-1β and TGF-β blockade throw some light on the possibility of conversion of circulating protumor neutrophils to intratumoural antitumor neutrophils (Fridlender et al, 2009). It may be worth to take the advantage of antitumor activity of N1 TANs to improve the treatment of the patients with elevated NLR-associated neutrophilic leukocytosis. (page 26, lines 5-18 in the revised version)

III. METHODS

Addition: The cut-off value, > 10 neutrophils/100 epithelial cells, was defined as TANs positive, which was determined from Receiver Operating Characteristics curve analysis. The criteria have also been used in a recent publication by Reid MD *et al* (Reid et al, 2011). (page 32, lines 3-6 in the revised version).

IV. Acknowledgments

Modification: "We thank …" (page 33, lines 14-16 in the original version) was changed to "We thank the Pathology Core and Biostatistic Core of the Chang Gung Molecular Medicine Research Center for technical support." (page 38, lines 14-16 in the revised version).

V. References

Addition: An X, Ding PR, Wang FH, Jiang WQ, Li YH (2011) Elevated neutrophil to lymphocyte ratio predicts poor prognosis in nasopharyngeal carcinoma. Tumour Biol 32: 317-324.

Addition: Martinon F, Burns K, Tschopp J (2002) The inflammasome: a molecular platform triggering activation of inflammatory caspases and processing of proIL-beta. Mol Cell 10: 417-426.

VI. Figure legends

Addition: AIM2 inflammasome high means the levels of all 4 AIM2 inflammasome components including AIM2, as well as 3 common components ASC, caspase-1, and IL-1β in NPC biopsy tissues are scored as high levels by immunohistochemistry analyses. Similarly, RIG-I inflammasome high or NLRP3 inflammasome high means high levels of RIG-I or NLRP3 combined with high levels of ASC, caspase-1, and IL-1β." (page 53, lines 14-19 in the revised version).

VII. FIGURE

Modification: Fig 2. Addition of immunoblotting size marker
Modification: Fig 3A. Addition of immunoblotting size marker
Modification: Fig 5C. Integration of the data of B16F10-IL-1β cells in Wild-type mice into Fig 5C.
Modification: Fig 6H. Correction of the boxes
Addition: Figure 6J. Correlation of TANs and pro-IL-1β and inflammasome components in NPC biopsies.

Supporting information:

Addition: Fig S3. Expression of RIG-I, NLRP3, ASC, and caspase-1 in HK1 cells.
Modification: Fig S4. Addition of immunoblotting size marker.
Addition: Fig S5. Induction of IL-1β secretion by various inflammasome stimulators in NPC-TW02 cells.
Modification: Fig S6. Addition of immunoblotting size marker.
Modification: Fig S8. Addition of immunoblotting size marker.
Addition: Fig S11. Outgrowing tumours *in vivo* contain both IL-1β expressing (B16F10-IL-1β) and non-expressing (B16F10-vector) cells as determined by specific pairs of primers and quantitative PCR.
Addition: Supporting information. Table S13. Percentage of TAN-positive NPC
Addition: Supporting information. Table S16. Clinicopathologic features of 104 NPC patients used in immunohistochemical staining study.
Addition: Supporting information. Table S17. Clinicopathologic features of 140 NPC patients used in TANs study.

Detailed modifications in Figures and Tables

Original version	Revised version	Change
	Fig 6J	New addition
	Fig S3	New addition
Fig S3	Fig S4	
	Fig S5	New addition
Fig S4	Fig S6	
Fig S5	Fig S7	
Fig S6	Fig S8	
Fig S7	Fig S9	
Fig S8	Fig S10	
	Fig S11	New addition
Fig S9	Fig S12	
	Table S13	New addition
Table S13	Table S14	
Table S14	Table S15	
	Table S16	New addition
	Table S17	New addition
Table S15	Table S18	
Table S16	Table S19	
Table S17	Table S20	
Table S18	Table S21	
Table S19	Table S22	

2nd Editorial Decision 31 August 2012

Thank you for the submission of your revised manuscript "Tumor inflammasome-derived IL-1beta recruits neutrophils and improves local recurrence-free survival in EBV-induced nasopharyngeal carcinoma" to EMBO Molecular Medicine. We have now received the reports from the reviewer who was asked to re-review your manuscript.

You will be glad to see that the reviewer is now globally supportive and we can proceed with official acceptance of your manuscript pending the minor changes detailed below:

- For experiments involving human subjects the submission must include a statement that informed consent was obtained from all subjects and that the experiments conformed to the principles set out in the WMA Declaration of Helsinki [http://www.wma.net/en/30publications/10policies/b3/] and the NIH Belmont Report [http://ohsr.od.nih.gov/guidelines/belmont.html]. Please see our Guide to Authors for further information and provide the necessary information in the respective Material and Methods part.

- The description of all reported data that includes statistical testing must state the name of the statistical test used to generate error bars and P values, the number (n) of independent experiments underlying each data point (not replicate measures of one sample), and the actual P value for each test (not merely 'significant' or 'P < 0.05').

I look forward to seeing a revised version of your manuscript as soon as possible.

Yours sincerely,

Editor
EMBO Molecular Medicine

***** Reviewer's comments *****

Referee #2:

The authors have made a thorough job of addressing previous criticisms.

2nd Revision - Authors' Response 07 September 2012

The specific changes are listed as follows.
1) *For experiments involving human subjects the submission must include a statement that informed consent was obtained from all subjects and that the experiments conformed to the principles set out in the WMA Declaration of Helsinki [http://www.wma.net/en/30publications/10policies/b3/] and the NIH Belmont Report [http://ohsr.od.nih.gov/guidelines/belmont.html]. Please see our Guide to Authors for further information and provide the necessary information in the respective Material and Methods part.*

We have replaced “Informed consent was obtained from all patients.” (page 30, line 9 in the original version) to “Informed consent was obtained from all subjects and that the experiments conformed to the principles set out in the WMA Declaration of Helsinki [http://www.wma.net/en/30publications/10policies/b3/] and the NIH Belmont Report [http://ohsr.od.nih.gov/guidelines/belmont.html].” in the revised version (page 30, line 9-12 in the revised version).

2) *The description of all reported data that includes statistical testing must state the name of the statistical test used to generate error bars and P values, the number (n) of independent experiments underlying each data point (not replicate measures of one sample), and the actual P value for each test (not merely 'significant' or 'P < 0.05').*

We have added the name of the statistical test used to generate error bars and P values, the number of independent experiments underlying each data point, and the actual P value for each test in all statistical data in the revised version.

ü
