# Supplementary material for: Tumour inflammasome-derived IL-1β recruits neutrophils and improves local recurrence-free survival in EBV-induced nasopharyngeal carcinoma
Source: EMBO Mol Med. 2012 Oct 15;4(12):1276–93. doi: 10.1002/emmm.201201569 (PMC3531603; doi:10.1002/emmm.201201569)
Supplement: Supplementary file 2 [file emmm0004-1276-SD2.pdf]

Supplementary Information

Tumor inflammasome-derived IL-1β recruits neutrophils and improves local recurrence-free survival in EBV-induced nasopharyngeal carcinoma

Lih-Chyang Chen, Li-Jie Wang, Nang-Ming Tsang, David M. Ojcius, Chia-Chun Chen, Chun-Nan OuYang, Chuen Hsueh, Ying Liang, Kai-Ping Chang, Chiu-Chin Chen, and Yu-Sun Chang

Table of contents

Figure S1: Overexpression and the association of CIITA, NLRC4, and NLRP7 in NPC tumor cells with LRFS and DFS in NPC patients..3
Figure S2: Kaplan-Meier survival analysis of DFS as a function of elevated inflammasome component expression in NPC patients..5
Figure S3: Expression of RIG-I, NLRP3, ASC, and caspase-1 in HK1 cells................6
Figure S4: Knockdown of AIM2, RIG-I, NLRP3, and cathepsin B in HK1 cells.........7
Figure S5: Induction of IL-1β secretion by various inflammasome stimulators in NPC-TW02 cells..8
Figure S6: Association of endogenous ASC with AIM2, RIG-I, and NLRP3 in HK1 cells in response to PAMPs and DAMPs..10
Figure S7: The effect of irradiation and cisplatin treatments on ROS production and ATP release..11
Figure S8: Association of endogenous ASC with AIM2 and NLRP3 in HK1 cells in response to treatments..13
Figure S9: Characterization of IL-1β-producing HK1 and B16F10 cell lines.............14
Figure S10: Tumor-derived IL-1β can inhibit tumor growth *in vivo*. Nude mice were injected into intrafootpad with B16F10-pro-IL-1β and B16F10-IL-1β cells 16
Figure S11: Outgrowing tumors *in vivo* contain both IL-1β expressing (B16F10-IL-1β) and non-expressing (B16F10-vector) cells as determined by specific pairs of primers and quantitative PCR 17
Figure S12: Hierarchical cluster analysis of AIM2, RIG-I, and NLRP3 inflammsome gene expression levels in 114 cancer cell lines..21
Table S1: Relationship between ASC and clinicopathologic features.........................24
Table S2: Relationship between caspase-1 and clinicopathologic features.................25
Table S3: Relationship between IL-1β and clinicopathologic features 26
Table S4: Relationship between AIM2 and clinicopathologic features.......................27
Table S5: Relationship between RIG-I and clinicopathologic features.......................28
Table S6: Relationship between NLRP3 and clinicopathologic features 29

Table S7: Multivariate analysis of the association between ASC and local recurrence-free survival of NPC patients..30
Table S8: Multivariate analysis of the association between caspase 1 and local recurrence-free survival of NPC patients..31
Table S9: Multivariate analysis of the association between IL-1β and local recurrence-free survival of NPC patients..32
Table S10: Multivariate analysis of the association between AIM2 and local recurrence-free survival of NPC patients..33
Table S11: Multivariate analysis of the association between RIG-I and local recurrence-free survival of NPC patients..34
Table S12: Multivariate analysis of the association between NLRP3 and local recurrence-free survival of NPC patients..35
Table S13: Percentage of TAN-positive NPC..36
Table S14: Cluster of 114 cancer cell lines by expression profile of AIM2, RIG-I, NLRP3, ASC, and Caspase-1..37
Table S15: Clinicopathologic features of 144 NPC patients used in this study...........41
Table S16: Clinicopathologic features of 104 NPC patients used in immunohistochemical staining study..42
Table S17: Clinicopathologic features of 140 NPC patients used in TANs study.......43
Table S18: Antibodies used for immunohistochemical staining.................................44
Table S19: Antibodies used for FACS analysis 45
Table S20: Antibodies used for immunoprecipitation and Western Blotting...............46
Table S21: Sequence of quantitative PCR Primers..47
Table S22: Sequence of siRNA Primers 50

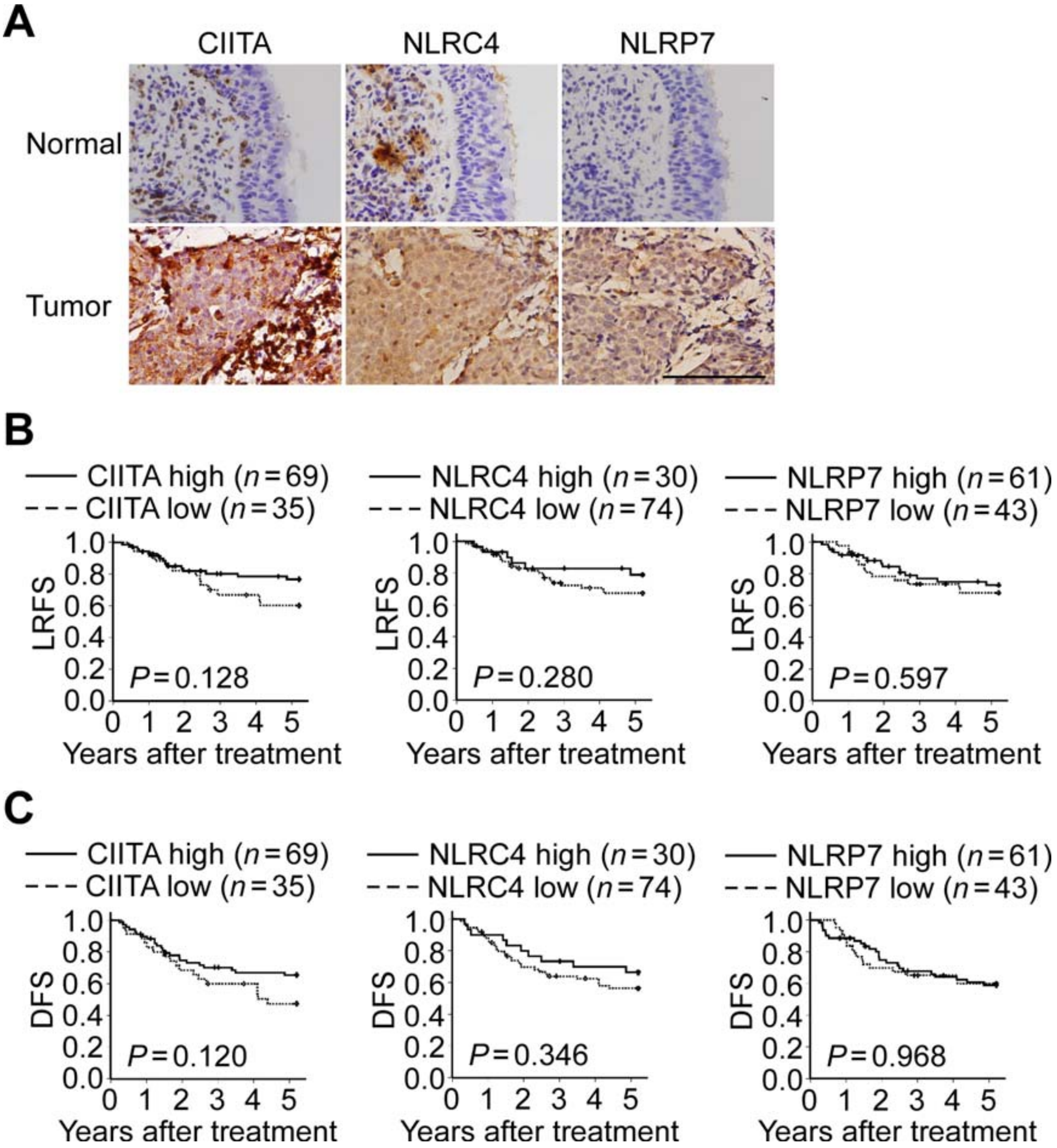

Supporting Information Fig S1. Overexpression and the association of CIITA, NLRC4, and NLRP7 in NPC tumor cells with LRFS and DFS in NPC patients. (A) Overexpression of CIITA, NLRC4, and NLRP7 proteins in NPC tumor cells. Consecutive NPC tissue sections containing tumor (lower panel) and adjacent nontumor cells (upper panel) were immunohistochemically stained with

protein-specific antibodies. The results are shown at 400x magnification. The expression of CIITA, NLRC4, and NLRP7 was stronger in the tumor cells than the adjacent normal nasopharyngeal epithelial cells. Bar, 100 μm. Kaplan-Meier survival analysis of LRFS (B) and DFS (C) according to the levels of CIITA, NLRC4, and NLRP7 expression in NPC patients. No correlation of CIITA, NLRC4, and NLRP7 with survival was found in NPC patients.

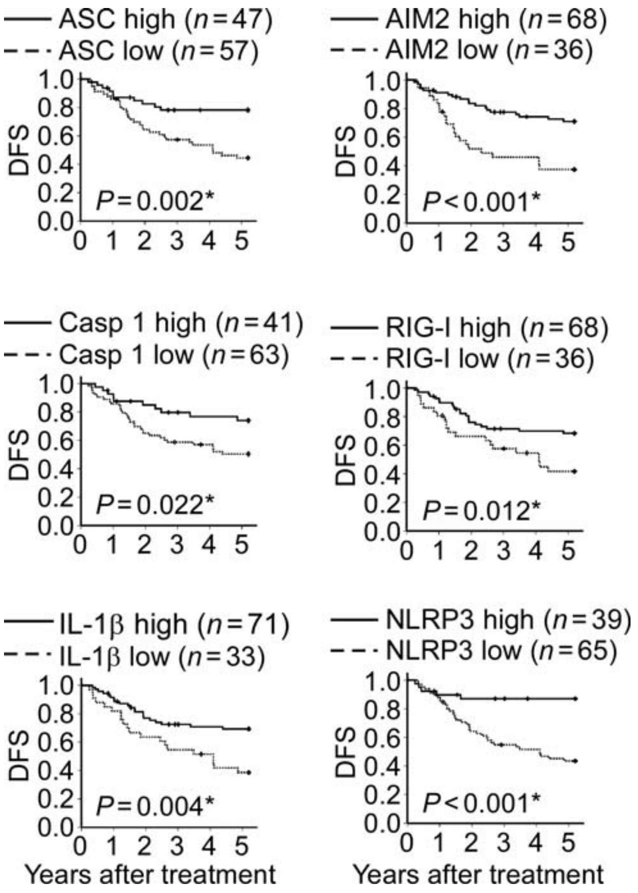

Supporting Information Fig S2. Kaplan-Meier survival analysis of DFS as a function of elevated inflammasome component expression in NPC patients. * With statistic significance as indicated.

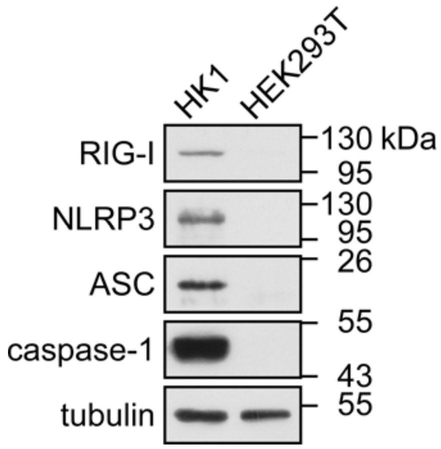

Supporting Information Fig S3. Expression of RIG-I, NLRP3, ASC, and caspase-1 in HK1 cells. The levels of RIG-I, NLRP3, ASC, and caspase-1 protein were determined by Western blotting. Tubulin was used as a loading control. The HEK293T cells were used as a negative control.

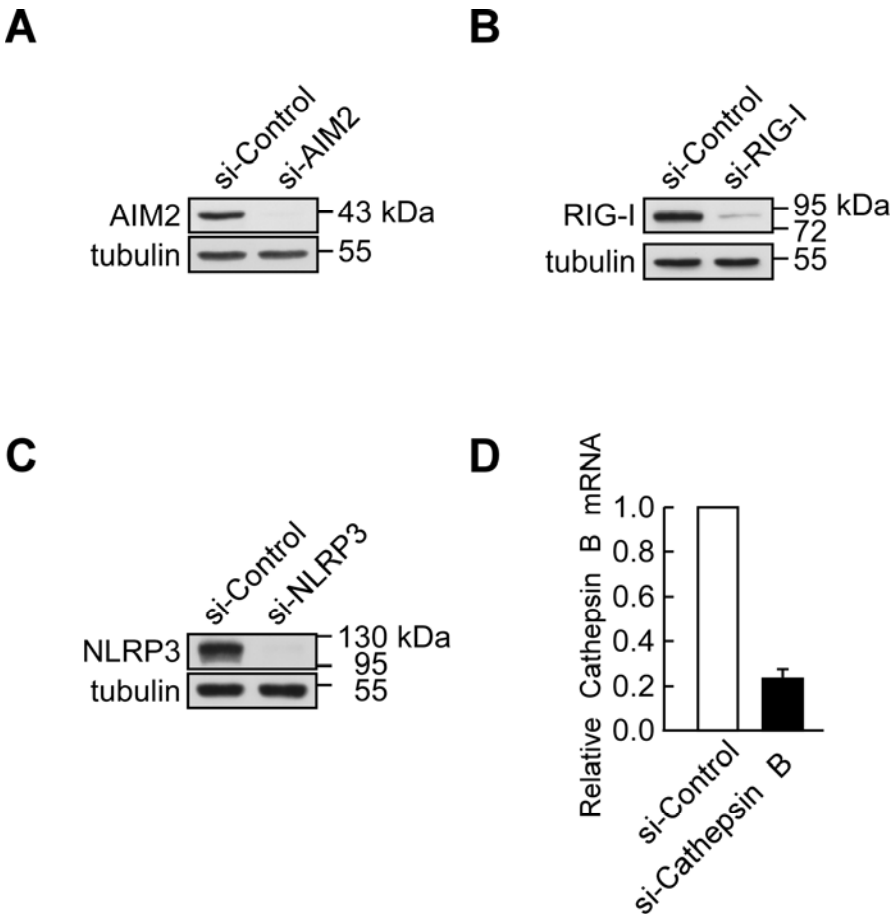

Supporting Information Fig S4. Knockdown of AIM2, RIG-I, NLRP3, and cathepsin B in HK1 cells. (A-C) Knockdown of AIM2, RIG-I, and NLRP3 expression in HK1 cells. HK1 cells transfected with AIM2- (A), RIG-I- (B), or NLRP3 siRNA (C) were cultured for 48 h. The levels of AIM2, RIG-I, and NLRP3 protein was determined by Western blotting. Tubulin was used as a loading control. (D) Knockdown of cathepsin B expression in HK1 cells. HK1 cells transfected with the control and cathepsin B siRNA were cultured for 48 h. The level of cathepsin B mRNA was determined by quantitative RT-PCR. The results are presented as the mean ± SD of three independent experiments.

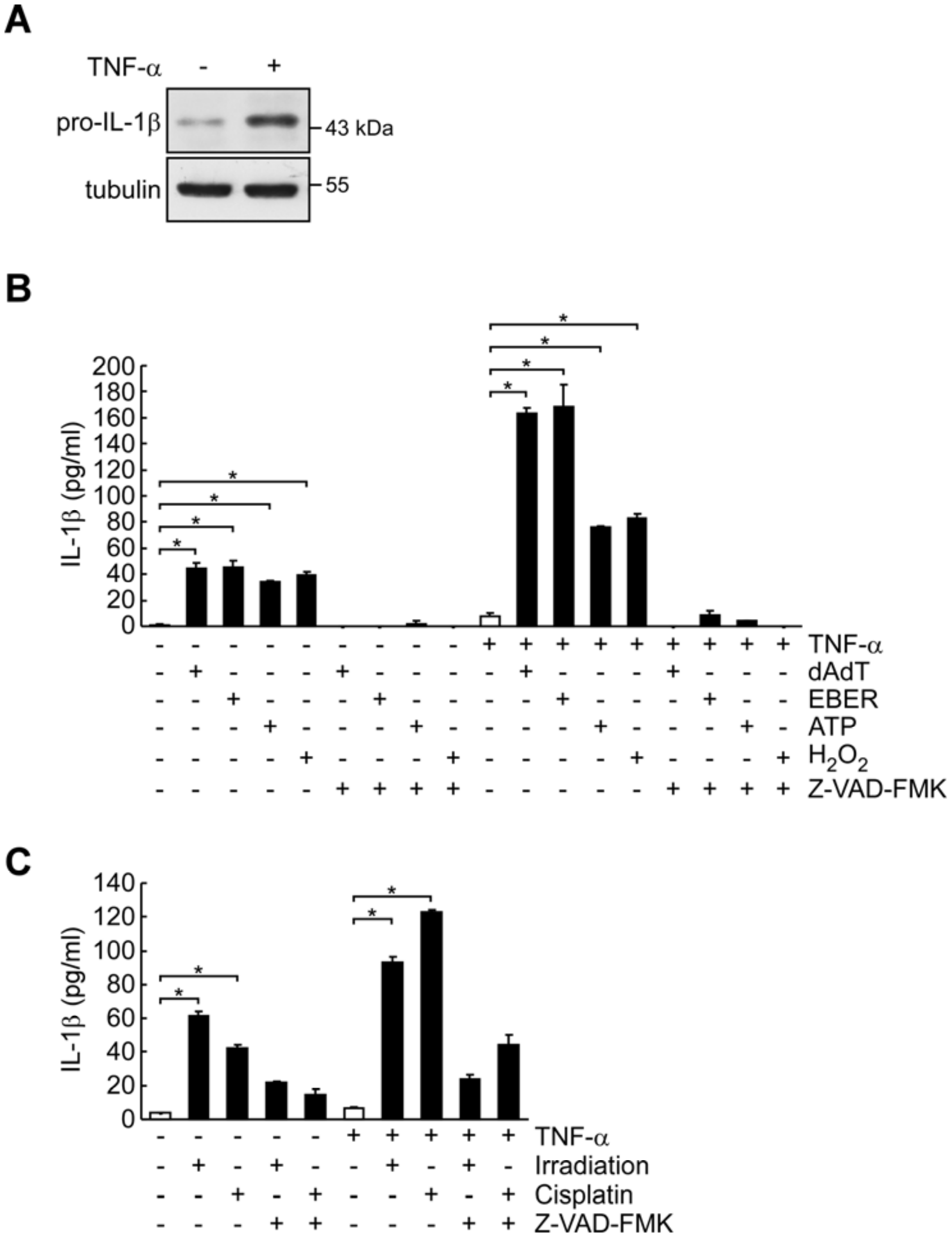

Supporting Information Fig S5. Induction of IL-1β secretion by various inflammasome stimulators in NPC-TW02 cells. (A) Induction of pro-IL-1β expression in NPC-TW02 cell line by TNF-α. NPC-TW02 cells were incubated with 20 ng/ml of TNF-α for 48 h and the protein levels of pro-IL-1β and tubulin (loading

control) were determined by Western blotting. (B) IL-1β induction by poly(dA:dT), EBER, ATP, and H_2O_2. NPC-TW02 cells incubated with 20 ng/ml of TNF-α for 48 h were transfected with poly(dA:dT) or with EBER for 12 h or treated with ATP (5 mM) for 4 h or with H_2O_2 (10 μM) for 24 h with or without a pretreatment with Z-VAD-FMK (10 μM) for 30 min. Without TNF-α treatment, *P = 0.005, 0.006, 0.001, and 0.003 for poly(dA:dT), EBER, ATP, and H_2O_2, respectively; With TNF-α treatment, *P = 0.0001, 0.003, 0.0001, and 0.002 for poly(dA:dT), EBER, ATP, and H_2O_2, respectively. All results are presented as the mean ± SD of three independent experiments and analyzed by Student's *t* test. (C) IL-1β induction by irradiation and cisplatin. NPC-TW02 cells incubated with 20 ng/ml of TNF-α for 48 h were treated with irradiation (30 Gy) or cisplatin (40 μM) for 24 h with or without a 30 min pretreatment with Z-VAD-FMK. IL-1β production was used to measure inflammasome activity. Without TNF-α treatment, *P = 0.005 and 0.003 for irradiation and cisplatin, respectively; With TNF-α treatment, *P = 0.0003 and 0.0001 for irradiation and cisplatin, respectively. All results are presented as the mean ± SD of three independent experiments and analyzed by Student's *t* test. .

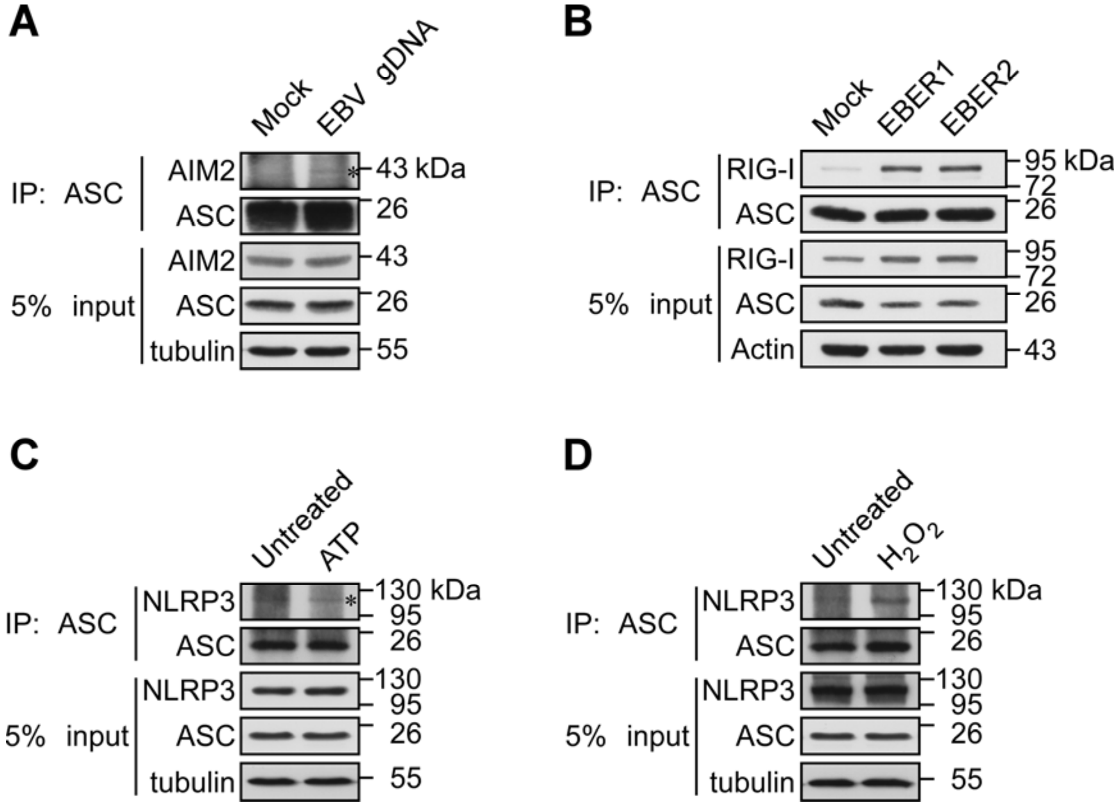

Supporting Information Fig S6. Association of endogenous ASC with AIM2, RIG-I, and NLRP3 in HK1 cells in response to PAMPs and DAMPs. Lysates of HK1 cells treated with EBV gDNA (A), EBER1 and EBER2 (B), ATP (C), or H_2O_2 (D) were immunoprecipitated (IP) with an ASC-specific monoclonal antibody. The proteins of AIM2, RIG-I, NLRP3, and ASC in the lysates or the immunoprecipitates were visualized by Western blotting with protein-specific antibodies. Tubulin and actin were used as a loading control. * indicates the specific band.

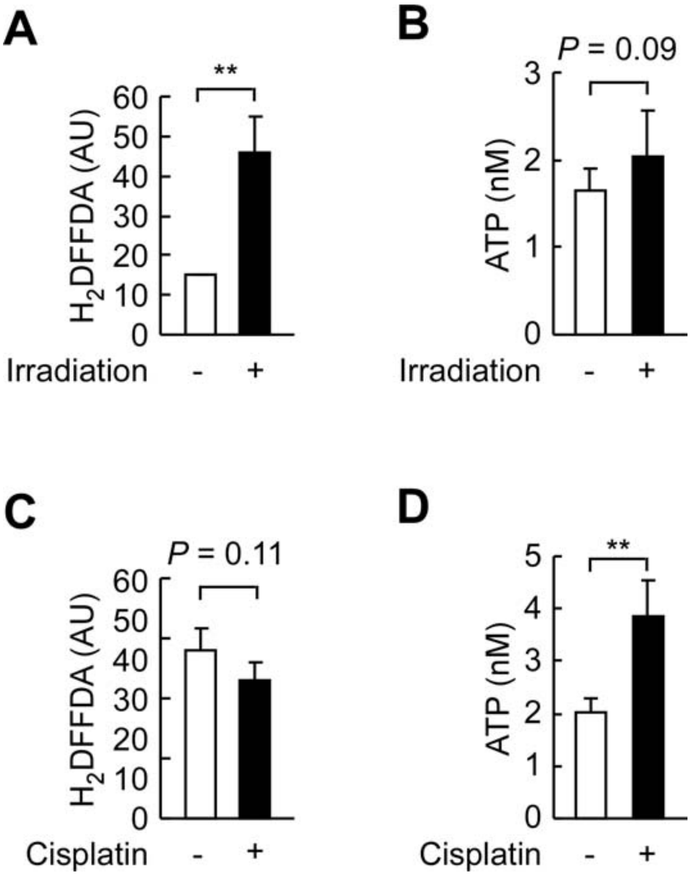

Supporting Information Fig S7. The effect of irradiation and cisplatin treatments on ROS production and ATP release. (A) The effect of irradiation on ROS production. HK1 cells treated with 30 Gy of irradiation were cultured for 1 h. The level of cellular ROS was assessed by ROS-specific fluorescent probe H_2DFFDA (10 μM; Invitrogen, Carlsbad, CA) according to the manufacturer's instructions. Fluorescence was assessed with a SpectraMax M2 plate reader (Molecular Devices) using an excitation/emission of 485/523 nm. $^{**}P = 0.007$, the results are presented as the mean ± SD of four independent experiments and analyzed by Student's *t* test. AU,

arbitrary units. (B) The effect of irradiation on ATP release. HK1 cells treated with 30 Gy of irradiation were cultured for 24 h. The concentration of ATP in cultured medium was measured immediately by luciferin-based ENLITEN ATP Assay (Promega, Madison, USA) according to the manufacturer's instructions. The luciferase activity was measured using the Dual-Glo Luciferase Assay System (Promega) as an indicator for ATP content. The results are presented as the mean ± SD of four independent experiments and analyzed by Student's *t* test. (C) The effect of cisplatin on ROS production. HK1 cells treated with 40 mM of cisplatin were cultured for 1 h. The level of cellular ROS was assessed by the ROS-specific fluorescent probe H_2DFFDA. The results are presented as the mean ± SD of four independent experiments and analyzed by Student's *t* test. AU, arbitrary units. (D) The effect of cisplatin on ATP release. HK1 cells treated with 40 mM of cisplatin were cultured for 24 h. The concentration of ATP in cultured medium was measured immediately. $^{**}P = 0.00001$, the results are presented as the mean ± SD of eight independent experiments and analyzed by Student's *t* test.

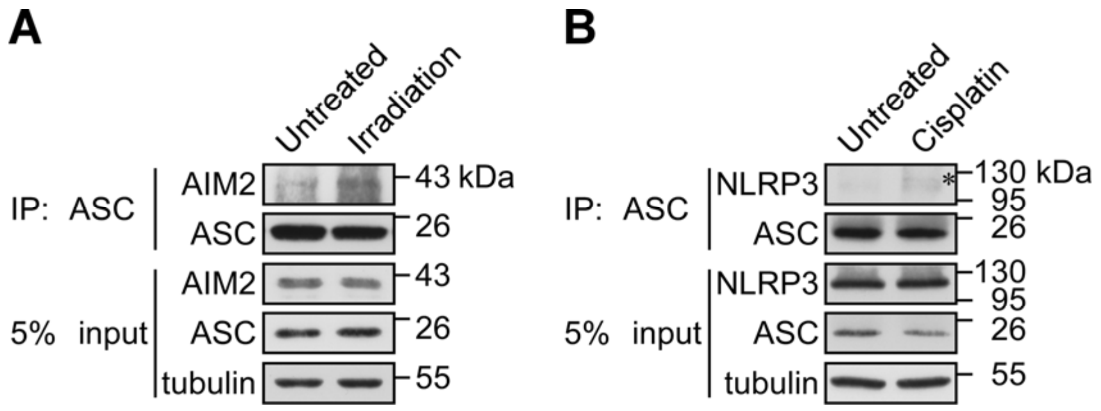

Supporting Information Fig S8. Association of endogenous ASC with AIM2 and NLRP3 in HK1 cells in response to treatments. Lysates of HK1 cells treated with irradiation (A) or cisplatin (B) were immunoprecipitated (IP) with an ASC-specific monoclonal antibody. The proteins of AIM2, NLRP3, and ASC in the lysates or the immunoprecipitates were visualized by Western blotting with protein-specific antibodies. Tubulin was used as a loading control. * indicates the specific band.

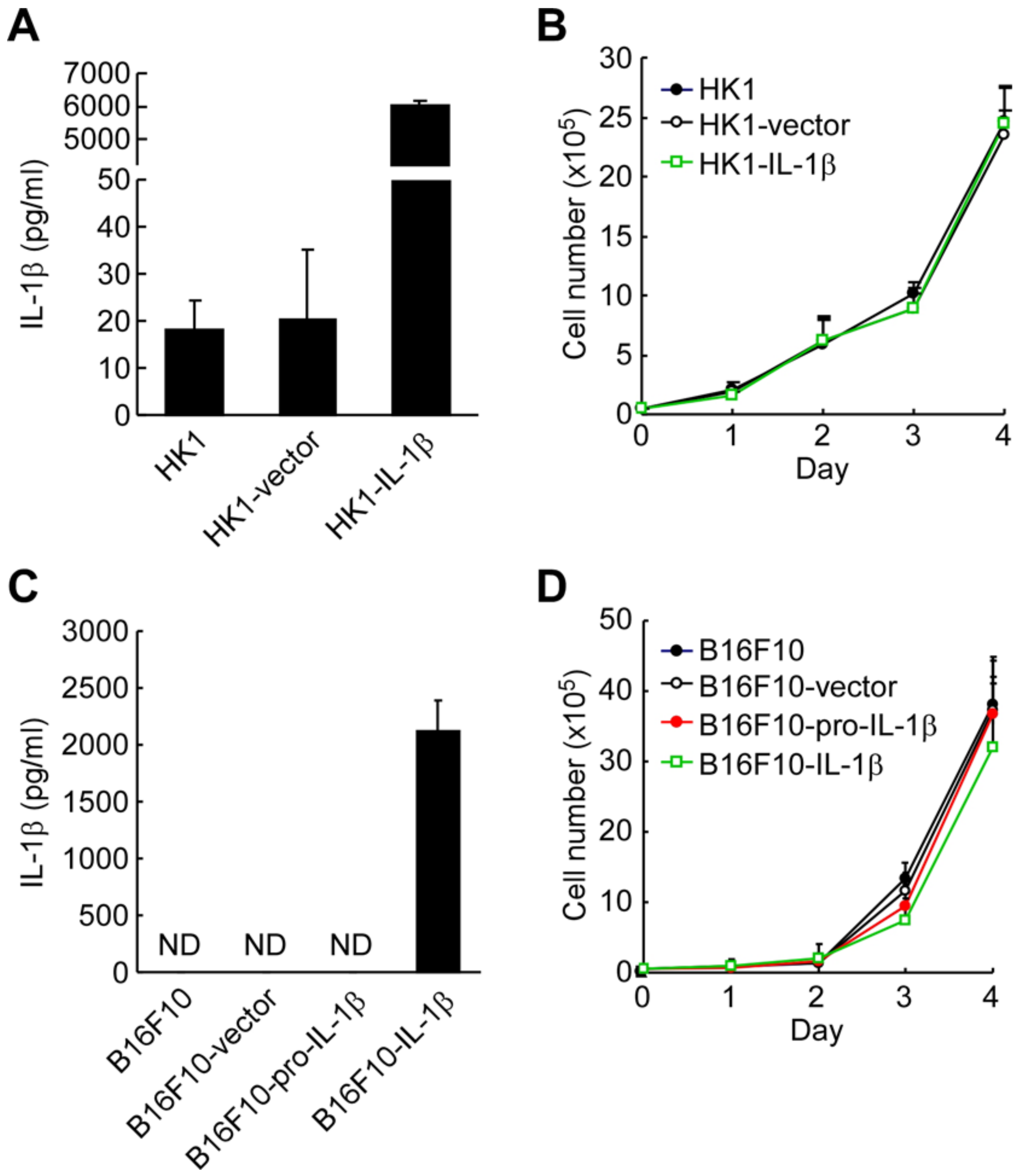

Supporting Information Fig S9. Characterization of IL-1β-producing HK1 and B16F10 cell lines. (A) Detection of IL-1β in supernatants from the HK1-IL-1β and control cells. HK1 cells infected with lentiviruses containing IL-1β gene (HK1-IL-1β) and empty vector (HK1-vector), or the parental HK1 cells were incubated for 48 h, and supernatants were analyzed for IL-1β content by ELISA. (B) Cell growth kinetics

of HK1-IL-1β cells. HK1, HK1-vector, and HK1-IL-1β cells were cultured and then cell numbers were determined at indicated days. (C) Detection of IL-1β in supernatants from the B16F10 IL-1β and control cells. Parental (B16F10), empty vector-transduced (B16F10-vector), pro-IL-1β-transduced (B16F10-pro-IL-1β), and IL-1β-transduced (B16F10-IL-1β) cells were cultured for 48 h, and supernatants were analyzed for IL-1β content by ELISA. ND, no detectable IL-1β. (D) Cell growth kinetics of B16F10-IL-1β and control cells. B16F10, B16F10-vector, B16F10-pro-IL-1β, and B16F10-IL-1β cells were cultured and then cell numbers were determined at indicated days. All results are presented as the mean ± SD of three independent experiments; bars, SD.

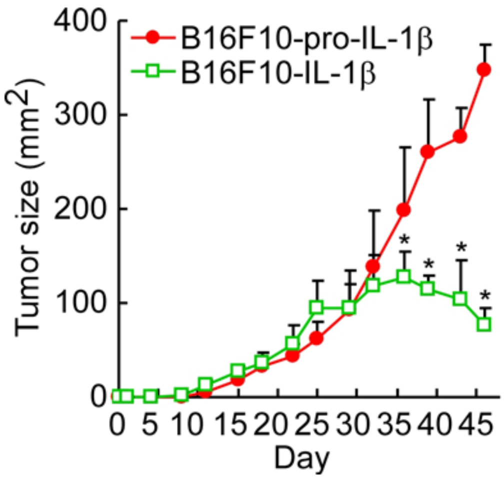

Supporting Information Fig S10. Tumor-derived IL-1β can inhibit tumor growth *in vivo*. Nude mice were injected into intrafootpad with B16F10-pro-IL-1β and B16F10-IL-1β cells. Each group included 8 mice, and each experiment was repeated at least twice with similar results. Tumor size was measured twice a week with a caliper. **P* = 7.8E-03, 1.3E-06, 6.5E-07, and 1.2E-12 at day 36, 39, 43, and 46 post-inoculation, respectively. All results are presented as the mean ± SD and analyzed by Student's *t* test.

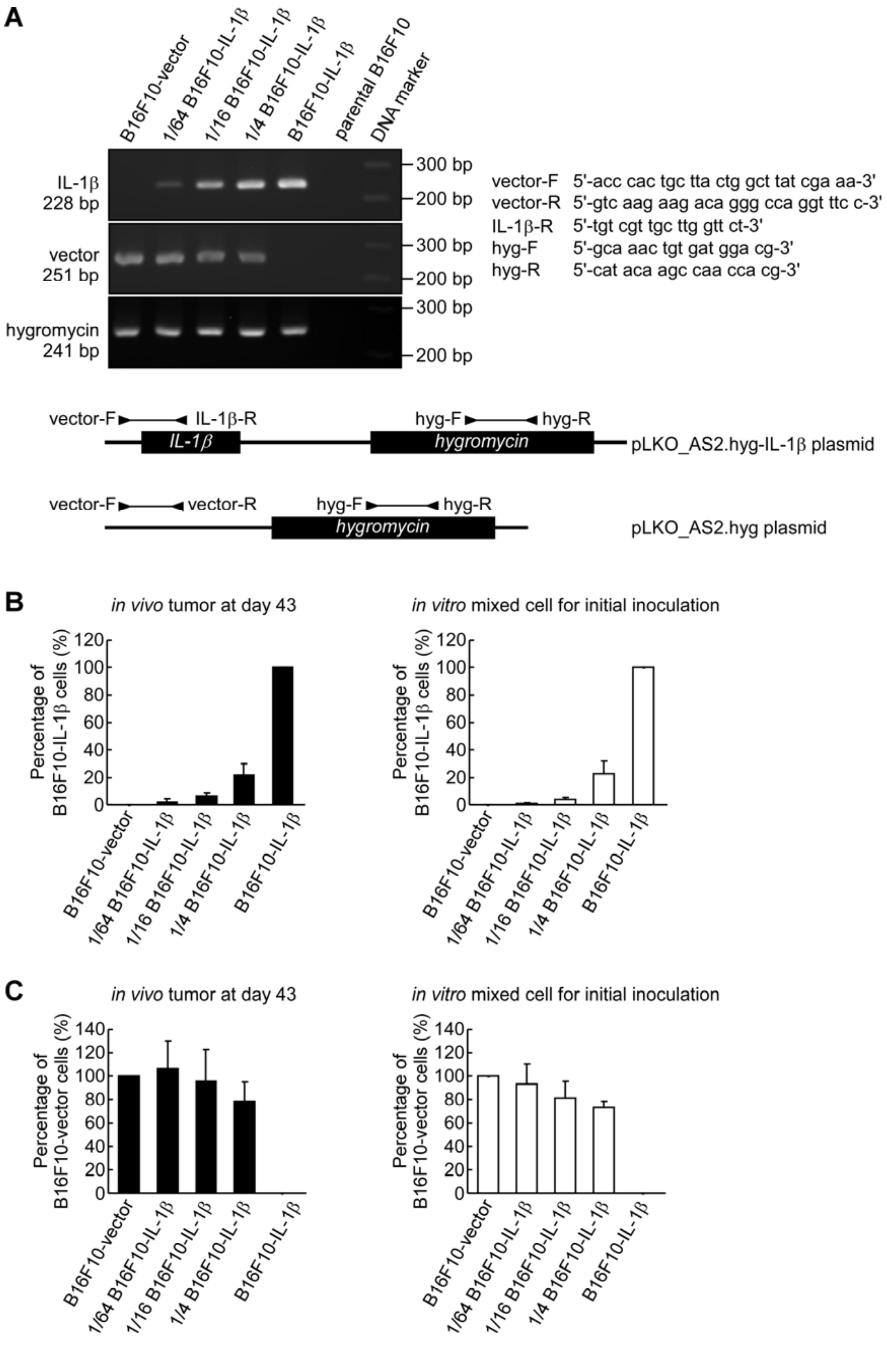
A
B16F10-vector
1/64 B16F10-IL-1β
1/16 B16F10-IL-1β
1/4 B16F10-IL-1β
B16F10-IL-1β
parental B16F10
DNA marker
IL-1β 228 bp
vector 251 bp
hygromycin 241 bp
300 bp
200 bp
vector-F 5'-acc cac tgc tta ctg gct tat cga aa-3'
vector-R 5'-gtc aag aag aca ggg cca ggt ttc c-3'
IL-1β-R 5'-tgt cgt tgc ttg gtt ct-3'
hyg-F 5'-gca aac tgt gat gga cg-3'
hyg-R 5'-cat aca agc caa cca cg-3'
vector-F
IL-1β-R
hyg-F
hyg-R
IL-1β
hygromycin
pLKO_AS2.hyg-IL-1β plasmid
vector-R
pLKO_AS2.hyg plasmid
B
in vivo tumor at day 43
in vitro mixed cell for initial inoculation
Percentage of B16F10-IL-1β cells (%)
C
Percentage of B16F10-vector cells (%)

Supporting Information Fig S11. Outgrowing tumors *in vivo* contain both IL-1β expressing (B16F10-IL-1β) and non-expressing (B16F10-vector) cells as determined by specific pairs of primers and quantitative PCR. (A) Establishment of the quantitative PCR method to determine the presence of B16F10-IL-1β and B16F10-vector cells in the mixed cell population. Detection of B16F10-IL-1β and B16F10-vector cells in the mixed cells was first established by using the cells (B16F10-IL-1β, B16F10-vector, or the mixed) for initial inoculations. Genomic DNA samples prepared from B16F10-IL-1β cells, B16F10-vector cells and a mixture of B16F10-IL-1β and B16F10-vector cells with indicated ratios (1/64, 1/16 and1/4) were amplified by PCR using primers to differentiate the cell backgrounds of each tumor. The B16F10-IL-1β cells were identified by using primers, vector F and IL-1βR (producing a 228-bp PCR product); whereas the B16F10-vector cells were identified by vector F and vector R (producing a 251-bp PCR product). Hygromycin gene located on the pLKO_AS2.hyg plasmid was used as an internal control of B16F10-IL-1β and B16F10-vector cells, and was amplified by using hyg-F and hyg-R primers (producing a 241-bp PCR product). The PCR products amplification yielded after 32 cycles were analyzed by agarose gel electrophoresis. Genomic DNA of parental B16F10 cells was used as a negative control. The sequences and positions of primers for PCR assay of the inserted DNA in B16F10-IL-1β cells and B16F10-vector

cells are shown. (B) Detection of B16F10-IL-1β cells in the *in vivo* outgrowing tumors. Genomic DNA samples were prepared from the outgrowing tumors harvested at day 43 post-inoculation with B16F10-IL-1β cells, B16F10-vector cells, or mixed cells with indicated ratios. The IL-1β-expressing vector DNA in *in vivo* growing tumors were analyzed by quantitative PCR using a pair of primers specific for the B16F10-IL-1β tumor cells, which contained the IL-1β gene-containing pLKO_AS2.hyg plasmid as indicated in (A). The amount of hygromycin gene in each reaction was used as the internal standard. The level of PCR products in tumors initially inoculated with B16F10-IL-1β cells was designated as 100%. Results showed that higher percentages of B16F10-IL-1β cells were detected in *in vivo* tumors of the 1/4 B16F10-IL-1β cells than tumors of the 1/16 and 1/64 cells. The similar results were observed in the mixed cells for initial inoculation. (C) Detection of B16F10-vector cells in the *in vivo* outgrowing tumors. Genomic DNA samples were prepared from the outgrowing tumors harvested at day 43 post-inoculation with B16F10-IL-1β cells, B16F10-vector cells, or mixed cells with indicated ratios as described in (B). The vector DNA in *in vivo* growing tumors were analyzed by quantitative PCR using a pair of primers specific for the B16F10-vector tumor cells, which contained the vector pLKO_AS2.hyg plasmid as indicated in (A). The amount of vector-specific PCR products from the tumors derived from B16F10-vector cells

were designated as 100%. Results showed that B16F10-vector cells were detected in *in vivo* tumors derived from B16F10-vector cells, or B16F10-vector cells mixed with various ratios (1/4 , 1/16 and 1/64) of B16F10-IL-1β cells. The similar results were observed in the cells (B16F10-IL-1β, B16F10-vector, or the mixed) used for the initial inoculation. The amount of B16F10-vector DNA in tumors was analyzed by quantitative PCR and normalized using the PCR level of hygromycin gene in each reaction. The percentages of B16F10-vector cells in mixture cells were similar between in vivo outgrowing tumors and in vitro freshly mixed cells. In animal experiments, each group included 4 mice, and each experiment was repeated twice with similar results. In cell culture experiments, the results were obtained from three independent experiments. All results are given as means ± SD.

A

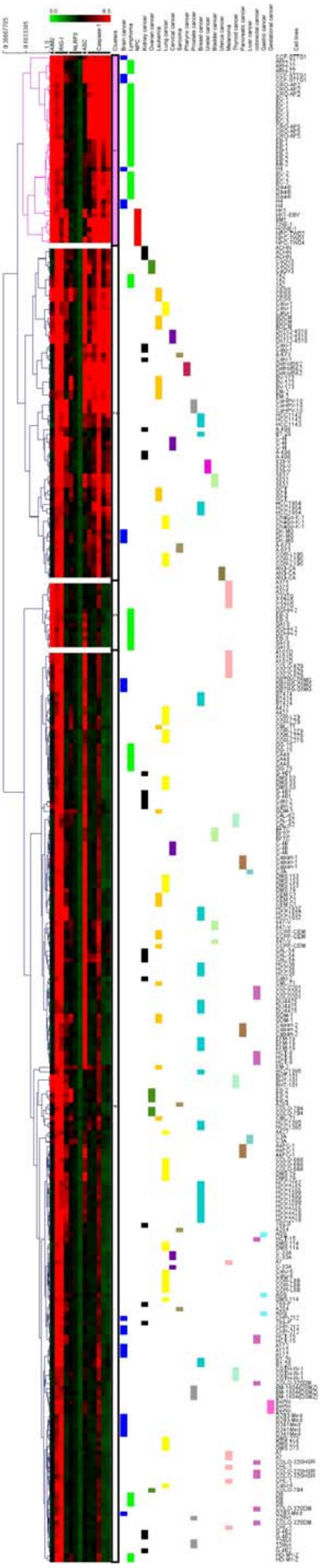

B

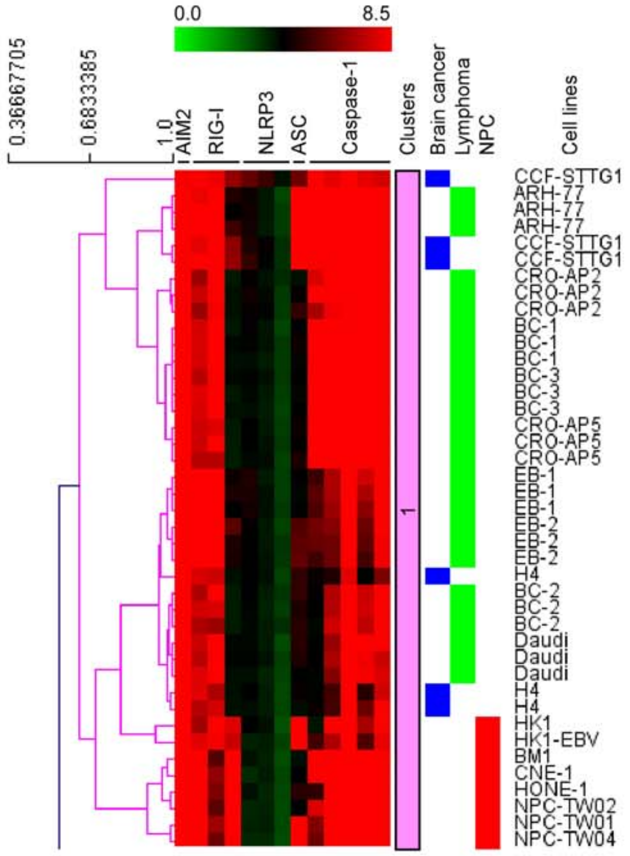

C

Expression of AIM2, RIG-I, and NLRP3 inflammasomes in various types of cancer

Cell lines	High level	%
NPC	8/8	100.0
Lymphoma	9/17	52.9
Brain cancer	2/8	25.0
Cervical cancer	0/4	0.0
Leukemia	0/9	0.0
Lung cancer	0/14	0.0
Breast cancer	0/12	0.0
Kidney cancer	0/8	0.0
Melanoma	0/6	0.0
Colorectal cancer	0/5	0.0
Bladder cancer	0/3	0.0
Ovarian cancer	0/3	0.0
Pancreatic cancer	0/3	0.0
Prostate cancer	0/3	0.0
Thyroid cancer	0/3	0.0
Sarcoma	0/2	0.0
Gastric cancer	0/1	0.0
Liver cancer	0/1	0.0
Pharynx cancer	0/1	0.0
Gestational cancer	0/1	0.0
Ureter cancer	0/1	0.0
Uterus cancer	0/1	0.0

D

Correlation of AIM2, RIG-I, and NLRP3 inflammasome expression and EBV infection in lymphoma cell lines

	Inflammasome		
	High	Low	*P*
EBV			
Positive	8	3	0.043*
Negative	1	5	

Supporting Information Fig S12. Hierarchical cluster analysis of AIM2, RIG-I, and NLRP3 inflammsome gene expression levels in 114 cancer cell lines. (A) Hierarchical cluster analysis grouped the 114 cell lines into 4 clusters (Supplementary Table 14). Using the 13 probes of Affymetrix U133 Plus 2.0 array corresponding to AIM2, RIG-I, NLRP3, ASC, and caspase-1 genes of inflammasomes, 8 NPC cell lines and 9 out of 17 lymphoma and 2 of 8 brain cancer cell lines cell lines were clustered together, therefore designated as Cluster 1 (marked in Pink color). The cell lines did not group together when 13 random, non-gene-specific probes were tested. (B) A zoom-out Custer 1 of the hierarchical cluster analysis in (A). (C) Summary of results from the cell lines from 22 cancer types. (D) Correlation of AIM2, RIG-I, and NLRP3 inflammasome expression and EBV infection in 9 lymphoma cell lines grouped in Cluster 1. *P*-value was calculated by the Pearson Chi-Square test.

Methods

Inflammsome gene profiling analyses with microarray data from cancer cell lines

The expression profiles of inlfammsome genes in 8 NPC cell lines (HK1, HK1-EBV, BM-1, CNE-1, HONE-1, NPC-TW01, NPC-TW02, and NPC-TW04) were analyzed using Human Genome U133 Plus 2.0 array (Affymetrix, Santa Clara, CA, USA) in the National Yang-Ming University Genomics Center, Taiwan. The Human Genome

U133 Plus 2.0 array data of other cancer cell lines were retrieved from the GSK Cancer Cell Line Genomic Profiling Data (https://array.nci.nih.gov/caarray/project/woost-00041). The probes corresponding to AIM2, RIG-I, NLRP3, ASC, and caspase-1 were imported into TIGR MultiExperiment Viewer (MeV) (http://www.tm4.org/mev/) to carry out clustering analysis. Hierarchical clustering was performed based on Pearson correlation distance and average linkage rule.

Supporting Information Table S1. Relationship between ASC and clinicopathologic features

Characteristics	Total (*n*)	ASC High	ASC Low	*P*
Age[a]				0.330
> Median	50	20	30	
≤ Median	54	27	27	
Gender				0.253
Male	79	33	46	
Female	25	14	11	
T stage				0.237
1-2	62	25	37	
3-4	42	22	20	
N stage				0.550
0-1	60	29	31	
2-3	44	18	26	
Clinical stage				0.307
I-II	18	6	12	
III-IV	86	41	45	
Histological type				0.179
Keratinizing carcinoma	4	1	3	
Non-keratinizing carcinoma				
Undifferentiated subtype	86	42	44	
Differentiated subtype	13	3	10	
Basaloid squamous cell carcinoma	1	1	0	
Chemotherapy				0.098
No	69	27	42	
Yes	35	20	15	

[a] Median age is 45. Relationship between ASC expression and clinicopathologic characteristics was evaluated using the Pearson Chi-Square test. No significant correlation was seen between ASC and any of the examined clinicopathological features.

Supporting Information Table S2. Relationship between caspase-1 and clinicopathologic features

Characteristics	Total (*n*)	Caspase1		*P*
		High	Low	
Age[a]				0.842
> Median	50	19	31	
≤ Median	54	22	32	
Gender				1.000
Male	79	31	48	
Female	25	10	15	
T stage				0.414
1-2	62	22	40	
3-4	42	19	23	
N stage				0.104
0-1	60	28	32	
2-3	44	13	31	
Clinical stage				1.000
I-II	18	7	11	
III-IV	86	34	52	
Histological type				0.503
Keratinizing carcinoma	4	1	3	
Non-keratinizing carcinoma				
Undifferentiated subtype	86	35	51	
Differentiated subtype	13	4	9	
Basaloid squamous cell carcinoma	1	1	0	
Chemotherapy				1.000
No	69	27	42	
Yes	35	14	21	

[a] Median age is 45. Relationship between caspase-1 expression and clinicopathologic characteristics was evaluated using the Pearson Chi-Square test. No significant correlation was seen between caspase-1 and any of the examined clinicopathological features.

Supporting Information Table S3. Relationship between IL-1β and clinicopathologic features

Characteristics	Total (*n*)	IL-1β High	IL-1β Low	*P*
Age[a]				0.095
> Median	50	30	20	
≤ Median	54	41	13	
Gender				0.218
Male	79	51	28	
Female	25	20	5	
T stage				0.392
1-2	62	40	22	
3-4	42	31	11	
N stage				0.209
0-1	60	44	16	
2-3	44	27	17	
Clinical stage				1.000
I-II	18	12	6	
III-IV	86	59	27	
Histological type				0.288
Keratinizing carcinoma	4	3	1	
Non-keratinizing carcinoma				
Undifferentiated subtype	86	61	25	
Differentiated subtype	13	6	7	
Basaloid squamous cell carcinoma	1	1	0	
Chemotherapy				0.662
No	69	46	23	
Yes	35	25	10	

[a] Median age is 45. Relationship between IL-1β expression and clinicopathologic characteristics was evaluated using the Pearson Chi-Square test. No significant correlation was seen between IL-1β and any of the examined clinicopathological features.

Supporting Information Table S4. Relationship between AIM2 and clinicopathologic features

Characteristics	Total (*n*)	AIM2		*P*
		High	Low	
Age[a]				0.306
> Median	50	30	20	
≤ Median	54	38	16	
Gender				1.000
Male	79	52	27	
Female	25	16	9	
T stage				0.149
1-2	62	37	25	
3-4	42	31	11	
N stage				0.836
0-1	60	40	20	
2-3	44	28	16	
Clinical stage				0.174
I-II	18	9	9	
III-IV	86	59	27	
Histological type				0.395
Keratinizing carcinoma	4	3	1	
Non-keratinizing carcinoma				
Undifferentiated subtype	86	58	28	
Differentiated subtype	13	7	6	
Basaloid squamous cell carcinoma	1	0	1	
Chemotherapy				0.391
No	69	43	26	
Yes	35	25	10	

[a] Median age is 45. Relationship between AIM2 expression and clinicopathologic characteristics was evaluated using the Pearson Chi-Square test. No significant correlation was seen between AIM2 and any of the examined clinicopathological features.

Supporting Information Table S5. Relationship between RIG-I and clinicopathologic features

Characteristics	Total (*n*)	RIG-I		*P*
		High	Low	
Age[a]				1.000
> Median	50	33	17	
≤ Median	54	35	19	
Gender				0.235
Male	79	49	30	
Female	25	19	6	
T stage				0.303
1-2	62	38	24	
3-4	42	30	12	
N stage				0.299
0-1	60	42	18	
2-3	44	26	18	
Clinical stage				0.786
I-II	18	11	7	
III-IV	86	57	29	
Histological type				0.685
Keratinizing carcinoma	4	3	1	
Non-keratinizing carcinoma				
Undifferentiated subtype	86	57	29	
Differentiated subtype	13	7	6	
Basaloid squamous cell carcinoma	1	1	0	
Chemotherapy				0.513
No	69	47	22	
Yes	35	21	14	

[a] Median age is 45. Relationship between RIG-I expression and clinicopathologic characteristics was evaluated using the Pearson Chi-Square test. No significant correlation was seen between RIG-I and any of the examined clinicopathological features.

Supporting Information Table S6. Relationship between NLRP3 and clinicopathologic features

Characteristics	Total (*n*)	NLRP3		*P*
		High	Low	
Age[a]				0.313
> Median	50	16	34	
≤ Median	54	23	31	
Gender				1.000
Male	79	30	49	
Female	25	9	16	
T stage				0.838
1-2	62	24	38	
3-4	42	15	27	
N stage				0.100
0-1	60	18	42	
2-3	44	21	23	
Clinical stage				0.793
I-II	18	6	12	
III-IV	86	33	52	
Histological type				0.266
Keratinizing carcinoma	4	2	2	
Non-keratinizing carcinoma				
Undifferentiated subtype	86	35	51	
Differentiated subtype	13	2	11	
Basaloid squamous cell carcinoma	1	0	1	
Chemotherapy				0.284
No	69	23	46	
Yes	35	16	19	

[a] Median age is 45. Relationship between NLRP3 expression and clinicopathologic characteristics was evaluated using the Pearson Chi-Square test. No significant correlation was seen between NLRP3 and any of the examined clinicopathological features.

Supporting Information Table S7. Multivariate analysis of the association between ASC and local recurrence-free survival of NPC patients

Characteristics	Patients (n = 104)		
	Hazards Ratio	95% Confidence interval	*P*
Age			0.503
> 45	1.00	Reference	
≤ 45	0.77	0.358-1.656	
Gender			0.447
Male	1.00	Reference	
Female	0.68	0.246-1.854	
T stage			0.054
1-2	1.00	Reference	
3-4	2.35	0.986-5.593	
N stage			0.739
0-1	1.00	Reference	
2-3	1.16	0.477-2.840	
Clinical stage			0.421
I-II	1.00	Reference	
III-IV	0.60	0.175-2.074	
Chemotherapy			0.144
No	1.00	Reference	
Yes	1.93	0.799-4.660	
ASC			0.019*
Low	1.00	Reference	
High	0.35	0.145-0.844	

* With statistic significance. The Cox proportional hazards model was applied for multivariate analysis to determine the independence of each prognostic factor. Multivariate analysis showed that the upregulation of ASC was strong independent prognostic predictors for better local recurrence-free survival.

Supporting Information Table S8. Multivariate analysis of the association between caspase 1 and local recurrence-free survival of NPC patients

Characteristics	Patients (n = 104)		
	Hazards Ratio	95% Confidence interval	*P*
Age			0.412
> 45	1.00	Reference	
≤ 45	0.73	0.341-1.554	
Gender			0.312
Male	1.00	Reference	
Female	0.60	0.217-1.630	
T stage			0.041*
1-2	1.00	Reference	
3-4	2.52	1.037-6.058	
N stage			0.630
0-1	1.00	Reference	
2-3	1.25	0.508-3.062	
Clinical stage			0.249
I-II	1.00	Reference	
III-IV	0.47	0.132-1.691	
Chemotherapy			0.254
No	1.00	Reference	
Yes	1.66	0.696-3.936	
Caspase-1			0.019*
Low	1.00	Reference	
High	0.33	0.129-0.832	

* With statistic significance. The Cox proportional hazards model was applied for multivariate analysis to determine the independence of each prognostic factor. Multivariate analysis showed that the upregulation of caspase-1 was strong independent prognostic predictors for better local recurrence-free survival.

Supporting Information Table S9. Multivariate analysis of the association between IL-1β and local recurrence-free survival of NPC patients

Characteristics	Patients (n = 104)		
	Hazards Ratio	95% Confidence interval	*P*
Age			0.747
> 45	1.00	Reference	
≤ 45	0.88	0.408-1.903	
Gender			0.673
Male	1.00	Reference	
Female	0.80	0.290-2.226	
T stage			0.030*
1-2	1.00	Reference	
3-4	2.63	1.100-6.277	
N stage			0.848
0-1	1.00	Reference	
2-3	1.09	0.442-2.697	
Clinical stage			0.322
I-II	1.00	Reference	
III-IV	0.53	0.151-1.861	
Chemotherapy			0.263
No	1.00	Reference	
Yes	1.66	0.684-4.014	
IL-1β			0.002*
Low	1.00	Reference	
High	0.27	0.120-0.610	

* With statistic significance. The Cox proportional hazards model was applied for multivariate analysis to determine the independence of each prognostic factor. Multivariate analysis showed that the upregulation of IL-1β was strong independent prognostic predictors for better local recurrence-free survival.

Supporting Information Table S10. Multivariate analysis of the association between AIM2 and local recurrence-free survival of NPC patients

Characteristics	Patients (n = 104)		
	Hazards Ratio	95% Confidence interval	*P*
Age			0.592
> 45	1.00	Reference	
≤ 45	0.81	0.378-1.742	
Gender			0.397
Male	1.00	Reference	
Female	0.65	0.239-1.764	
T stage			0.040*
1-2	1.00	Reference	
3-4	2.48	1.041-5.915	
N stage			0.661
0-1	1.00	Reference	
2-3	1.22	0.501-2.974	
Clinical stage			0.490
I-II	1.00	Reference	
III-IV	0.64	0.183-2.258	
Chemotherapy			0.278
No	1.00	Reference	
Yes	1.63	0.676-3.906	
AIM2			0.002*
Low	1.00	Reference	
High	0.28	0.129-0.624	

* With statistic significance. The Cox proportional hazards model was applied for multivariate analysis to determine the independence of each prognostic factor. Multivariate analysis showed that the upregulation of AIM2 was strong independent prognostic predictors for better local recurrence-free survival.

Supporting Information Table S11. Multivariate analysis of the association between RIG-I and local recurrence-free survival of NPC patients

Characteristics	Patients (n = 104)		
	Hazards Ratio	95% Confidence interval	*P*
Age			0.287
> 45	1.00	Reference	
≤ 45	0.66	0.307-1.418	
Gender			0.414
Male	1.00	Reference	
Female	0.66	0.238-1.805	
T stage			0.039*
1-2	1.00	Reference	
3-4	2.57	1.048-6.313	
N stage			0.431
0-1	1.00	Reference	
2-3	1.42	0.593-3.393	
Clinical stage			0.334
I-II	1.00	Reference	
III-IV	0.53	0.146-1.921	
Chemotherapy			0.399
No	1.00	Reference	
Yes	1.45	0.613-3.412	
RIG-I			0.023*
Low	1.00	Reference	
High	0.41	0.187-0.881	

* With statistic significance. The Cox proportional hazards model was applied for multivariate analysis to determine the independence of each prognostic factor. Multivariate analysis showed that the upregulation of RIG-I was strong independent prognostic predictors for better local recurrence-free survival.

Supporting Information Table S12. Multivariate analysis of the association between NLRP3 and local recurrence-free survival of NPC patients

Characteristics	Patients (n = 104)		
	Hazards Ratio	95% Confidence interval	*P*
Age			0.723
> 45	1.00	Reference	
≤ 45	0.87	0.402-1.883	
Gender			0.262
Male	1.00	Reference	
Female	0.56	0.205-1.539	
T stage			0.138
1-2	1.00	Reference	
3-4	1.94	0.809-4.649	
N stage			0.157
0-1	1.00	Reference	
2-3	1.93	0.776-4.817	
Clinical stage			0.338
I-II	1.00	Reference	
III-IV	0.54	0.153-1.906	
Chemotherapy			0.368
No	1.00	Reference	
Yes	1.50	0.621-3.618	
NLRP3			0.002*
Low	1.00	Reference	
High	0.10	0.023-0.421	

* With statistic significance. The Cox proportional hazards model was applied for multivariate analysis to determine the independence of each prognostic factor. Multivariate analysis showed that the upregulation of NLRP3 was strong independent prognostic predictors for better local recurrence-free survival.

Supporting Information Table S13. Percentage of TAN-positive NPC

	Inflammasome genes	
	High (%)[a]	Low (%)[b]
AIM2	10.3	5.6
RIG-I	10.3	5.6
NLRP3	12.8	6.2
ASC	12.8	5.3
caspase-1	9.8	7.9
IL-1β	9.9	6.1
average	11.0	6.1
Student's *t* test		0.002*

* With statistic significance. [a] The positive rates of TANs in patients with high expression levels of AIM2, RIG-I, NLRP3, ASC, caspase-1, and IL-1β. [b] The positive rates of TANs in patients with low expression levels of the above described proteins.

Supporting Information Table S14. Cluster of 114 cancer cell lines by expression profile of AIM2, RIG-I, NLRP3, ASC, and Caspase-1

Clusters	Cancer types	Cell lines
1	Lymphoma	ARH-77
		BC-1
		BC-2
		BC-3
		CRO-AP2
		CRO-AP5
		Daudi
		EB-1
		EB-2
	NPC	HK1
		HK1-EBV
		BM1
		CNE-1
		HONE-1
		NPC-TW02
		NPC-TW01
		NPC-TW04
	Brain cancer	CCF-STTG1
		H4
2	Leukemia	BDCM
		BV-173
		CESS
		EM-2
		GCT
	Kidney cancer	A-498
		ACHN
		Caki-1
	Lung cancer	Calu-1
		ChaGo-K-1
		COR-L105
	Breast cancer	HCC1143
		HCC1954
	Cervical cancer	C-4I

		DoTc2-4510
	Bladder cancer	5637
	Brain cancer	DK-MG
	Lymphoma	1A2
	Ovarian cancer	CaOV3
	Pharynx cancer	Detroit562
	Prostate cancer	CaHPV-10
	Sarcoma	A-673
	Ureter cancer	639-V
	Uterus cancer	AN3-CA
3	Lymphoma	DOHH-2<br>EB-3<br>GA10
	Melanoma	A375<br>C32TG
4	Lung cancer	A427<br>Calu-6<br>COLO-668<br>COR-L23<br>COR-L279<br>COR-L88<br>DMS 114<br>DMS 153<br>DMS 273<br>DMS 53<br>DMS 79
	Breast cancer	BT-20<br>BT-474<br>DU4475<br>EFM-19<br>HCC1395<br>HCC1599<br>HCC1937<br>HCC2157<br>HCC2218<br>HCC38
	Brain cancer	A172

	CHP-212
	D283 Med
	D341 Med
	DBTRG-05MG
Colorectal cancer	COLO201
	COLO-320DM
	COLO-320HSR
	HCT-15
	HCT-8
Kidney cancer	769-P
	Caki-2
	CAL-54
	G-401
	G-402
Leukemia	CCRF-CEM
	CEM C1
	CML-T1
	GDM-1
Lymphoma	CA46
	DB
	DG-75
	HD-MY-Z
Melanoma	A101D
	A7
	CHL-1
	COLO-829
Pancreatic cancer	AsPC-1
	Capan-1
	Capan-2
Thyroid cancer	BHT-101
	CAL-62
	CGTH-W-1
Bladder cancer	647-V
	BFTC-905
Cervical cancer	C-33A
	C-4II
Ovarian cancer	COLO-704

		ES-2
	Prostate cancer	22Rv1
		BM-1604
	gastric cancer	AGS
	gestational cancer	BeWo
	Liver cancer	C3A
	Sarcoma	A204

Supporting Information Table S15. Clinicopathologic features of 144 NPC patients used in this study

Characteristic	Number of patients
Age[a]	
> Median	69
≤ Median	75
Gender	
Male	106
Female	38
T stage	
1-2	88
3-4	56
N stage	
0-1	73
2-3	71
Clinical stage	
I-II	26
III-IV	118
Histological type	
Keratinizing carcinoma	4
Non-keratinizing carcinoma	
Undifferentiated subtype	121
Differentiated subtype	18
Basaloid squamous cell carcinoma	1
Chemotherapy	
No	100
Yes	44
TANs	
Positive	13
Negative	127

[a] Median age is 45.

Supporting Information Table S16. Clinicopathologic features of 104 NPC patients used in immunohistochemical staining study

Characteristic	Number of patients
Age[a]	
> Median	50
≤ Median	54
Gender	
Male	79
Female	25
T stage	
1-2	62
3-4	42
N stage	
0-1	60
2-3	44
Clinical stage	
I-II	18
III-IV	86
Histological type	
Keratinizing carcinoma	4
Non-keratinizing carcinoma	
Undifferentiated subtype	86
Differentiated subtype	13
Basaloid squamous cell carcinoma	1
Chemotherapy	
No	69
Yes	35
TANs	
Positive	9
Negative	95

[a] Median age is 45.

Supporting Information Table S17. Clinicopathologic features of 140 NPC patients used in TANs study

Characteristic	Number of patients
Age[a]	
> Median	67
≤ Median	73
Gender	
Male	103
Female	37
T stage	
1-2	86
3-4	54
N stage	
0-1	72
2-3	68
Clinical stage	
I-II	25
III-IV	115
Histological type	
Keratinizing carcinoma	4
Non-keratinizing carcinoma	
Undifferentiated subtype	118
Differentiated subtype	17
Basaloid squamous cell carcinoma	1
Chemotherapy	
No	96
Yes	44
TANs	
Positive	13
Negative	127

[a] Median age is 45.

Supporting Information Table S18. Antibodies used for immunohistochemical staining

Antibody	Dilution	Company
Anti-ASC	100	Millipore
Anti-caspase-1	50	Millipore
Anti-IL-1β	50	Santa Cruz Biotechnology
Anti-AIM2	100	Deciphergen Biotechnology
Anti-RIG-I	100	ENZO Life Sciences
Anti-NLRP3	200	Sigma-Aldrich
Anti-CIITA	50	Santa Cruz Biotechnology
Anti-NLRC4	100	Santa Cruz Biotechnology
Anti-NLRP7	50	Santa Cruz Biotechnology

Supporting Information Table S19. Antibodies used for FACS analysis

Antibody	Fluorescent tag	Company
Anti-CD45	APC	BD Bioscience
Anti-CD45	PerCP-Cy5.5	BD Bioscience
Anti-CD11b	FITC	BD Bioscience
Anti-CD11b	PE	BD Bioscience
Anti-Ly6G	PE	BD Bioscience
Anti-CD3	FITC	BD Bioscience
Anti-B220	PE	BD Bioscience
Anti-CD11c	APC	BD Bioscience
Anti-NK1.1	FITC	BD Bioscience
Anti-F4/80	PE	eBioscience
isotype control	APC	BD Bioscience
isotype control	erCP-Cy5.5	BD Bioscience
isotype control	FITC	BD Bioscience
isotype control	PE	BD Bioscience

Supporting Information Table S20. Antibodies used for immunoprecipitation and Western Blotting

Antibody	Application	Company
Anti-ASC	Immunoprecipitation	Calbiocam
Anti-IL-1β	Western Blotting	Santa Cruz Biotechnology
Anti-p65	Western Blotting	Santa Cruz Biotechnology
Anti-c-Jun	Western Blotting	Santa Cruz Biotechnology
Anti-caspase-1	Western Blotting	Santa Cruz Biotechnology
Anti-tubulin	Western Blotting	Santa Cruz Biotechnology
Anti-Flag	Western Blotting	Sigma-Aldrich
Anti-NLRP3	Western Blotting	Sigma-Aldrich
Anti-p-ERK1/2, and	Western Blotting	Cell Signaling Technology
Anti-p38α MAPK,	Western Blotting	Cell Signaling Technology
Anti-p38β MAPK	Western Blotting	Cell Signaling Technology
Anti-ASC	Western Blotting	MBL International
Anti-AIM2	Western Blotting	Deciphergen Biotechnology
Anti-RIG-I	Western Blotting	ENZO Life Sciences
Anti-actin	Western Blotting	MDBio Inc.

Supporting Information Table S21. Sequence of quantitative PCR Primers

Primer	Sense Sequence
CIITA	AGTTCAGCAGGCTGTTGTGTGA<br>TGGGAGTCCTGGAAGACATACTG
NAIP	CCTTCAGAGCCGTGGTGAAC<br>GGCACTATAGGACCAACTGCTATTG
NOD1	CAAAGGCCTCACGCATCTTAA<br>CTCAGAGATTGATTTGCTGTTCTTCA
NOD2	TCTCCAGGATGAAGGTGTATGTTC<br>CCCTAGGTAGGTGATGCAGTTATTG
NLRC3	CAGGAGCCTCACCAGCTTAGA<br>AGCAGTGAGGGCTGTGTTGA
NLRC4	CCCTTAAGGAGTGGAACTATCCTCTA<br>CCTTTAAATCCTGAGCCAAATCG
NLRC5	TGCCGTGTTGGGTTTGGT<br>TGCTTGTCTTGTCCCCTCTCA
NLRP1	TGGATACGGGAGAGATGAGTAATAGC<br>AGTTTGAGATTAGCCTGAGCAACA
NLRP2	CACCTCTAGACGTGGACGAAATG<br>AAGACTTCTTTACCCAGGCAGATG
NLRP3	TCTGTGTGTGGGACTGAAGCA<br>TACTGATGCAAGATCCTGACAACA
NLRP4	AACTACCCAGCAGGCAACGT<br>AATCAATGGGTGAGAGGTGACAA
NLRP5	CGAGGTCATGAGAGAACCATCTT<br>CACGCGGCGGTGAGA
NLRP6	TCTCAAGGCACCACAAAACAAC<br>CAGACCGCGTCAGGGAGTT
NLRP7	TGAGGGCTTGAGTTACCCTGAT<br>TTGGAGCGCCTCTGAGAGAT
NLRP8	AGGCACCCTCAGTGCAAACT<br>CCCGTCAAAACACCGATTAAG
NLRP9	CTCCTCGATCTGGGCTCAAA<br>TCAACCACAGCTCCCGTATG
NLRP10	GCCATATTTGTCTGCATGATTACAG<br>ACCTGGTTGTATCTGCCATTGA

NLRP11	GCCCACATGCCAAATAAGTCA<br>CAGACTCCCGCCACTGATG
NLRP12	ACAGGAAATGCACTGGAGGATT<br>TGAGGCGGCAGATCTTCAG
NLRP13	CTCTGAAACCACATCGTGCATT<br>GCAAGCAGTTGTCAGATTGCAT
NLRP14	TCAGAGGCTCGGGTTGGA<br>TGCAGATAAGAGCAGAGGAGAGATC
NLRX1	GAGGACGTCAGCCTGGTACTG<br>CAAACACTCGTGGAACCACCTT
AIM2	GCACCAAAAGTCTCTCCTCATGT<br>TAACTGGCAAACAGCGCTTCT
RIG-I	TCAGACATGGGACGAAGCAGTA<br>AGGTACAGGTTTTGGTTTTTCTTGA
MDA5	GGTCTCGTCACCAATGAAATAGC<br>TAACTCCTGAACCACTGTGAGCAA
LGP2	GAGGGCACCCACCATGTC<br>GCTTCCAGTCCTTGAAGACTTTG
P2RX4	CTCTGGCCTGGCACTGCTA<br>CACATATTTATATTTCTTCTCCCGATAGTAGA
P2RX7	AGGATGGTGAACCAGCAGCTA<br>GCAGCCTGGACAAATCTGTGA
Pannexin1	TCCCACTGTGGCTGCATAAG<br>TGAGGAGCAGCTGCGAAAC
ASC	ATCCAGGCCCCTCCTCAGT<br>GTTTGTGACCCTCGCGATAAG
Caspase 1	GAATGTCAAGCTTTGCTCCCTAGA<br>AAGACGTGTGCGGCTTGACT
IL-1β	TCTCCGACCACCACTAC<br>AGCCTCGTTATCCCAT
IL-18	GCTGAACCAGTAGAAGACAATTGC<br>ATCTGATTCCAGGTTTTCATCATCT
GAPDH	TGGTATCGTGGAAGGACTCATGAC<br>ATGCCAGTGAGCTTCCCGTTCAGC
Arginase	GATTGGCAAGGTGATGGAAG<br>TCAGTCCCTGGCTTATGGTT
TNFα	ACCACGCTCTTCTGTCTACT

	AGGAGGTTGACTTTCTCCTG
ICAM1	GAAGCTTCTTTTGCTCTGCC<br>AGCAGTACTGGCACCAGAAT
INOS	CCACCTCTATCAGGAAGAAA<br>CTGCACCGAAGATATCTTCA
CCL2/MCP-1	CAGGTCCCTGTCATGCTTCT<br>GTCAGCACAGACCTCTCTCT
CCL3/MIP1α	ACCATGACACTCTGCAACCA<br>TCAGGCATTCAGTTCCAGGT
CCL5/RANTES	ACCATGAAGATCTCTGCAGC<br>TGAACCCACTTCTTCTCTGG
MRPL32	AGAGGTGCTGGGAGCTGCTA<br>GATGGATGGTCTCTGGACGG
LMP1	ACAAAACTGGTGGACTC<br>GTCTGCCCTCGTTGGA
18S	CAGGTCTGTGATGCCC<br>ATCGGTAGTAGCGACG
Cytochrome *c* oxidase I	AGGAACAGGTTGAACAGT<br>GTTGAGGTTGCGGTCT
EBER1 detection	AGGACCTACGCTGCCCTAGAGGTTTTGC<br>AAAACATGCGGACCACCAGCTGGTACTTG
EBER2 detection	AGGACAGCCGTTGCCCTAGTGGTTTCG<br>AAAAATAGCGGACAAGCCGAATACCCTT
EBER reverse transcription	CCCTTTACATGTTGTGGGTGCAAAACTAGCCA
EBER1 cloning	TGTAATACGACTCACTATAGGGAGGACCTAC<br>GCTGCCCTAGAGGTT<br>AAAACATGCGGACCACCAGCTGGTACTTG
EBER2 cloning	TGTAATACGACTCACTATAGGGAGGACAGCC<br>GTTGCCCTAGTGGT<br>AAAAATAGCGGACAAGCCGAATACCCT TC

Supporting Information Table S22. Sequence of siRNA Primers

siRNA Oligonucleotides	Sense Sequence
RIG-I	GCACAGAAGUGUAUAUUGG
	CCACAACACUAGUAAACAA
	CGGAUUAGCGACAAAUUUA
	UCGAUGAGAUUGAGCAAGA
AIM2	UAUGGUGCUAUGAACUCCAGAUGUC
	GACAUCUGGAGUUCAUAGCACCAUA
	AAACCAUUCACAAUUGUUCCAAGGG
NLRP3	AAAGGAAGAAGACGUACACCGCGGU
	ACCGCGGUGUACGUCUUCUUCCUUU
	UUAGCUUUGGCUUUCACUUCAAUCC
Cathepsin B	UUCUGAUUCGAUUCCACAGUGAUCC
	AUAGUUGACCAGCUCAUCCGACAGG
	UCUCUUUGAUGGUGGGACACUGUGG
p65	GGAUUGAGGAGAAACGUAA
	CCCACGAGCUUGUAGGAAA
	GGCUAUAACUCGCCUAGUG
	CCACACAACUGAGCCCAUG
c-Jun	GAGCGGACCUUAUGGCUAC
	GAACAGGUGGCACAGCUUA
	GAAACGACCUUCUAUGACG
	UGAAAGCUCAGAACUCGGA
p38α/β MAPK	CAAGGUCUCUGGAGGAAUUUU
	GUCAGAAGCUUACAGAUGAUU
	CCGCUUAUCUCAUUAACAGUU
	GUCCAUCAUUCAUGCGAAAUU
ERK1	GACCGGAUGUUAACCUUUAUU
	AGACUGACCUGUACAAGUUUU
	GAAACUACCUACAGUCUCUUU
	GCUACACGCAGUUGCAGUAUU
ERK2	CCAAAGCUCUGGACUUAUUUU
	AAACAGAUCUUUACAAGCUUU
	CAAGAGGAUUGAAGUAGAAUU
	GUACAGGGCUCCAGAAAUUUU
